# QUFIND: tool for comparative prediction and mining of G4 quadruplexes overlapping with CpG islands

**DOI:** 10.3389/fgene.2023.1265808

**Published:** 2023-10-25

**Authors:** Baljeet Kaur, Priya Sharma, Pooja Arora, Vikas Sood

**Affiliations:** ^1^ Department of Computer Science, Hansraj College, University of Delhi, Malka Ganj, India; ^2^ Department of Biochemistry, Jamia Hamdard, Delhi, India; ^3^ Department of Zoology, Hansraj College, University of Delhi, Malka Ganj, India

**Keywords:** G4 quadruplexes, CpG islands, guanine tetrads, G4 quadruplex and viruses, G4 quadruplex prediction

## Abstract

G-quadruplexes (G4s) are secondary structures in DNA that have been shown to be involved in gene regulation. They play a vital role in the cellular processes and several pathogens including bacteria, fungi, and viruses have also been shown to possess G4s that help them in their pathogenesis. Additionally, cross-talk among the CpG islands and G4s has been shown to influence biological processes. The virus-encoded G4s are affected by the mutational landscape leading to the formation/deletion of these G4s. Therefore, understanding and predicting these multivariate effects on traditional and non-traditional quadruplexes forms an important area of research, that is, yet to be investigated. We have designed a user-friendly webserver QUFIND (http://soodlab.com/qufinder/) that can predict traditional as well as non-traditional quadruplexes in a given sequence. QUFIND is connected with ENSEMBL and NCBI so that the sequences can be fetched in a real-time manner. The algorithm is designed in such a way that the user is provided with multiple options to customize the base (A, T, G, or C), size of the stem (2–5), loop length (1–30), number of bulges (1–5) as well as the number of mismatches (0–2) enabling the identification of any of the secondary structure as per their interest. QUFIND is designed to predict both CpG islands as well as G4s in a given sequence. Since G4s are very short as compared to the CpG islands, hence, QUFIND can also predict the overlapping G4s within CpG islands. Therefore, the user has the flexibility to identify either overlapping or non-overlapping G4s along with the CpG islands. Additionally, one section of QUFIND is dedicated to comparing the G4s in two viral sequences. The visualization is designed in such a manner that the user is able to see the unique quadruplexes in both the input sequences. The efficiency of QUFIND is calculated on G4s obtained from G4 high throughput sequencing data (*n* = 1000) or experimentally validated G4s (*n* = 329). Our results revealed that QUFIND is able to predict G4-quadruplexes obtained from G4-sequencing data with 90.06% prediction accuracy whereas experimentally validated quadruplexes were predicted with 97.26% prediction accuracy.

## Introduction

The most widely accepted DNA structure is the classical B-DNA form, which is a right-handed double helix in nature and contains the hydrogen bonds between the nucleobases as described by Watson and Crick in 1953 (Watson and Crick, 1953). Yet, it is evident that DNA is structurally dynamic and can also adopt alternative secondary structures like guanine-rich tetrads (Gellert et al., 1962) and non-guanine rich tetrads (Liu et al., 2018). Guanine-rich DNA strands are capable of folding into four-stranded helical structures, called G-quadruplexes (G4s). The four guanine residues present in the core of a G4 are bonded through the Hoogsteen hydrogen bonds and are stabilized by monovalent cations like K^+^ and Na^+^ to attain a planar form (Gellert et al., 1962; Sen and Gilbert, 1990). A minimum of three consecutive guanine residues is stacked four times successively (called G-tracts or G-runs) with the intervening sequences extruded as loops and taking the shape of a G4 scaffold (Bochman et al., 2012). It is believed that longer perfect G-tracts increase the stability of the G4 structure while unusual G-tracts tend to decrease the stability of the structure (Huppert and Balasubramanian, 2005). On the contrary, new models have predicted the robustness of the unusual G-tracts (Varizhuk et al., 2017; Doluca, 2019). Furthermore, in the early models of G4 structures, it was assumed that loop lengths up to 7 bases could form stable quadruplexes (Huppert and Balasubramanian, 2005). However, it has been since then observed that the quadruplexes with several long loop lengths (up to 30 bp) could also exist (Guedin et al., 2010). It has been shown that RNA can also adopt this type of non-canonical structure under physiological conditions (Davis, 2004). G4s can be unimolecular or multi-molecular and can attain a variety of topologies arising from different combinations of strand direction, length, and loop composition and both intramolecular and intermolecular G4s can also be observed (Spiegel et al., 2020). During the last few years, there has been a lot of growing interest in the scientific community in exploring these G4 structures and their regulatory roles among biological processes (Maizels, 2006; Rhodes and Lipps, 2015; Hansel-Hertsch et al., 2017; Spiegel et al., 2020). They are widely distributed in prokaryotes, eukaryotes, and viruses and play key roles in regulating several physiological and pathological processes. Some of the biological processes known to be regulated by G4 structures include DNA replication (Valton and Prioleau, 2016), damage and repair system (Fleming et al., 2017), genomic instability (Wang et al., 2019), gene expression (Cave and Willis, 2022), chromatin rearrangement (Reina and Cavalieri, 2020), and viral latency (Ruggiero and Richter, 2018). Their role in diverse biological processes renders them interesting potential therapeutic targets (Ruggiero and Richter, 2018; Carvalho et al., 2020). Different studies in various organisms have revealed that G4 secondary structures are located in a non-random manner within genomes and tend to cluster in particular/functional genomic regions like telomeres (Sundquist and Klug, 1989), promoters (Siddiqui-Jain et al., 2002; Dai et al., 2006; Fernando et al., 2006; Xu and Sugiyama, 2006), and untranslated regions (UTRs) of mRNA (Huppert et al., 2008). The role of G4s is associated with several diseases including cancer (Varshney et al., 2020), neurogenerative disorders (Wang et al., 2021; Vijay Kumar et al., 2023) and rare genetic disorders including fragile X syndrome (Asamitsu et al., 2021).

Initial *in silico* approaches for the prediction of putative G4 structures on a genome scale were based on the experiments conducted biophysically (Huppert and Balasubramanian, 2005; Todd et al., 2005). The ongoing era of next-generation sequencing has made whole genome sequencing relatively easy and affordable thereby creating a wealth of genomic data which can be used to obtain a bird’s eye view of the cellular processes. Using techniques like rG4-seq and G4-seq, scientists have developed the transcriptome-wide (Kwok et al., 2016), genome-wide (Chambers et al., 2015) experimental map of G4s in humans, and recently, the more exhaustive whole-genome landscape of G4s in 12 species (Marsico et al., 2019). Several improved computational algorithms have been developed by employing the G4-seq dataset to train a machine learning model for the characterization of G4 structures on a genomic level (Garant et al., 2017; Hon et al., 2017; Sahakyan et al., 2017). Notably, the vast majority (80%–90%) of the G4 structures predicted by these improved computational approaches were confirmed to exist in genomes by the G4-seq approach (Marsico et al., 2019). A powerful tool called pqsfinder (Hon et al., 2017) provides a flexible framework for its users and allows them to define the custom criteria for scoring and matching. It allows the user to input up to three imperfections (mismatches, bulges in G-runs, and/or long loops >9 nt) in a single sequence of DNA or RNA and has the advantage of assigning a score to each predicted G4 sequence. The scoring scheme emphasizes the stability of the predicted structure because it gives a bonus score to the perfect G-tetrad stacking and a penalty score in case of mismatch and bulges. Quadron (Sahakyan et al., 2017) is a machine learning (ML) model based on a tree gradient boosting machine and trained on the G4-seq data for the human genome, which allows the user to predict G4 structures in DNA as well as RNA sequences. G4RNA screener (Garant et al., 2017) applies an ML model based on an artificial neural network and trained on experimentally validated G4s from sequences deposited in the G4RNA database. It allows the user to predict G4s in RNA sequences only and incorporates the cG/cC and G4 hunter algorithms for better or comparable outcomes. Additionally, ImGQfinder (Varizhuk et al., 2014) is another tool where a user can predict G-quadruplexes. This tool allows one mismatch or bulge in G-tract. QPARSE (Berselli et al., 2020) is a graph-based search algorithm where users can look for monomeric and multimeric quadruplex forming sequences and G4s with long, hairpin loops. Users are allowed to enter the query sequence of a maximum 10,000 bp length or upload a fasta file of a maximum 15 Kb size. G4-iM Grinder (Belmonte-Reche and Morales, 2020) looks for G4s and i-Motifs within a given DNA or RNA sequence. It has three distinct methods: the G4 search engine with 13 customizable functions (for example, showing G4 on both strands, loop sequence, size, *etc.*), G4 qualification functions, and quantification functions. It incorporates cG/cC and G4 hunter algorithms to evaluate better results. The continuous progress in literature providing evidence on the *in-vitro* existence of G4 structures containing more than four G-tracts (Phan et al., 2005; Omaga et al., 2018) and G4 structures containing all the possible tetrads, A:T:A:T tetrads and bulged nucleotides in one single structure (Liu et al., 2018) still remained to be incorporated into the search algorithm.

Anotpther interesting role of G4 structures is to influence the methylation at CpG islands (CGIs), which are guanine-cytosine-rich regions and are usually hypomethylated. The CGIs are widespread at the promoters of housekeeping, tissue-specific, and developmental genes and co-localize with G4s in these actively transcribed regions for gene regulation (Jara-Espejo and Line, 2020). Recently, it was proposed that the G4 structures protect the CGIs from methylation by sequestering and inhibiting DNA methyltransferases and hold an important place in epigenetic control mechanisms (Cree et al., 2016; Mao et al., 2018). As both G4s and CGIs are tightly associated with actively transcribed regions, their accurate identification in the genome is of great significance.

Several computational tools for the identification of CGI in a given DNA sequence are accessible to users nowadays. Three of the widely used conditions for CGI analysis are as follows 1) moving window should be of 200 nucleotides, 2) GC content higher than 50%, and 3) CpG O/E (Observed/Expected) ratio higher than 0.6 (Gardiner-Garden and Frommer, 1987). Some improved versions for CGI identification are also available which include the additional parameters (Larsen et al., 1992; Ponger and Mouchiroud, 2002; Takai and Jones, 2003; Hackenberg et al., 2006).

The presence of stable and conserved G4s in all known human viruses and their variants has been successfully presented by Lavezzo et al. (2018) but their analysis is mainly based on the reference genomes (RefSeq). Additionally, the analysis pipeline has failed to provide any criteria where user-defined sequences can be handled. It is evident that mutations in viruses have played a major role in evolution (Moelling and Broecker, 2019) enabling them to evade host immune responses efficiently (Xia et al., 2018). Several studies related to mutations in quadruplex-forming structures have shown that mutations in G4s may hinder normal cellular activities (Khristich and Mirkin, 2020). Therefore, understanding the potential effects of mutations on quadruplex-forming structures warrants further research.

All quadruplexes and CGIs search models have limitations despite the advancements in the field as none of the tools has been explicitly designed to detect and analyse all possible G4s. Keeping in mind that G4 forming sequences within a genome harbour CpG sites, we have developed a web-based server, QUFIND (QUadruplex FINDer), where users are allowed to predict either the G4 structures or CGIs and G4 structures simultaneously, in a wide range of organisms. This is the first-ever tool that allows users to find the G4 forming sequences within CGIs so that the user can analyse G4 secondary structures in context with the CGIs. The web server interfaces with ENSEMBL’s REST API and NCBI API to mine CGIs and/or G4 secondary structures. It provides options to search the entire ENSEMBL and NCBI databases in order to retrieve the desired nucleotide sequence entries for analysis. The web server is organized in such a way that users are free to choose as many possible parameters by themselves. The web program is divided into three modules: QUFINDU which allows querying CGIs and/or G4 secondary structures in all the species for which the sequences are available in the ENSEMBL and NCBI database, QUFINDV which allows querying G4 secondary structures in viruses and their variants and lastly, QUFIND which allowed CGIs and/or G4 secondary structures to search in user-provided sequences. QUFINDV offers an interactive graphical representation of the G4 sequences in viruses and their variants for comparative studies on a single screen. The program is also designed to handle the analysis of non-guanine-based quadruplexes as well as the prediction of overlapping G4 structures among the CpG islands.

## Methodology

### Definition of model

The design of this tool is focused on the mining of G4 secondary structures contained within the CGIs. For this study, CpG regions were defined as the moving segment of DNA or RNA of 200 bp constant length, GC content in that region should be greater than 50%, and the O/E (Observed/Expected) ratio should be higher than 0.6. Observed CpG is the number of CpG dinucleotides in the segment and expected CpG is calculated by multiplying the number of “C”s and the number of “G”s in the segment and then dividing the product by the length of the segment (Gardiner-Garden and Frommer, 1987). The typical G4 secondary structures are identified using the following configuration:

$$G_{x}N_{1-y}G_{x}N_{1-y}G_{x}N_{1-y}G_{x}$$

In the motif represented above, G_x_ is the continuous stretch of guanine (G) bases repeated “x” times [where x ∈ (Gellert et al., 1962; Bochman et al., 2012)], called G-tract also referred to as stem length and N_1-y_ represents the loop which ranges from one to “y” and y ∈ (Watson and Crick, 1953). The G4 motif is composed of four G-tracts and three loops but some defects like bulges and mismatches also exist in G-tracts, and give rise to atypical G4 secondary structures. In these, continuous stacking of guanine bases is interrupted by non-guanine bases in the form of bulges (Mukundan and Phan, 2013), and substitution of a non-guanine base for one of the guanine bases in a G-tract can also occur (Tomaško et al., 2009). The other type of mismatch is the vacancy of a guanine base in one of the G-tracts (Li et al., 2015). The users are provided with an option to search for these atypical G4 secondary structures. The G-tract configuration for atypical motifs changes to G_x_DG_n-x_ (in case of bulged tracts) and G_x_DG_n-x-1_ (in case of mismatched tracts) where 2 ≤ x ≤ n, “n” is the maximum length of the G-tract and ‘D’ is the defect. In this section, users are required to select the type of defect and the number of defects. We have limited the number of bulges up to 5 and the number of mismatches up to 2. The strategy to mine typical and atypical G4 secondary structures is based upon a “regular expression” which is purely dependent upon the selection of parameters and search within a query sequence. The algorithm mines overlapping or non-overlapping CGIs/G4 secondary structures in a given sequence. Overlapping G4 secondary structures may contain multiple internal G4s but non-overlapping G4 secondary structures will not have coinciding G4 coordinates. The same algorithm is applied to search for other tetrads including T, A and C.

### Architecture and features

QUFIND web server backend is written in Python programming language using Flask microframework and all visualizations are generated using Matplotlib library. The front end is written using HTML, CSS, JavaScript, and the jQuery library of JavaScript. The web server interfaces with ENSEMBL’s REST API and NCBI’s E-utilities to fetch a nucleotide sequence in FASTA format from the database and analyze it for the presence of quadruplexes in an overlapping or non-overlapping model. This keeps the server up to date with ENSEMBL and NCBI’s latest release. The individual modules of the web server are described in the following subsections.

### QUFINDU (QUadruplex FINDer for UserID)

**FIGURE 1:**
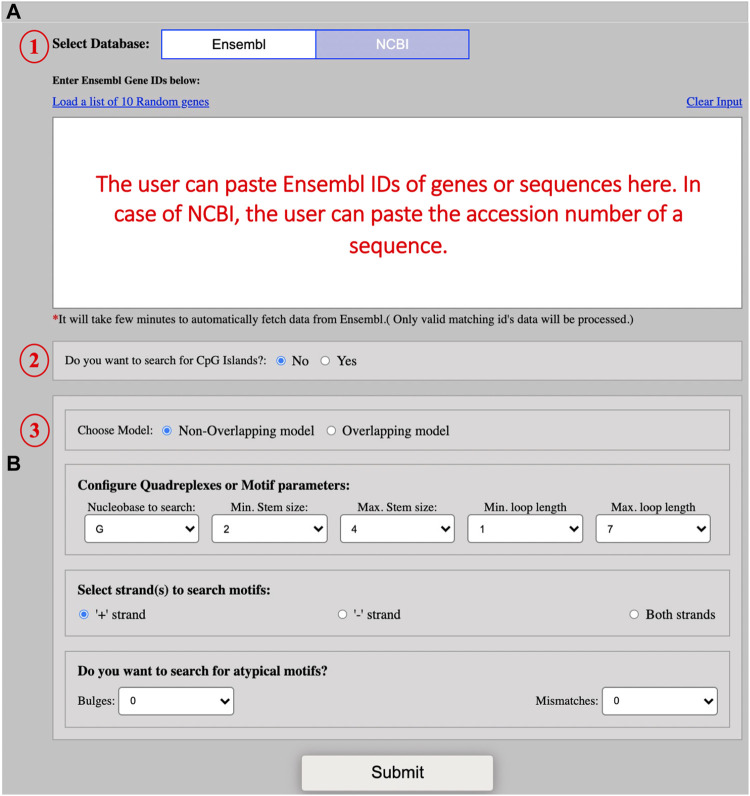
Screenshot of example input. **(A)** The screenshot shows the input interface for the QUFINDU module. The user needs to select the database (1) (“Ensembl” is set by default) and then paste multiple Ensembl IDs of genes in the text box. In the case of NCBI, a single accession number of a sequence can be pasted in the provided box. The users can click on “Yes” if they want to search for CpG islands first (the CpG islands option is not selected by default) (2) and then select the search model to mine G4 motifs: Non-overlapping or overlapping (3). **(B)** The screenshot showing the different parameters for G4 motif configuration. The users can directly click on the “Submit” button with the default parameters or can change the parameters according to their interests. The different parameters include Nucleobase to search, minimum stem size, maximum stem size, minimum loop length, maximum loop length, and strand option. The user can also choose to search for defects in the G-tract or stem. Defects are of two types: bulges and mismatches. Both types of defects cannot be chosen simultaneously.

This model allows users to mine G4 secondary structures in all species available in the two databases, ENSEMBL and NCBI. By default, the ENSEMBL is selected and the user can mine typical or atypical secondary structures by providing the ENSEMBL ID of the interested gene or sequence (Figure 1A). The user can change the database for fetching the sequence. In the case of NCBI, the user can enter the accession number of a sequence. The server is set to search for G4s only by default but the users can opt to search for G4s along with CGIs alike QUFIND. The server can mine overlapping or non-overlapping CGIs/G4s (G4 secondary structures search occurs internally in case of CGIs) for both the searching options. The G4 secondary structure motif configuration parameters in this module are the same as described in QUFIND. Various options to predict secondary structures are represented in Figure 1B.

**FIGURE 2:**
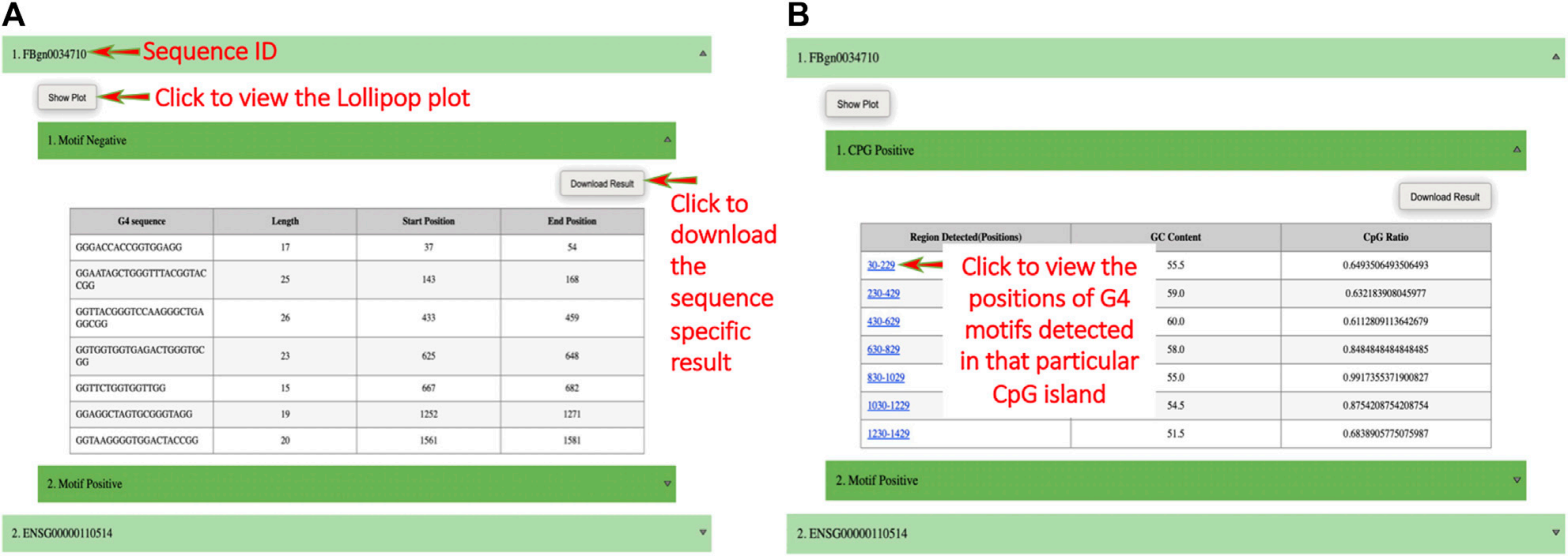
Screenshots of example output. **(A)** The result page of QUFINDU/QUFIND module. The result page shows the gene or sequence IDs, clicking on which a table is shown for the selected strand (s) (i.e., “Motif Positive” or “Motif Negative” or both). The table contains all the information obtained for mined G4 motifs. The users can click on the “show plot” button to view the plot and a click on “Download Result” will download the sequence and strand-specific results. **(B)** The result page of QUFINDU or QUFIND module if the “CpG islands” option is selected. Every ID contains a sub-option “CpG Positive” or “CpG Negative” or both depending on the selection made by the user, clicking on which a table is shown containing detected regions of CpG islands, their GC content, and CpG ratio. In this case, the G4 motifs mining process occurs internally and hence the detected regions of CpG islands are provided with a hyperlink. Clicking on the hyperlink opens an image showing the start and end position of G4 motifs contained within that CpG island.

On submission, a result page is displayed on which the user can click on any sequence ID to view its result in the form of a table and can download the sequence-specific result. The user can also click on the “Show Plot” button to view the image (in the form of a “Lollipop” chart) representing G4 secondary structures with their annotated length and position (i.e., length, position) (Figure 2A). If the CpG islands option is selected then a result page is displayed on which the users can click on “CpG Positive” to view the table containing CGIs rich region positions, their GC content, and CpG ratio value and can download the CGIs-specific result. The position of G4 secondary structures that lie within CGIs can be found in the form of an image by clicking on the hyperlink given on CGIs positions (Figure 2B).

### QUFINDV (QUadruplex FINDer for viruses)

**FIGURE 3:**
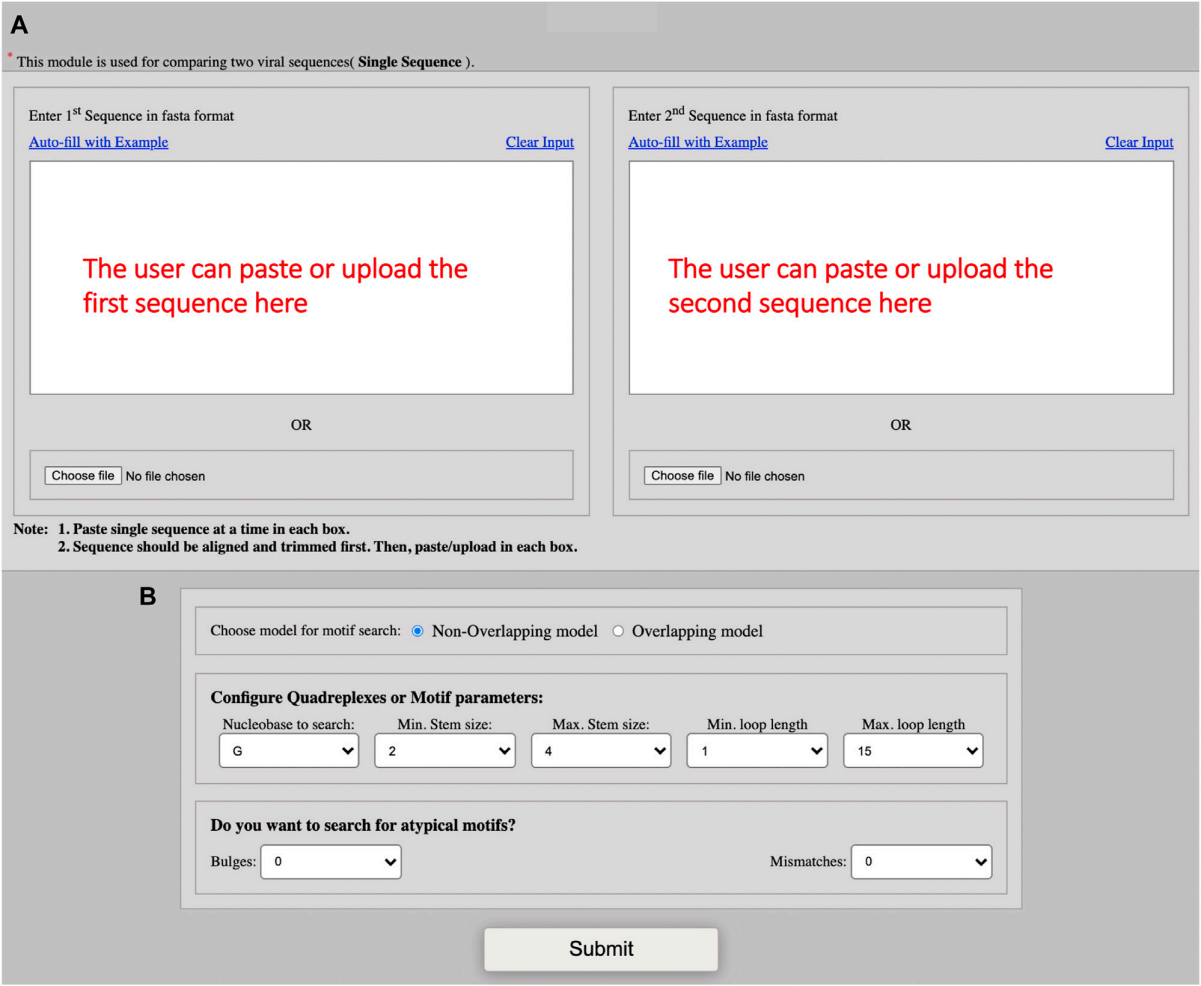
Screenshot of QUFINDV module. **(A)** The figure shows the input interface for QUFINDV module. The user can paste or upload the first sequence in the box placed at left and second sequence in the box placed at right. These two sequences should be equal in length for comparison. Then, select the search model to mine G4 motifs: Non-overlapping or overlapping **(B)** the screenshot showing the different parameters for G4 motif configuration. The users can directly click on ‘Submit’ button with the default parameters or can change the parameters according to their interest. The different parameters includes: Nucleobase to search, minimum stem size, maximum stem size, minimum loop length, maximum loop length and strand option. The user can also choose to search for defects in the G-tract or stem. Defects are of two types: bulges and mismatches. Both types of defect cannot be chosen simultaneously.

QUFINDV allow querying quadruplexes in viruses and their variants. The appearance of mutation in the genome of a virus leads to the generation or disruption of the quadruplexes. Hence, the detection of unique G-quadruplexes in the variants of concern should be explored. The users need to first align the two query sequences using any sequence alignment tool (e.g., Clustal W) and then trim the overhangs found in the respective sequence. The trimming step is performed to make the two sequences equal in length. After trimming, users are required to paste/upload the first query sequence in the two text boxes for comparison (Figure 3A). The user then can choose the model of an algorithm for fetching the G4 secondary structures, i. e., either overlapping or non-overlapping. The motif configuration parameters in this module are the same as those used in the QUFIND (Figure 3B). This module offers an interactive graphical representation of the G4s in viruses and their variants for comparative studies on a single screen.

**FIGURE 4:**
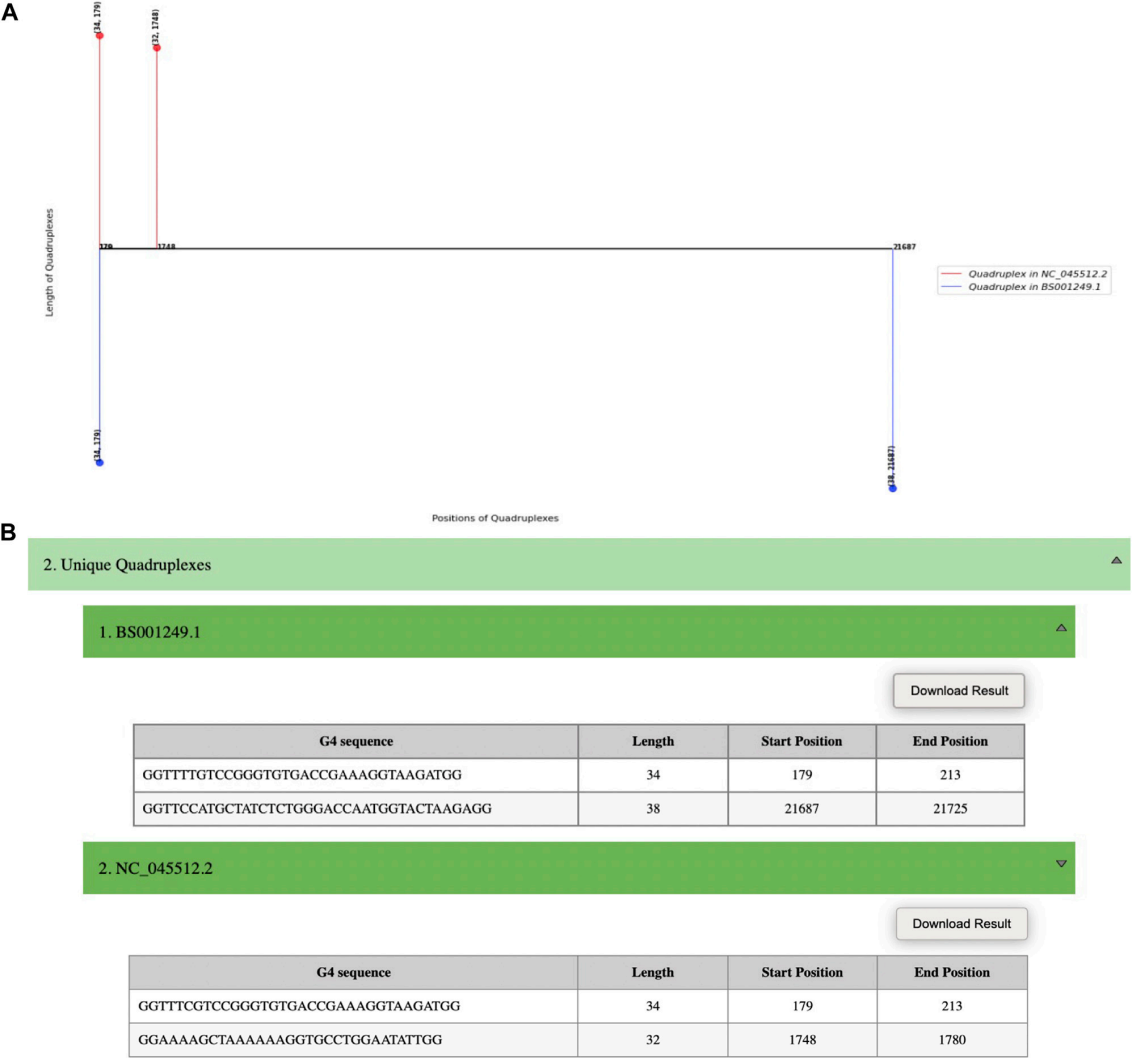
Screenshot of QUFINDV output page. **(A)** A “lollipop” plot representing the unique quadruplexes with its annotated length and position (i.e., length, position). The stems in the +*y*-axis represent the quadruplexes in the first viral sequence (NCBI Ref seq ID: NC_045512.2) and the stems in the–y-axis represent the quadruplexes in the second viral sequence (NCBI ID: BS001249.1). **(B)** Detected unique quadruplexes information in the form of a table for each ID.

On submitting a query, an image representing unique quadruplexes is displayed with its annotation (Figure 4A) and a file containing information related to the detected quadruplexes can be easily downloaded (Figure 4B).

### QUFIND

It is the core search module of the web server, that is, responsible for performing G4 quadruplex search in custom sequences. It allows users to search for their query sequences by either uploading sequences containing files in FASTA format or pasting multiple FASTA formatted sequences. The users can either choose to run CGIs with G4s or G4s alone. The server can mine overlapping or non-overlapping CGIs/G4s (G4 secondary structure search occurs internally in case of CGIs) in both search options. The users have the flexibility to choose the nucleobase G, C, A, or T (G is used by default). The users can then define the minimum and maximum stem length of Gs required per G-tract, the minimum and maximum size of the loops, and the maximum number of bulges/mismatches allowed. The default value for the minimum and maximum stem length of Gs is set at 2 and 4 and the minimum and maximum size of the loops for the default condition is set at 1 and 7. The user can select up to 5 maximum bulges or up to 2 maximum mismatches. The presence of bulges and mismatches at the same time is not allowed.

Upon the submission of a query, a progress page is displayed and users can bookmark it to access the results of their submitted sequences later on. After the analysis is complete, a result page is displayed where users can find the information related to all the submitted sequences along with the visualization. By clicking on any sequence ID, its corresponding nucleic acid secondary structure can be found and its representative figure shows the position of the mined quadruplexes in the sequence. The users can also download results for all the sequences and/or only specific sequences in CSV format. In the CSV file, the sequence, and length of mined quadruplexes is given along with the start and end position.

### Evaluation of QUFIND

**FIGURE 5:**
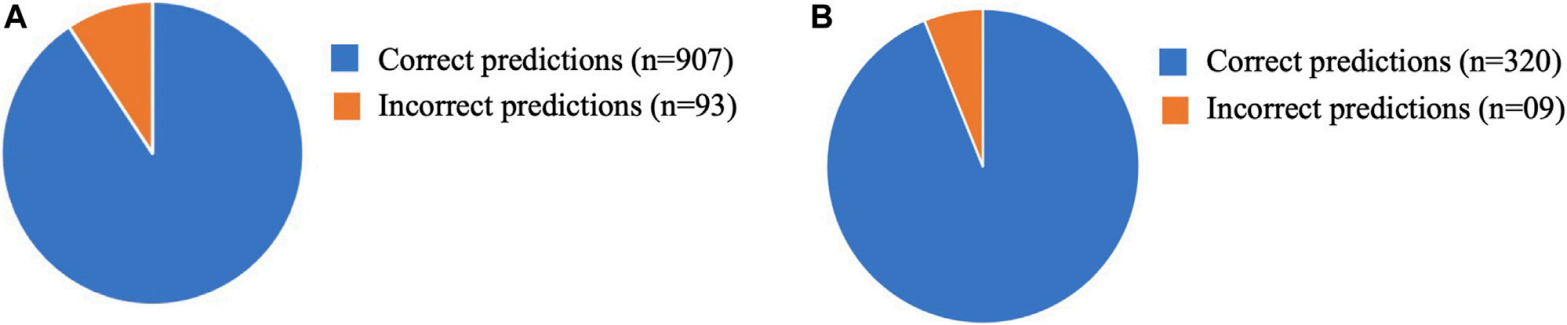
Graphical representation of the performance of validation data. **(A)** Prediction accuracy for high throughput G4 sequences (907/1000 = 90.70%) and **(B)** Prediction accuracy for experimentally validated G4 sequences (320/329 = 97.26%).

The model was tested on 1000 positive sequences (Supplementary Table S1) obtained from the high-throughput dataset in which quadruplexes can be formed (Chambers et al., 2015; Klimentova et al., 2020). Another dataset consisting of experimentally validated 329 sequences (Supplementary Table S2) was prepared through literature mining. The validation of the model was performed by simply passing the G-quadruplex containing sequences which were obtained via 1) high throughput sequencing (*n* = 1000) and 2) experimental validation (*n* = 329) through the tool. The number of correct sequences is represented as the percentage of the total sequences and is presented as the prediction accuracy. The tool was able to predict G4 quadruplexes obtained from a high-throughput dataset with 90.06% prediction accuracy whereas experimentally validated quadruplexes were predicted with 97.26% prediction (Figure 5).

## Conclusion

QUFIND is the first web server that allows users to find quadruplexes either without or within the CGIs. The tool provides an opportunity for the users to analyze G4s in the context of the CGIs. In addition, the server is able to mine G4s in viruses and their variants. This web server is connected with ENSEMBL’s REST API and NCBI’s e-utilities to get the latest gene models or genomic assembly of any organism to detect the presence of secondary structures in them. This server also provides an interactive graphical representation of the mined secondary structures and the results can be downloaded in a convenient format.

The methodology of secondary structure mining allows flexible customization of stem length, loop length, nucleobase, and inclusion of defects. Generally, motifs with three or four nucleic acid-tracts and a loop length of 7 nucleotides are considered to be more stable but due to continuous developments in literature, unusual G4-forming structures can also be seen. Hence, QUFIND is meant to be a flexible and comprehensive tool for investigating G4s.

## Data Availability

The original contributions presented in the study are included in the article/Supplementary Material, further inquiries can be directed to the corresponding authors.
