# Supplementary material for: QUFIND: tool for comparative prediction and mining of G4 quadruplexes overlapping with CpG islands
Source: Front Genet. 2023 Oct 25;14:1265808. doi: 10.3389/fgene.2023.1265808 (PMC10634401; doi:10.3389/fgene.2023.1265808)
Supplement: Supplementary file 1 [file Table1.docx]

Supplementary table S1 shows all the positive sequences obtained from the G4 high throughput dataset (PMID 33193663).

* Denotes sequence with no predicted quadruplexes.

>P1

AGTATGGGCTTATCAGACTAATGGTGAAGGGTGGGGTGGGTAGAAAGGTTTATGTCTTCACAGAAGGAATCTGTTCCTTCACTTTTCAGGTTTTCTTGTCATTGTAACCATGTCACTTTC

>P2

GAGGAGCATGGTGGCCGTCCTACCAGAGGGAGGGGTGGGGTGGAGGGAGCTCACCTGACAGGCTTTACCTCTCTC

>P3

TGGTATCTGAGTTCACTCCACGTAGAGTCTGAATTGTATGTCTTGGTGTCTTGAAGAGTTTGATTTGATGATTTTCAGAGAGGGGTGGGGGGGGTGTGGGGTCCCTAATTTGTTTCTGTAGGTAGGAGCACTCGA

>P4

AAAGGGGTACAGGAACAGGTGTGACAGGAGAGAGGGGAGAAAAATTGAAAGGAGAGAGGTGTGAAAGGAGAGAGGGTCAGGAAATTGAAAGGAGGTGCGTAGCAG

>P5

GCCCCAGTACAGGGGAACACCAGGGCCAAAAAGGGGGAGTGGGTGGGTAGGGGATTGTGGGGGTGGGTATGGGGGACTTTTGGGATAGCA

>P6

TAGAGGACTCCAACGGACAAAGGTAAGAAAAACGACCGGGAGCCCCGAAGTCAGGAAAAGGGATTGGGTTATGATCTAGTCCAACGGGAGAAGAAGGGGGGTGGGGGTAGGTGCAAGGGA

>P7*

ACTATCTCTAGACGTCAATGGTGGTGTCAATAAAAGTCAAGTCAACCGAACGAACAAACGAGAGAGAGAGAGAGAGAGAGAGAGAGAGAGAGAGAGAGAGAGAGAGATTTGGGGTCACAG

>P8

GTAAAAACTTACGAATCTAAGTAAAAGTAAAAGAAGGAAATTTTTTCAATAAGAAGAGAGTATATGGTATAGGGTTGGTGTCGGAGGGGAGACAGGTAAAGAAGGTCTAGGGTAGGGACG

>P9

CTATCTACCAGGAAGTGCAGGGTGGTGGGAAACTCTCATGGATTTGGGGATGGTGGCAGACATAGGCAAAATAGAAGTTCATGGGAAAACCTGAAAATGCCTGAAGAGTCTGCCTCAAACCAACTGTTCTTTTCAGGGGGCACCTGGGCCTCGGGGGCCTGTGGGTGAGAAGGGGGACCAGGGAGATCCTGGAGAAGACG

>P10

AAGCTCGGAGTCTCGGGGGAGAGTATTTGGGGGAGGGAGTAGCTAGGCGGGCTTCCTTCTTTGTGTACCTCAGAAAACTGTCTTAGCAAGTTACACTCCCAGGTCTAAAAAAAAATGAGGTAGTGCAGACACTTAGGATTTCAAATCTGC

>P11

TGTTTTATATTTAGGCTTTGGTGGGTGGGTTGGGAGGGGGTAAATAGTTCATGAGGCTTTTCTAAGAAATTGCTGACAAAGCACTTTTGGATGATGCTATCCCAGCAGTGGGGTGGGGAGAAAGGATAATATAAC

>P12

AGGGAGTGGGTGGGTGGGGGAGCACCCTCATAGAAACAGGGAGAGGAGGGAGGGAATAGGGAGCTTCCAGAGGGGAAACCAGGAAAGGGAATAACATTTGAAATGTAAATAAATAAAATATCCAGTTAAAGAAAAAAAATTTACTTGCTATGTTCAAGAGAAAAA

>P13

CTTTCCATTCCGGGCTTGGGGGCGGGGGGAGGGGGGCTCATGCCTGTTTCCACTGTTTTACAGGTGGTAGCCCGGTGGCCTGAAGCTTGACCAGATGGAGTCCTTTCAGCACATGTCCAC

>P14

AGAAAAGACTTATGTGAACATTTAAAAAAAAATGACGGAAGTGTTAAGAGTAATAGTAGAAACCAGAAGAGTAGGGAGGAGGAGGAGGGAGGGGGTGGGGTGTAGAAAGGGTTCTCGGGACACTAGAATTTTGTT

>P15

CCTGATTCCCCTGTTCACCCTACTGATTCTCTAGAAGGGTCTACCTTCCGGACCGTACCCACGTACACCTTCATAAGTACCTCCTCTGAAGTCGGCGGGTAGTCACGAGACGGGGCGGGGAGTCGCCAACCATTC

>P16

CTGTTAGGGACCTTGGTTCAATCCAATGGTTGGCTTCGAGGGGGGGGAGGGGTTGCAGGGAAGGAACCAGGTAGGGGGATAACATGTGAAATTTAAATAAAGAAAATAACCAATAAAAATTAATTCAAGTCAATAAATCAAATAAGGAGT

>P17

CGTCAGGTTCCTGTACGTCCTCTTAGAGTCCTACCCGATGTTCTCCCTGTCTCTCAGAGAAACAGGGGTACGTATTGTTCAAGGAAGGGAGTTGAAGGTGGAGTTGTGTGTTATCAAAGA

>P18

TGTGTGTGTGTGTGTGTGTGATATTTTAAGAGGAAGGGGGAGAGTATTAAGACTCCATTTTAAGGCTCCTGAGAACTTATTAGGTAATTTGACTAATTATTTGATGGGAAAAGCCAGCAT

>P19

TGGAGGGGAGGGAGGGTTGAGAAAGGTTCTCATGTAGCCCAGGCTGGCTCTGAATTCTCTATGCAACTAAGGGTGGTCTTGAACTTTGGC

>P20

AGATTTTTAAGGTGTACCTGTGTAGGGGTGGGATCTGGGTTTAAGGTGGTTCAGAAAATGACATGGAGAGTCAGAGTTAGGGAAGAAATGGCTGGTGTGTTTTATAAAAAGAAAGAAAGAAACAGGAATATATTT

>P21

GTTCATGTTTCAGGTGGTCGACCCCGACTCGAGTATAGACTTACTCTTGTACGGGGGACAGGGGTAACGAAGGGTCGTCTGGAAGACAGA

>P22

AGGTCGGAGTTCGTTTTAACAAGAGCACACACGCAAGCACACACACACACACACAGAACACGTCCTCATGTACGAGACATTTTACAGGGAAGTATCGCCAGGGGGGTGGGTAGGGGCACATCACTGGAGTCACCC

>P23

GATGTGGTGGGATGGGAGGGGAAGGGAGAGAGAGACTCTTCCAGGGAATGGGACCTAATTTCCTTTGCCTAGATCTCACCCCTTACAGGACTCACCAGATCCCAG

>P24

GAGATTAAAACGAAAAGACAATAATAATTAAAATAACCGATAAAAGCAATAAATGTAAAGTTTACAATAACGGTAAGGGTCAAAGGGAGAGAGGGTCTTTGTGGGATAGTGTAGAAGGGAGGGGGACGAAGACACTACCATAACGAGGTGGGTGGGTGGGTGAAGGTGGAGGGACGGGAGGTAAAGGGATGTGACCCCGT

>P25

ATTTTGGAGTTACAGTTTAGTTGAAGATGTCTTCTCTTTCCGTACGAGGAGACGTTTGTAGAAGGGAGAGGGGGAGAGAGGGAGGGAGAGAGAGGGAGAGATTAGAGGGATGGATCAACAGAGAAGGGGGGAAGTTGAGGGGTTGAGTGGGAGTGGAGTGTTCGT

>P26

TCGTTGTCTCAGTTGAATGGACCTGGGAATCCCGAGAGTCTCAGACTTGGTGGTTGGTTTCTTATATGTGTCCGACCTGGTGTGGGGGGGTGTGTGTGTGTGTGAGTGTCGTGTACACGT

>P27

GCTCGGATGTGATCTAGTATTTTTACCAGAGGTCTCTTTCACCCGAGTACTATTTCACCAGAGTGGAGGGGGACGGGGGGTAGGAATATTTCGAAGACAGGGACC

>P28*

CGTGTGTGTTTCTGAGAGAGAGAGAGATTGAGACTCAGACAGACAGACAGACAGACAGACAGACACTAAGATTCTGGCAGTCTCCCAGAGTGACTGGCAAAAAAA

>P29

GGAAGGGGGCATAGCTGGGGAGATAGTCTGCCTTGCAAATGTGAGGACTTAAGTTTTGATGCTAAAAACCCAGTTAGTTTTATGTCTGTGAAATCCACCTTAATAGTTTTTTTAAGTGAC

>P30

CAGTCGAGATTTATATGCGTTCGCATCAGAAGAACCTTCCCGGTCTCTCTTGAAAACAACAGAAACTGAGGGTGGGTCTGATTCTTCCTCTATCACAAACAGTAAAAGGGGTGTTCTTGAGTAGGGACTTACTTTTCCGTTAGATGGTTCCGGGGAGGGGATGGGGTCCAGTTCGGGGTGGGGATTGACGTCAGTGAGGG

>P31

ATATAAATCCCTTCCAATCAAAAAAAAAGTAAAAATAATCTATAAAAGAAATAAATGTAAAGTTTAAAATAGGGGAAAGGAGTAAAGGGGAGGCTTTTGGAGGGGTAGGGTAGGGAGTTGGGGGACGAGAGGTTGGGTGCGTGAGGGTGA

>P32*

AGAAGAAGAAGAAGAAGAAGAAGAAGAAGA

>P33*

TACACATCTCTGGTCTCCAGTCATAGTCCATAGAAGGAGTTATAGAGAAGAGAAAAGAAGAAAAAAGAAGAGAAAAGAAGAAGGAGAGAGAGAGAGAGAGAGACAGAGAGAGAGACAGAGAGAGAGAGAGAGAGAGAGAGAGAGAGAGAGAGAGAGAGAGAGAGAGAGAGACACACACACACACACAGAGAGAGAGAGGG

>P34

AGAGACTTAGAGGGAATTAGAGAGAGAGGGAGGGAGGGAGGGAAA

>P35

TGGTGATGGTGGTAGTGGTGGTGCTGGTGCCTTTTTTGCCTTCTACTGTAAGTGACAAGAATCTTTGATTTCACCATATAGCTCATGATG

>P36

CCCCATTAGTCCTTCCCCCTATATTAAATTTTACATTTACTTATTTTAGTAATTATTATTATTATTGTTATTATTGAGGATCGTAGTGGAGGAGGAGTCAAAGAAGGTCGGAAGGGAATT

>P37

CCCCTTCCTTGCTACTACGTTGACTCCTCATACAGAAACAGGTCATACCGGTCAAGAGTCCGACGATCCTCCAGACCCGATGGTGGTCCGGGTGGAGTGGGACGGTGGTGGGTGACTAACAGGACAACCCCTCCG

>P38

TAATAATAATAATAATAATAAAATGAAATGAAAAACCAAGTCAAATTACACGTCCCCTTTGCACCGTAGGGACAACAACTTGTCAAGGAGGGAGGGAGGGATGGGATAAGTCAGACTTCGAGTGTAGGAGTGTGA

>P39

TGAAAAAATAGCTATAGAATTAAAAAAAGAACCTATAAAAGAAATAAATGTAAAGTTTACAAAAGGGGAAAGGTCCATAGGGGAAACCTTTGGGGAATGGGATAGGGGGGATGAGGACGGAGATACTCCTACCAGGGAGTAAATGGTGAGGGTAGGAGGGTGAGA

>P40

GAGGATTCCTCTTCGACCCTACTACGTTACTCAAGTCTCAAACTTTTACCGTCGGTCTTCCGGAGGGGTGGGGTTGGAGGATAAAAGGTTCCAACGGACAAAGGTAAGAAAGACAACCGGGAGTCCCGAAGTCAGGAAAAGGGAGTGAGTTATGGTCTAGTTCGAGGGGAGAGGGAGGGAGAGGGGTGGGGTAGGTGAAA

>P41

GCAGGACAACTGGGGTGGCCTCAGACATCCTCAGGGAGGGAGGGAGGGATGGTAGATATTTTCTTCTTCTGGGAGTTGTATGCAAAATTAAGAGCAAGGGGGGGAAGTCTCACTTTCTGCCCTTTTTCCTTTGATGAAGGCCTTGCTGAA

>P42

TTTGGGTCTGGGTTCTAAGGGAGAGGGAAAGAGATAGACCAACGT

>P43

CAGGAGCAAGGAGTGGGGAGAAAGCAACTGGGGTTGTCAGAGAAACTGTAAGCTTGCTCACACTGAGTGAACCTATCTTTTGTTTGCTTAGCAGGCTGCATTCCATGGCACAGGACACTGCTTATGCAAAAAAAA

>P44

AAAGGAGAGAAGAGAAGAGAAGAGAAGAGAAGAGAAGAGAAGAGAAGAGAAGAGAAGAGAAGAGAAGAGAAGAGAAGAGAAGAGAAGAGAAGAGAAGAGAGAGAAGAGAAGAGTTTGGGCCTCGGGCAGTGGCTCAGCAGGTAAGAATACTCAGTGCAAGCATGAGGATCTGGGTTCAAA

>P45

GCTCGTTCTCCAGCTCTCAGAGGTGGAGATTGGGGGGCGGGGGGAATCAAGAATCACAAGCCAGCCCTGGAAAGAGGCTCTCTCAAAATCACCAGCACACTAGCAACAATAACAGTAACA

>P46

TAGAGAGAAGAACTGGGTGGGGATTAAAGGGATATTAGAATAGGGCAATTTTTATGTTTTTCTGAATTCCAAGAAAGTTATAATTATGCCTCTGGATTTTTTCATTCTCCTCCTCTCTTCTTCATCTCTCTCCCT

>P47*

GCAGGGTATTCTGGGGGCTTTTCACACCAAGTCTGTTTCCTATGTCTAAGTGTACAAGCCTATTTCTAGGCAGGGGGGTTGTGATGTGATGTGCAGATAACAGAATTCTAAGAAAGAACATTTTGTTTTTCATTTTTCATTTTGTCCTAGTTGCAAAGTCATGTG

>P48

TACAGGGAACGCCAGGGCCAAAAAAAATGGGAATGGGTGGGTAGGGAAGTGGGGGGGGGAGGGTATGGGGGACTTTGGGGATAGCATTGTAAATGTAATTGAGGAAAATATGTAATAAAA

>P49

CGTGGTGAAAGGAGATAAAGTATGTGTAGAAGGGGAGGAAGGGAGAGATGGTACTGGCTGTAACCAGCACTGACCAGGAGGAATAATGGGAGAAAGGGAAAGGGGGAAGGAAGGGGGAAATATAAAGTGGACAAGTTAGGTAAGGATTTCTATAGTGCAGCTGTAGGGTAATGCTCTGAGTTTAAATTCTAGTCC

>P50

GGTCGTAGGGTAGTCTGACGTAATAAGCCTCGGAATGCAAGAGAAATAAAAATTGTCGGTAAGCACAATCAACAGTTGGGGGTCGGACTT

>P51

GGGCGGGGTGGGGGTGGGGGCAGCTTGAATTGAGGAATGCGGGTCTGGAATCCGAGCTGCTGAGAAATCACTTTTCCCACCCAGCTCCTG

>P52*

TTCGTAAGTGTCATGGTAACATATCTCAAAGTTGTTGGTATATCATCGACAGTCCCAAACCGACAAAAGTTTGACCCTGTAAAGTTGAGAGGGAAAAGTAGGGAT

>P53

TAACAGCCTTGGGAGGACTCAAGGGGTGCTGTTGGGAGTGGGGTGGGGGAGCTAGAGGGATGGGGGCCGGCTGTTAGCAAAGGAGACTGAGTCCCAGTAGCCGGTGCTACAGGAAGCTTCTGGCGCCGAACCCACATCATAGTGAGTGAG

>P54

TTATGATTAGGGTCTTGGGTTTTGTTTTGGTCTCGAAGTAGTAGAGGTAGGGGGTCCGATCCTTTTTCACTCAGAGGACAAAACCGAAGGAAGAGAGAGAAGACGGGAGAAGGGAGGGAG

>P55

CCATACATCAGGAACCTCCCTTCCGAGACCTACGGGGAGATTATATGTGTGAATCCTTTCAACATCTTATTCCAGGGGGTGGGGGTGGGGAGCGGTCTACCACCC

>P56

CTTTCCACTACCTCTGGTGGGTGGTTGGCTAGAAGGGAGGTTAAAGCATTTAACAACCCTTAATAAGGTAGGTTCTGAAAAATCTAAGCCTACAATAAACATATCTAGTCTGAGATTTTC

>P57*

GTTGTGGTTGGTTCATTTGTAGTGTCACAGACACACACACACACACATACATACATACACACACACAACACATACTAGAGAGAGAGAGAGAGAGAGAGAGAGAGAGAGAGAGTGTGTGTGTGTGTGTGTGTGGGGTGTTGTGAGTACACC

>P58

ACCCACTAATAGACGGAATGGTCCGGTCTGACCTCGGACTCGTCTCATTTCTGGAGAAAACAAACGAAACAAAACATAAAACTCTGTCCTAGAGAGAGAGAGAGAGAGAGAGAGAGAGAG

>P59

AGACACGGGGGAAAGGGCAAATCTCTCAACTCTATGCTGGTTTCAATGAGATAGTCACCATCAATCTTGGCTTTCCATACTCCGTCCCCAGTTGGTGGCTGGGTGGGGAGAGTGAGTAGGTGTATTAGCAGGGGGTGGGCACTGAAGTTTCAAAAGACTTGCAGTTAGCACTCTCTGCTTGCCACTTGTGGAACAAGACG

>P60*

TCTGTAAAAATTACAAATATATTGAATGTTTGATTTTTCTGTGAATCAATATTTGAAGACCATTTTGTGTACGACCATGTGTATAGATCGGGGAGGGGCGGGGCG

>P61

TACCACGAGTGGACATTAAAGGAGTTATTTCTCTGATTCCTCCCTCTTAGTACTCAGGGTCCGGTCGAACCTGATATGTTCCTCTGAGACGGAGGTCGGGAAGGGGAAAGGGGATTTAACATGATGTTAATGATT

>P62

ATGGATCTGGGGGAGAAGGGAGGTGGGGGAAAGAGACTGAGGGGAAGGAGGAAGAGGAAACTGCAGCTGGACTGTATATACGAGAGGAGAATTTAACAAGAGAGA

>P63

CACTACGGTTCACACGACTTCCTCTACCAACTGTTTCACGTCACGAACCAGTTCGGCCTTCTGGGGGGGTGACGG

>P64

GATAGATCTGTTCCGACCGAAGTTTGAGTGTCTCTACACGGGTTGAGACGGAGGACTCATGACCCTGATTCACACACGGAGGGGGGTGGATGGGAGGGGATGGGAGGGTTAGGGTAGGGA

>P65

GAAGACCTATGTCTCCGAAGGACGAGACTTGGAGGTAGTCCGACCCTCGAAACGGAACGAAGAGACCCGGTTCGAGGGACGAGGGGTGGGGGTCCGTCTGGGAAGGTCCTGAGTGACCAA

>P66*

AGACAGAGACAGAGAGAGAGACAGACAGAGAGAGACAGAGACAGAGACAGAGAGAGAGAGAGATACAGAGAGAGAGAGGGGATGGAAGAGAGAAAGATGGAAGAG

>P67

GTACCAATGATATTGAGTCCGTAGAATGTAGAAGGTTTAGGGAGCAGAGGTCAAGAGTCGAGTCTCTGGAAGGGATTCCTCGGCGTAGGTAAGTGTATAGGAAAGACGGGTTTGTGTCGA

>P68

CACACAAGATGTCTCGATCCGTTCCATCACTGGTGGACCAATCTCGATCCCTCCCCGCCGTCTCCCTCCAGGGGTCGTAAGACGTGGTATCCGGTGGGTAGGGAC

>P69

TAATAATGTATCTAATATTGTTCAGAAAAACTCTCAGAGTGTGTTGGGAGTGTGAGGTCAAACAGAGGGGGCGTGGGGGGGGTGGTTGAGGGTTTTGGGGTTTAGTAGGTTCTTCGGGTC

>P70

GGGGGTGGATGGGTAGGTGAGGGAGTCTCTGGTCTTCTCTGAGAT

>P71

AGGATTTTTATTCAATTTGGGGGGTGGGGGTGGGGAAAACAACATTATTAGGTTTCCATCCCAGCATGGTTCCAGAACAAGATTTCCACAGAATCTTGATAGGAAAAGTAGAGGCTGCCT

>P72

GGGTGGGAGAGGAGGGAGAGTGGAGGGAAAAGGGAAGGATGGAGGGAGGGAGGGAGGTAGGGAGAGAGAGAGATATCAAGGGAACTGGTAATTCAAAGTTAGGATTATTTAAGTCACATTAGCCCAAGGGAAACTTAAAAATCAGAACTG

>P73

ATCACCACAAACATTAGGGGAGATGGAAGGGGCGGTGGTGGGCAG

>P74

GAAAGGAGAGGGAGAGGAGAGGGAGAGGAGAGGGAGAGGAGAGGGAGAGGAGAGGAGGAGAGGAGAGGAGAGGAG

>P75

CAGTACAGGGGAATACCAGGGCCAGGCAGCAGGAGTGGGTGGGTTGGGGAACAGGGCAGGAGGAGTGTAGAGGGGGGTTTGGGGATAGCATTTGAAATGTAAATAAAGAAAATATCTAATAAAAATTTAAAAATATATAACTTATTATCTTATACTTTATAGAAT

>P76

CGGAAATGTAGTGACCGGTCTCTCGAATGTGTGACCGATGTCTCTGTGCCTTCCTCACACCACAACCCCGGAGGGACGTCTTTGGGGATCGATTGGTGTGTGGGAGACACGACGGGACAAGACTCTACGGGGAGGGACTTCAAAAAAAGA

>P77

GGGAACGCCAGGGCCAAAAAGGGGGAGTGGGTGGATAGGAGGTTGGGGGGGTGGGTATGGGGGACCTTTGGGATAGCATTGAAAATGTAAATGAGGAAAATACCTAATTAAAAAAAATAAATAAATAAAAAAAGA

>P78

TTGGGGTGCCCAGGACTAGGTGGGGTGGGAGTGCTGCGTTCTGAT

>P79

GGGGGGTCCTAATGCGGCCCTCCGTAGTGCTGACGCCGTACTTGAGGGCAGCTTATTGTAGAGTCTTACTAGAGACTCACTAGGTACCGG

>P80

AACTACAAATAAATAACAAGACATCGAGACGGACCGGTCCTTGAGTAATACCTCAGGTCCCCTTGGGTACCCTTGGAGTCGGGGGAGAGG

>P81

AAGGCATACAAAATGTGGACTGGGGGGGGGCATTTACTTTCCTTGTTTGGGGCACACTTTAAATGTAAATTACTTTCTTGGGCAGTAGCCACATCCACATCCATGAAGTCAATAGTTTAT

>P82

GGATTCTTAAATGATTGTCTTTGGTTCTGGTGAGTGGTAGTAGTCTTGTGTTGTGAGGGTGGGTTGGGTCATGGCACGTGGAGTTGTATT

>P83

GTGGAGCTGGTAGCAGGGGCTGTGGGGTAAGGACATAATCTGTGGGTGACAGCTGCACAGAGGAGAGAGTCTGGGTCCCCTTCCTTTCAGGAATAACAAAGGCATCTGGAGTAGGGGACAGGTGGGGGACTCAAGTTCCTCTTTACCCGGGTCTGAGTTCCTGCTTTTTACTCTGATGCCCTAATCAGGAGGTACGGAAA

>P84

AAGGGAGAGGGAGGGTGCAGAAGATAAAGGGAGCAGATTGTGCAGAGCCTTGTGCACCACGGGGAGGCCTGTGGGCAGCTGGGAGCCAGA

>P85

CTTGTGTTTTAAGAAAAGGGGTCTAAGGTGGTCATGGAGGTGGGATGGGAAAGATATTAGATTTATGTTTATTAAAAAGGCACTCTATTAGACAACTTTTTTGAGCTTATATCTGGTTGGTGGAGATTTAAGTTTCATATTGGGTTCATC

>P86

AGCAACCATTAGCTACCCATGAATCCTCAGAGGTGGGTGGGGCCTCATGAGCACCTTCTCCCTCTGTGACAGAAGATTGACACGTCTGATCTCGTGTAGATCTTGAGGTCTTAGGCAGGCCATCAAAGATGCCAG

>P87

CGAAACAAATCAAAACAACAACAAAAAATTAAATAAAAAATAACCTATATAAGAAATAAATGTGAAATTTACAAGAGGGTAAAGGACCAAAGGGGATGTTTTTTTGTGAGAGGATGATGGGGGAGAGTGGAGGGGGACGAGTGGATGGGTGGGTGGGTGAGGACG

>P88

ATTTCACTTACGATTGAAAAGTAAAAAAATAAAAAACAAAAAAATAATCAATAAAAGAAATAAATGTAAAGTTTTAAGGGTAAGGAGAGGGGGTAGTGGGCCGAGGGGTTAGATGGGTGA

>P89

TCCCCCATATACTTCCACTACCACTGGTGGGTGGTGGGCAGGAGGGAAACTTGAACTCTTATTAAAGTAGGTTGTGAAAATATATGCCTGCAGAAGGGAGGGTGGGTGTTTCAGCTGATGGCTCGAGGGAGCTGTCCATCGTGACAGGGATGGCCTGGAGCAGGAGTGTGAAGTGGCTGCTCCTGTTGCTACTAC

>P90*

GAGTCTAATATCTGTACATCTCTTTACGAAAAATGGAGTGTAAGTGTAAGTACTAAAGTTGTCGAAACGTTGTGG

>P91

AAACTTACTGAGACGGAAACATTTTCCCGAAACTCATACTGTCCCACAGAAAAAAGAAAAATCTCCTTCTCTTAGTTTAGGTACTGAGCGGTGGGGGGAGGGGGGGTACTCGGTACACCT

>P92

CTGCTGCTTCACAAAGATCTGGGGAGCAGAGGGGAGGAGTGAGGGGTGGAGACATCAGAGCCCTCAAGTTTAGGCTGAACACATGCTGCTGCTCACCTGGAAAATGGCATGAGAGCGGGAGGATGTGGCGTTGGCATCCGTGGGGTGCTGTGTGCGGCTACAGTT

>P93

TGGAAAAATCATTTTTAACCTGGTAAATAAGAGAGTACAAGTTTCAAGAGAGTTTAAAAAAAAGACTGAAGTTTTTCTAGTGGGGGGGGGGGAGGAGTATCCTGTGGAGATGAAGGAGATCGGAGGTGAAAGGTA

>P94

TAATGTGGCTCCCTCTTATAAGTTTGGGGGTGGGGACAGCTATTCACGTAGCATTTCACAGAGATAAACTAAGCTATCCTTGCACAGGAC

>P95

AACGACGAAGTATTAACATTAAAACGATGACAATACTTAGTATTACATTTATAGACTATACTCTCTACTCCATATTGGGGACACTTTCTCAGTAAGTGGGGGGGGGGGGTTTTCCCCGAAGCTGGGTGTCCGACTCTTAGTGACCAGATC

>P96

CTGAAGACAAGAAGGAGAAGGAAGGGGAGGAAGAAGAAGAAGAGGAGGCAGAAGAAGAGGAGGAGAAGGAAAAGGAGGAGGAGAAAAAGAAACACTTTCTTTTTCTCTGTTGGATGTTTTGTCACAACATCAAGTAATAATTAATAAGGGTGCTGTGATAAATCT

>P97

AGAAGAGAAGAGAAGAGAAGAGAAGAGAAGAGAAGAGAAGAGAAGAGAAGAGAAGAGAAGAGAAGAGAAGAGAAGAGAAGAGAAGGAATTAGCTATCCTGATGGCAAAAATCAAACTTGCCCTCAAGGAACTTGACACCCCTAATCAGCAGGAAGCACTGTAAAGATATTGTGGGCCCCT

>P98

GGAGGAGGAAGAAGAGGGAGGGGGAGGAGAAAGAGGAGGAGGAGTAGGGGGAGGAGGAGGAGGGAGTGGAGGGGAAGGAGGAGGACAGTATCCCTGTGAGTGGTTTCTGCAGCCCACACTCCATTTAGCTGCACTACCTGGAGGCTGCTGGGCAACTGGCCTAAT

>P99

AAAGGAGATGCAAATTCAGACTGGGGGGTGGAGGGAGGTGGAACCAGAGATTGCTATTCCCTAGTAACAATAATGTTAATGACAGCAACTGATCAACATTATGGAGTGTTCACCAAATGA

>P100

GACAAAGTCCCTGAGTCCACGGTGGGTGTGGGGGAGGGGTGAGAGGCTCACAGCCCAGTCATTATGGGAATATATGAATAGAGCAGGTGTGATCAGTGCTCAAAAGCAATAGTTTCCACA

>P101*

AACAGAAAACACATTTTAGGAATCGAAACGTTGTACCAAATTTTAAATCCTACTCGAGGAGTTCACAGTCTCCCAATGTATACACTTTAGGGGTGGGATATTTCACTAAGTCATTGTCAGTATGTTAGACATTTC

>P102

GCCATGAGGAGCAAGCCAGTGTGGGGCTGTGGGGTGTAGGGGAGAGGTGGGAGGGAGAGAGCCCACATCTGACCAGAACGACTAGGCTCTGGGCAGGCAGACGCAGGAGGGCTGCCGGAC

>P103

CAGCTTGCTAGGGTGCCTTCATTTCTAGTGAGAGTGAATGGGAGGGAGGGAAGTAGAAACTAGCCAATCAAAGGTATAATGACTCTCCCTGTTCTAGGTTCTTGTCTCTTTGGAACACGTAGTCCTCCTTTGGTTGTCCTTTGTGAGAGT

>P104

ACCTGGGTGGCTCAAATTAATGTCACCCCTCCCTATAAACAATCTTGTACCTTTTATCTTATCTGGTGACTTCTTTACTGAGGGAGAGAGGGTGTTGGTGGGTAGGGGTTACCTCAGGTG

>P105

CCTTTCCCCCCTACACCCGGGTAACAGAAGACCCTCACACGTCTGTGCTACAATAGTAACACAAATGTCTCCACCAACCTAAGGGGCACGAGACGTGTGGGGTGTTTCTACTGACAGGGT

>P106

AAGGGAAGGGAAGGGAAGGGAAGGGAAGGGAAGGGGAGGGAAGGAAAGGGAAGGGAAAAG

>P107

TCTCATGAAGAGTCTTACTGTTTGATCAGAAGAAAATTAAAAAATTTTAAATTCAATTTTATATTAATGTATAAAGGGGAAGGGAAGAAGGTTGGGAAGGACACGAAGGGAAGAGGAAGA

>P108

GTCCGTAGAAGAGACCGGTTGACTGGGAAAGACGAGGAGAGGGACCTTCACGGGGGGGGG

>P109

ACACACACACACACACACACACACACACACAGAGAGAGAGAGAGAGAGAGAGAGAGAGAGAAGATGACCAAGGTGACTTACAGAAGGAAGGATGTGGTGATTTCAATAGGAATGGCCCCCATAGACCCATGTGAGTGAATACTTAGCCCG

>P110

AATCAACGGGGAGGACGTCAGGGTCGGACGAAGAAGGAGACTCAGACCGAGAAATCATAGAGTCCCGACTGAAGGGAGTGGTGGGGGGGGGAGGGGTCCAAGATAGAGAGGGGTCTTCAGGGTAGAAGATTGAAA

>P111

TTTGGGTTTTTTTTTGGGGGGGGGGAGAGGTTGTTACAGGGCCTC

>P112*

TGGGTCTCTCGGAAGTCAAGGGAGACTGTAACAGACAGTAGAACCCCTGTAACATAAAATCCTCATTGTGGGATCTTGAAGGAATGAGTT

>P113*

ATAACGGTCAGAGAACAGCATTCAGGAGTTGATTTTCTCCCATAG

>P114

CGCCGCGAGGGTGGGGGGCCGAGGCCGGGGGGCCGCGGGCCGGCG

>P115

AGTTCGAGGCCAGCCTGGTCTACAGAGTGAGTTCCAGGACAGCCAGGGCTACACAGAGAAACCCTGTCTCGAAAAACCAAAAAGGAAAAA

>P116*

ACTACAGTAAAATGTCTCATGGGGGGGGGGTTCATTGCTAGACTCCTGATGTCTTCATACAAGCATTTGTATGTATAGATACATTTGTTT

>P117

GTGTGTGTGTGTGTGTGTGTGTGTGTGTGTGTGTGTGTGTGTGTGTGTGTGTTTGACTCATCTTCAATAGTGTCAGAGTATACGGGACGGGGCAGGGATGAAGAAACACAGACAATAAGGATTGCAGAAGGTCTA

>P118

AGGAAGGTAAGTGGGTAAGAGGGTAGTTAAGTGGTTGAGAGAATAGGTAGGTAGATTGGTGGGTGGGTGGGTGAGTAGATAAGTAGGTAGGTGGATAGGTAGGTAGGTAGGTAGGTAGGTAGGTAGGTAGGTAGGTAGGTAGGTAGGTAGATAGGTAGGTAGGTAGGTAGGTAGGTAGGTAGGTAGGTAGATAGGTAGGT

>P119

CGAGTAGATTTGACTTTCGGGGTTGGGTTGGAGACTCGGTACGGGATTGTCCCATTCCCG

>P120*

CAATGAGATCCTATG

>P121

GAAGAGACTTGATGTGCCAGGGTGGGGGGATACCCTGGGGATATTCACTGTCTCAGATGAAAAGGAAAGGGGGAGTAGGGGGAAGGATTGTGAGAGGGGGGTCACTGGGAGTGGGAGTAGTGATCAGGATGTAAAGTGAATATAAATGAATGATTGAATGAATCTATCAATCAATAAATAGAAGTAGGAACATCGGGAAC

>P122

GAACAAGGGAGGAAGGGGATAAATCCGGATTTACACGGAGTAAGCCTAAAATACCAGGGCATTCATATGTGGGAGCTATGTTGCCACTGGGTACCCGGAGCCCAC

>P123

GGAAGGAGAGGAGAGGAGAGGAGAGGAGAGGAGAGGAGAGGAGAGGAGAGGAGAGGAGAG

>P124

CCCTCTGACGTTAACCCTATACTTCATTCACCTATTTATTTATGTAGTCGTTTTTAATTAATCAATTAATTAATTAGTTAATTCTTTTTTGAAGTGAGGGGTCGGGTAGGGTCTTTAATCCCACGGTCGTTCGAA

>P125

AGAGAGACTCTGACCAAGACATGGGCAAGACAGAAATAACCTAAAAAGGAAACTGTAAAACTTCATCCCATGATCAAACTCTTTGCCTAA

>P126

TCAGATACTTTGTGATTAGAATTGGTTCGATTCGTGTCAATTTACAAGAAAAAAAAGTAATTAATTAAATACATAAGTGAAATGTGGGACTAGTGTCGGGGGAGGGAGACGAGAGGAGGGTCAGGGTAGGAAGGTGGGGGAAGAGAGTAAAGGGGAGGGGAAGAAGAATCTCTTGGGTGT

>P127

CTGGGGGGTTGGGGGGGTGGGTGTTGCTTGTTGGTTTGAGACATAGACAACCAACTCCTGCTTCTTCAGCCAACCAACAGGTACTGAAGATCAAAAGACCAGGAC

>P128

GAACGAGACATCTCGTCCGACCGGAGTTTGGGTCTCTAGACGGACGGAGACGGATGGTTTACGACCCTAATTTCCACACGTGGTGGGGGTGGGTCGATCCCGAAGGTCAACTGCGGGACG

>P129

TATGTAAGAGGGTTGTCACTATGGGTAGGGGTGGGGCAAAGGAATGAATGTGGACATCTGGCATCTAAGTTCTGACACGCTTCTTTTGTCTATCAAATGGTTTAA

>P130

GGAGGGAACACGTCTTCGGTCCTGTCCAGTGGACTCTGGGCTGAGGGTCGAGCCTGTCAGACCTCACTGGCTTCGCCCGGAAAGGAGCCGATAGGCAGTTTGGGACGGGAGTGGTGGGCCGGGAACGACGGGAGGGAGGAGCCAATGGTACGACCGGCCGCCGGC

>P131

AACCAGCCTAGGGGAGCGGGGGAAGGAGAAGGGGGGTCGGGGACAAGCGAGTTGTGAGACTGGATTCATCTAGACAGCTTTCACCACTGAACCTGTCTCTGGGTCCAGCAAACCCAGCCT

>P132

AGTGTACGAAACAAGTCAAGATCCCAATCGTCAAAATAGACGATCAACCGAAGGAAAGATACATTTAGTACAACAAGTACTACGGTTAGGGGTTGGGTCGGGTCC

>P133

AATAATGTCAACGAAACCGGAATAACTTAATCACGAGTCTTGTAAAAGTATTATAAAATATGTACTACTTTGGGATCCGTAACTATAACTACCAAAGGGACGGGAGATGTGGAGCAAGGGGTGGGTCGAAACACTCGGGTCTTATATATC

>P134*

CTTATGACATGTTCTATTTGTCTCGGACAGTCAGCTGTTTCTGTGAGATAACTGGGGTTA

>P135

GAATATTAAGAAATTTAAATAAGAAACTTTTAAAGTATATCCATACACAACATAAAACTAGGACAGACGAAAATGATGGAGGGAGATTGAAGGAGACCCTGGATC

>P136*

AGATGTATATTTCAACTGTATTACCACCGCGGTTAGAATTTCGAA

>P137

AGTACAAACCGTATTTTAGAAAAAAAAATTAATCTATTTAAGAAATAGATGTAAAGTTTACAATACGGGAAAGAACCAAGGGGGAGACTTTTGGGGGTAGGGTGGAGGAGAGGGGGACGAGTGTTTGCATGGGTGAGGGCGAAGGGACAG

>P138

ACAAAACAACTTATTGGTCTAAACAGACAGGCGGAGGGTGGGGGACCAGAAGAACAGTGT

>P139

GGAAGTACCTGTGGGCTGGCAGAGAACAGGGACAGGGGACACAGTGTGGCTGGAGCAGTGTCATTTGCCTGTGCATATGTTGGATGGGATGGAGCTAGGCAGCAATGGTGAGTTTCAAGTTCTGTGTGAGCTGAGGGAGAGGCAGGGGCCTGGAGAGAAGGCTCCCTGCCATAAGGAGAGATGCTGCCCTCACCTCCACA

>P140

TATGGGTGGGGGGGGATAGGGGATGGGTGGGTGAGGGGGAAAAAT

>P141

GAGGCCGGTAAGGCCCTGGGAGGCTGGGCAGTGTGGTGAATGGGAGAGAGGAGGCCAAGGGGTGCAGAAGTTCGGATAAGTCCTGGCGCACACTCCAGAGTGGAGAAAAACAAAACAGCCCGGTCTGCGGTGACC

>P142

TCAGCGGTATCTAAGAATTTTCTTACTTGCCTTGGTGTAGTTAGGGTGGGGGTCTTTTTTCTGGCTTTCTTTAGCCAGTGCTGCCCGTCTCGAAGGGTGTATCTCAAATAAGTAATTTCAGTCTGACACAGCTGA

>P143*

CTTGGGGGACAAACTGGAAGACTCTCAAGTTAATGTCCATGCGTC

>P144

TCCCCATATTTATCCTCTGGCTAACACACGGTGTGTGAGATAAGTCTCGAAAGCACGTAGAAAGTAAATTATTCTTGTAGGTTTCTACGTGACCGGGGAGGGGGTGGTGTCGACCGGAGGCTCAGTGTCCTAAGTGTCAGACGTTCCGTG

>P145

GGCTGGGGTCCTGGGCTGAAGAATGCGGGGGTTGGGGAGGGAGCAGCTGGGTGAATGGCTCTGTTGATAAAGTGTCTGCTGCACAAATGCGAGGGCTTGAGTTCGGATCCTCAGAAGACACATAAAAAGCCAGGG

>P146

GTGGGGGGAGGGAGATGGAGAGAGGGAGTGGAGGGAGGGAGGAAGAAAGGGAGGGAGGGAGGGAGAGAGAGAGAGAGAGAGAGAGAGAGAGAGAGAGAGAGAATGATGGGAGTGTTTTGAAACCTCAAAGCCCTC

>P147

GTTTCGGTCTAACACGTCACAGCCGACTGGATTCTCCAACTAACGTCCGGGTCCCGAGTGGCTGGTGGGGGGGGGTGTGTGTGTGTGTGT

>P148

CATAGGGAGGTGATGAGATGGGGAAGAGCTGGGAGGAGTTGGGGGAGGGGAAAACATGGTCAGAATGTATTGTGTGAAATTTTCTTCAATAAAAACAAACAAAAATAGTATGGATTATTGACAATATATTTTTCT

>P149*

TAAAAATTAAAGTTTTTATTGGGGGCGGGG

>P150

GGAGGTAGAGATGGAGAGGGAGACAGAAGGAGCAGGAAGGAGGTTAAAGTGAGGGGGAGGGAGTGGCGGGAAGGCAATAGAGGAGCCAATAGTGGGGAGAAGAGTGGGGATACAGCCCTGTCATCACATTGAGGTTCAGAACCCAAACAGGCCTGCCAGACAGTT

>P151

TTAAAGGGGGGGGGATCGAGTGAAGAACTCTTGGAGGGGGGACCAGGAAAGGAGGCAACATTTGGAATGTAAATAAATAAAACAATTAAA

>P152

CATCTAACTAATAATGAATAATTACGAATAGTTACGAATAGTGGATCGGATTACCGAAGAAGGAAGGAAGGAAGGGAGGGAGGAGGAGGAGGAGGAGGAGGAGGAAGGAAGGAAGGAAGGAAGGAAGGAAGGAAGGAAGGAAGGAAGGAGGGAAGGAAGGAAGGAGGGAGGGAGGGAAAGAGGGAAGGAAGGAGGGAGGG

>P153

CCCAGTACAGGGGAATGCCAGGGCCATAAAGGGGTAGTGGGTGGGTAGGGGAGTGGGGGT

>P154

TATAAATTACCAATACCTCGTAGAAAACCGATAGATCCAGGGTGAACCTTATCGATGGTGGGTGTACCTCTTATAACCAGAAAAGGATAGATATTAAAGGATAATTCGTGAACGGGTGGGTCGAGTACACAAACT

>P155

TGCCAGAGGTAACGCTGATCCAGTGGCCGGGACCCGGTACGGGAGTGGGTGGGTGACGAG

>P156*

TACTAAAAAGTAATAACTGTTGATGAGGGCACTGTGGGTAAATGTTTCAAGGAATAGTTAAACAAATCTATAACA

>P157

CAGGGGCAGGCTTTGAAAGTAAGGGAGGGAGAGGGGAAAAGAGAGGGGAGAGGAGAGAGACAGAGACAGAGAGACACAGACACAGAGTCAATGTGAGTCAGAGACAGACAGAGAAACAGACACAGAGTGTGAGTC

>P158

TACATGTACACACGTAAGTACACACACACACACACACACACACACACACGTGAACGTGCGTTCGTGAACACTGGG

>P159

AGCTGATAGTCATGGGCACCAGACCCTGGACTCCCGAGGGGGAGACCATGACTCACACTGAACTCTGTACAAGAAGCAGTTCTCCCTCTACTGAGGGATCCTATCCACGTTCTGTGGTAG

>P160

TAGAGAGAGGACTATGTCAGTAGGTGAGATCAAGAACACGGATCTGAGATCTACCAAAGTAGACGGTAGAGACTGAGCTGGTCGAGGTCGGGGAGAGGACGAGGGTGTGTGGATATACCCACCAGTTTGAGTCAG

>P161

AGGGGAATGCCAGGGCAGGATGACAGAAGTGGGTGGGTGGATGGGGGAGCATCCTCATAGAGGTAGAGGGGGGAAGGATGGGATGGGGGCTTCTGAAGGGGAGACCTGGAAAGGGAAAAACATTTGAAATGTAAATTTAAAAAAATCCAATAAAATTAAAAAAAAACCAAACAGTGGTTCAAAAGAACTCAAAAC

>P162

CCCGTCGGGTTGATGCGTCGCTGGCGTCGTCGTATCAGGGACCGACTACACGTTCAGGATGAAGGAAGGCCACGCAGGACCTCGGGGCGGGGTGGGGAACAGGCCGGGGCGGGGAATTCCCTAGGCGATCATAAA

>P163

CAATTACGTGATTATCCTTTTTCCTTTGAGTCTTCCATAACAAATAACTCGTGATAACGACACCAGGGAAGAGGGGGCGGGGGGATCTCTTTCGTGGTTAAAGGTACTCCATGTTCCGTA

>P164

AGATTTTAATTTTGTTTTTGTTTTGTTTATGGGGGGGGGGGTTTT

>P165

AGACTGTTTCCTCTCTCTCTGGCTGTCTTCCTTTTAAAGAAGAAATTCGAAGAGTGTGGACTCCTTTTGGGAACACTTTCTCTCCTGTGACCTTGGGTGGTGCCGGGGACAGCAACAGTGGGGGTGGGGGTGGGG

>P166

GCAGGGTAGAGGGCTGCACACGTTCCCTGGCCTGGAAGGGGGGGGGGTGGAGGAAGCCCAGAGTGAGGCTTAGACTCATTAGTGATGGCAGGCAGCTTAATCCTTCATTTCCATGCTCAGCTCCCCGGGTTCTCA

>P167

GACGAGAGAGAGAGAACGAGAGTGAGGTAGGAGATCAGGGAAAGGGAGAGAGGGATAAGGGGGGGAGAAGTGCACGAGTACCGGTCGGAGATGAGAAGATGAGAGAGAAAGAGAGACGGAAAGAGACAGAAATGATGGGAGAGTTGAGGGGAGGGGTACGGGACTTATTTGAGATAAGATATGATATAGTATGACACCGA

>P168

AAAGGGTGGGGGAGTTTTTGAACCCCTGGGGTAACCGCGTCCAGGTCAATTGTCCCATTTGTCCAGTGTCAGGAT

>P169*

TTTTGTTAATGTTGTTTGGATGTTTGCTTTTTGGGGTTGGTTTTG

>P170*

GAAGAAAGAGAGAGAGAGAGAGAGAGAGAGAGAGAGAGAGAGAGAGAGAGAGAGAAAGAAAGAAAGAAAGGTAGGTAGGTAAAAGTGAACCAAAATCAAAGAAGAAAGAAAGAGAGAGAGAGAGAGAGAGAGAGAGAGAGAGAGAGAGAGAGAGAAAGAAAGAAAGAAAGAAAGAAAGAAAGAAAGAAAGAAAGAAAGAA

>P171

AAGGGAGTGGAGCATTGGGAGGTGGAGATCATATGTAGTGAGAACAGGAGAGAGAAAGGGGATATGGACAAGGGGTTGGGGGGCAATGTTTAGGACCCATCAAAGACCTGGGATGAGAGGAGGCCTCAAAGGATCTATGGGGGGGCAATTCTAGCGGTGGGGATATGGATTCTGAAGTGGGCACTACCTGTAGACAGGTA

>P172

CACACGTGGGGGTGGGTGCACCATGTAGGAGTGGGGGTTCAGAAGAGGGATGTTCATCCCCACATACCCTCTTCTTTCCTTCCTTCCACTCCAAACATATCTCTCTTTAAACTATATTAAAGTATACTCTGCTTC

>P173

TGGAAGGGAGGAACAGAAGGGGAGTGGTATTTGGGAGGGAAGGGGTTTTGGAGGGTGAAGGGTCCCGAGTCCACT

>P174

GGGGCAATAATAATCTTACTCCTGCCATCTCCTTGGACATGCCTCAACCCCACTCTGCACCAGGCCTGCTCCCTA

>P175

AGACACTATACGATACAGTCTTTGTCCCGACTGTGCGTCTCCTACTACCAAGGACTAAGGACGACCCTCTCCCCGGTATCATCGGGAAGAGGTAGTCGGAGTGTGAATGGGAGACGGGGAGGGTTTTGGTGCACATGACGAGGTCACTAT

>P176

AAGGTGGGGGTGAGGACATCCCCCTGCAGACAGGGCGGGGGATGGGGAGGAGATATGGGATCCGGAACAGTTAGAGGGTGGATGGGGGTGGGGAATAAAATCTGGAGTGTAAATAAATAAATAAATAAAAATTAAAATCAAGTTGTTATCAAAATCCACATCTAG

>P177

GAGGGATGGGGTATAGGTCCTTACGGGGCCATGTAGGATGTTGGTAGCAGCAAAAGCCACTGGAAGGGCAGGCAGAGAGAACATCTGTGGGGACAAAGGCTAAGAAAAGAATTTCTTTCTTTTACTGTAGATAAT

>P178

TTCGTTATGACTGTATTTTCCGTGAGATCCGAAGGTCTTTCTTCCTGGGTTCGGTGGCCAAGGACGACCGGAGGACCTAGGGTGGGGGTGGGGGGTGGGGACCCC

>P179

TTGTGACCCGTTCTCTGTCCGGGTTCTTCTTCTTCTTTCCGTTGCCACCGAGAGACTTCAATCTTTTCTCTGATACAAAGTATGAGGGGGTGGGTGGTCAAGTTTCGTTCAATCTCTCAAGGATCCGTGACGTCT

>P180

GGGTCCACCCTGGGCCACCCACCATTAAAGGTGGATTTACCCTGTCTTCCGTAAGCTAGTATAGACGACCTGGGGACCGAGGCAAGTTTCAATGGCGGGGGTGTCGGGGGTGTCAGGGGTGTCCTGTCCGCACAC

>P181

CCGGTGACGGACGAAAGAACTTCCGTCGTGGGAACAGAGGGAGGTCAAGTTACACCCCCCAGGTCCATCGTTCGGGTAGACCCTTCCTTTCCGGGGGGCTTGTAGGTGGAACGACGTCCGACGTCCACACCTCCAGAGTGGTTTGAGAAATTCTGAGAGAGGGTGGGGGTTTGAGGGGTC

>P182

TTTTGGATTGTTTGTTCTATCTACTGACGTCTCCTTCCTGTGTCTTCCTATTGGAGTTTGGAGGTGTGGGTGTGCACATACGCACGCTTGGGTGGGGGGGTGTTT

>P183

ACTTAAGGGAGGAGGGGGATTATTCCATTTACAGTTTGCAGGCAAGTTTAGCATGGTGTAGGTTTGGCTGCTATGATGGCTCTTATTCTGGTAGTACAGTGAGGC

>P184

TGACTGAGTTTTATTTTCGTTTGTTCAGGTGGCGTCTCATGTAAGAACTTCCCTAGGTCTTACCAGGAGTAGTAACTTCATGGAGTCGAGTGGGTATGGGATTGGGTAATCGGGAGTCTAAAGAGTCAATTCAGA

>P185

TGAGGTAGGGGTGGGTATGTGGTGGGTTGGGGAGAGGATGGGTAGGGGATGAGAGGTGGAAGTGGAGAGTTAGGGTATAGGGAATGGCTAGGGGTAGGAAACACCCTCTTAGACACAAAGGAGAGGTGAGATGGGGTTAACAACTCTGGGGGTTAGGGAGCAACATTTTGAATGTAAATTAAAAAAAAATACAAGACTAC

>P186*

ACCTATGCACATAAATGTGTGTGTGTGTGAGAGAGAGAGAGAGAGAGAGAGAGAGAGAGAGAGAAAGAGACAGAGAGAGAATGAATAATATGAATCTAGCAGGCA

>P187

CCTCCCAAGATTGATCCCTCTCCATTCCTTTCATTATACTTCTTTCTTACTTTTTATTGT

>P188*

TGATATACACAGCTTAAAATTCACAATCTGTATGTTCAGACCCAAGGCTACAGGCACTCA

>P189

GTGAGGGCGGGCGGTGGCGAGGCCGGCGGCTGCGGGCGGAGTGCGGCCGGCGCGCGGGGCCGTAGGCACCGTGCCCGGCGCGCGCCCCCCTCGGCGGGCCTGGCGGGGATCCCGGCGCAGCTTTGTTCCCCGTGCGTGGCCCGCGTCGCCTGTCCTCCCAGCCGCTGCCCTGGGCGCCGG

>P190

ACAGGAACTCAAGCAGGGCAGGAAGCTGGGGAAGGAGCTGATGCAGAGGCCGTGGAGGCTTTGCTGCTCTACTGGCTTGCTCCCCATGGCTTGCTCTGCCTGTTCTATATAACCCAGGAC

>P191

GTACCTTTAGATGAGGGGGGGATAAAGGGAGTGAGGGGAAGAGAGTCAGAGAGAGAGAGAGAGAGAGAGAGCACGAGAGCGAGCGAGCACGAGAGCTGTGCCATGCATATACTGGCAGCACTGAGTGGATTCTGTAGGTTAAAGAAAAGTACCTACAGTTGAGAGGGTAAAGTGGAGTATGGGTGGGGCTGGAGGAAAAG

>P192*

AGACACAAACAGGTGAAATCGAAAGAAGAGATTGTGACACCCGAAACGTACGAGACAAGAGAGAAAGAGGGGGGG

>P193*

GAGCTTTTGTTAATGTGCCTCCCTTATTAC

>P194

CTGGACCCTCAAGGTAGTCCCAACGAATGTCCTCCTACCGACTCCCCATGAATACCTTTGTCTTTACTGAGTCAGTGGACGTAGTGGTCTCGGGGGGGGGAGGGGGGCGTCGTGGTCGAGTTCCGTGTGATTCGG

>P195

AGGTGTTGGACCCCCTGGAAGGTGTTGGACCCCCTGGAAGAGGTGTTGGACTCTCTGTAAGGTGTTGGACCCTCTGTAAGGTGTTGGACCCCCTGGAAGGTGCTGGACTCTCTGTAAGGCCCTGGACCCCCTCTA

>P196*

AGAGAGAGAGAGAGAGAGAGAGAGAGAGAGAGAGAGAAAGAGAGAGAAAGAAAGAAAGAAAGAAAGAAAGAAAGAAAGAAAGAAAGAAAGAAAGAAAGAAAATCAAAGTGGACATTATCC

>P197

GAATCAAGGGCTGGGAGGCGAAAGTGGGTGGGTCAGTGGAGGAACACCCTCATAGAAGTAGGGGCTGGGTTTCCAGGAGGGGGGCAAACCAGGAAAGGGGATAACATTTGAAGTGTAAATAAAGAAAATATCATACACACATACATATATATGTGTATGTATATA

>P198

GTAGTCGTCATTCTCGTGTCTGATGTCTAAGACAGAAGGTACCCCCTCACGACCCGTGTAGGTTTCCCCTTTCGTTTGTCTGAAAGACGGGGTGGGTGTCGTAAA

>P199

AACTGCTGTGTGTGTGTGTGGGGGGGGGGGGGCGCCTGGCTGAGGTGACATGTCCTGTCCTCAGTGCTCTACTTGGCAGCTTAGATGTAGGAGCTCAGCTGATGTGTCAGCTCTACATTG

>P200

GTAAAGGTTTATGTTGGACAACTCAAGTATACTACTACGAACATACATACGAAAGTCCTACTGACGAAGTGGGGCTGTTGGTTAATCGTAGAGGGAGAAGGGATACCTCCTGGTGGTGTGGGTGAGAGTAGAATG

>P201

GTGGGGGTAAGTAAGAACCCACGGAAGGAACAGGGTCCTGTACGGAATAGGACCTCTACGGGGGATGAATGAATGGGGACAGTCAACATCTAAAGGTAAGTAAAAGTACAGGAAGATCGGTAGAGAGGGCAGGAAGGGGTGTGGACTAGAACTTGGAGGGTAAGGGGATGGGTAAGACGGAGGGAGAGTCAAGGGAGGGA

>P202

AGAAAAAAAAATTAAATAAAAAATAATCTAAAAAAAATATATGTAAAGTTTACGATAGGGCTTTCAAGGGATATGGGAGGGGGACAGGACGAGGGGATGGGTGGGTGAGGACGAAGGACC

>P203

AAGTTCGTAAAAGACAAAAACAAAAGAACCATCGGTAAGAAAAAGAAAAAATTTTAAAAGAAGAAGAAAAAAATATAAGATAAAACCGACATCACAGGGGAGGGAGGTAAAGATAAAGGGGCTTGGAGAGATGGGGATGGTACGACGGAGGGGAGTCTCTTCTTG

>P204

AACTGAACTAGTCTCGGACTACTCAGTGAAGTACCTTAATAAAAGGACGGATAGCGCAAAAGAAAGGGAGGGAAAGAGGAAGAGGAAAAAAGAAGAAGAAGAAGAAGAAGAAGAAGAAGAAGAAGAAGAAGAAGAAGAAGAAGAAGAAGAGAGACAGAGAGAGAGCAAGAAAGGAAAGAAAGAAAGAAAGAAAGA

>P205

GGGGTAGGAGTCTCCTGGGAGCTGGCACGTCCCTCATGGTGGAGCCTCACCCTCCAATCAGAACTGAAGTCCCCC

>P206

AGACCAAGGAGTGGGAGGTAAGAGGGAGGGGGAAAGGAGGGTCAT

>P207

GGAGGTGAGGGGATGGGTGGGTAAGGGTAAAAAAACCGGGACCGCTAGGGGACATGACCC

>P208

ATTGCAGAGGTGACACTAGAGAAGCAGTCTATGGGTGGGACAGGGAGGCATTTGCTACAGCTTCCTGAGGACCCTGGGTTACTCTGAGGGCTCCAGGAAGCTTGCAGGGAACGAAGGAACAGCCACACGGTCAGTCACACTTGTGTTTGC

>P209

GTTGGAGGATGGAGGACTCCAACGGACAAAGTTAAGAAAGACCACCGGGAGTCTCGAAGTCAGGGAAGTGGGGTGGGTTACGGTCTAGTACAAGGGAAGAAGAAGAGGGTAGGGGGAAGGGTAGGTTCGGGGAGGGAGAGGGTTAACATG

>P210

AAATGGAATAAGGTATCATACAGAGAGGGGGGAGGGAGGGAGAGAGAGAGAGAGAAAGAGAGAGAGAAAGAGAGATGAGATGAGATATATGCATACCTCATCTAAAAAGCTTTTGCACAACAAGCATCTAACAGA

>P211

AGGAGATATGAATGTCTGTGTGGGTGGGTGTGTAGAGGCCAAGAATAGATGTCTGATATCTTCCTCTATCTCTGTCTACCTTTTTCTTTTTTGACTTTTTTGGCTAGAGTGGTAGCCACA

>P212

TCCCGCCTCTGGGTCCCTCCCAAGGGCTGGGATTAAAGGTGTGGGCCACAGTTCCTGGCAATTTCTCTAATTTTTTTTAAAAAACATTTTTCTTTCAAACCCTGTGTCCAGGGCTGATTT

>P213*

TAATAATACACCCCAATAAGTCAATACGTCACCATTGCAGTGTTTTGAGTCTTAGATGTCTGTAACGAAACTACCTGTCCAAATCATAAAGAGAGAGAGAGAGAGAGAGAGAGAGAGAGAGAGAGAGAGAGAGAG

>P214

TTTAGAGATCAGGTGGGGTGGGGGGTGAGGACATCCACGTGGAGACAGGGGGCTGGGGAGTAGGTGTGGGATGTGGAGCAGTCAGAGGGTGGATGGGGGCAGGGAATAAAATATGGAGTGTAAAAAAATAAATAAATTTAAAGGGGGTCATCTGGCTCCCGCTCTCACAGCCATTGCAGT

>P215

ACTCTTGAGAGACTGGAAGCCACAGGGAAAGGGGAGGCCTGATGGGGTGGGGGCCACCCTCTCAGAAGAATGGGATGAGGAACTGTGGGATATAGGATAGAATGGGGCAATGACTGGAATATAAAATAAAATAATTTAAATTAACAATTG

>P216

TTTAAGTTAGTACCAAAAAACTAACAAACAAACAAACAAACAAACAAAATAATCTATAAATAAAGTAAATGTAAAGGTTCCGGTAGGGTTTTCAGGGGGTGTGCGAGGAGGTGGGTGAGGGGGTTGGTAGGTGAGGGTGTAGAACTGGGA

>P217

CGCTAACTATCCTCTACTACTTTTTCAGTCGGGACCCGACCACCAAGATATTCTTTCGTCCAACTCCTCGTATTCCTCGTCCGGTCATTCGTCGGGGGCGGTACGGGAGACATAGTCGGGGACGGAGGTCCAAGG

>P218*

GTACCCTGCTTTGAG

>P219

AGGGAAGGCGGGGCACTGGGGAAACCTTGGCGGGAAGGGCCCCTGCCACCAGCAGAGAAGGCGGCATAAGGGGTGGCATCTCCAGAGGGAACTGCAAGCATCCTAGCCCCAGGCAGCTGCTCAGTTCCTGCAGCT

>P220

TGTTTAGTAATTCCCAGGTCTAGAAAGACGGGATTGTAAAGGAGACACCCTCTAGTGTCTTCGGTGGGTCTTGTGGTAGGGTGGATAGGGGGTTGGGTGGGTAGG

>P221

TTAAAGTCTCTTTTGATAAAAAAATAAAAAATAATCTATAAAAGAAGTAAATGCAAAGTTTACGATAGGGGTTTCGGAGGATATGCGGCGGGGGGACGGGACGAGGGATTGGGTGGGTGAGGACGAAGGACCGGG

>P222

TAAAAATTCTTAAAATTACATACTGAGTCATATCGATTTTGAACGGAAAGAACTGTAGGTGGGTAGTGGAGATTGAGAGACTGGTGGAGTACAACAGGGGTGGGATGGGGGACCTTAAAT

>P223

TAGAAGCTAGGGGTTTTTATAGAAAAGGGGCTGGGGCTCAGGGAGGAATTGGCACGGTTTCACATGATTGGTTCATTTAAACATCAGCAGACTGTACATGCAGATAACATTTAACTTAGGTCAGAAGGGCAGGAG

>P224

TTTGGTTGGTGGTTTAGTCCCTGGGAGCTCGGGGGTTGGGGGAGGGGGTCTGGTTGGTTCACACTGTTGTTCCTCCTATGGGGTTGCAAATTCCTTCAGCTCCTTCAGTCCTTTCTCTAACTACTCCATTGTGGACCCCATGCTCAGTCC

>P225

AGGAGGAGGCTCTGGGTTTGGGTGGAGGGTCACAGTCACCCCTGTCCTTGCCAACACTTACCAATGCCAATGCTGAGGAAACTGAAGATGAAGTCTATCTTATTCAGGCTGCAACAGACT

>P226

TATACTTTCACCGGTAGTTTATGAACGCTTTCTCCGATCTGTACCGTTGAAACGAGAATGTCTCCTTGGTTGGTACACACATTTCCTACGGGGCGGGGGGGGTACCTATAATCAATTTACTTGGTCATATACACT

>P227

GAGCCACAGTAAAGGGCCCCTGTGAGGTGGGGTAGCTGGAGCAGGGGGGGTCTTAGGGTTTATAAGATGTCCAAC

>P228

TGTAGATAAGATATCCATCATTGACACATATTACACACTGTCAAGGAGAATAACAATATTACTTAAAGTCAGTAAAAGGGCGAGGGAGTGAGGGGAGGCGGATCTTGGGTGGAAGAGTCGTTCGGGGGACGGTGAAGGCACCGGAGGACC

>P229

AAGGGGGTGTGTGGCAATGGTGGGTTGAGGCCACTCGGGATGGGCAGGGAAGATCAGGGCAGGGCTGGGTCTGTTTTTCTACAGCTTGGTGTTCAGGTGTGCCTTTCTGTTCTAACCACCGTGAGAACCACCGTAGTCCCTCCTTTCCCTTAGGAAATCTGTCTTTTGCTTGTGCCCATGTGAAATATCAAAGCA

>P230

TGACTGGAGAGAGGTCACCCAGCTCCTGAGGGCCAAGCTGGTGCTATTTCCACAACACAGAGAAGCCATAAGTGC

>P231

AGGAAGAGAAGAAGGGGAGGTAAAAAGAGGGGAAGAATTAGGTATGGGAGTAGATGGAGGAGATGTACAAAGGTTCAGGAAATTGAATAGAGGTATGTAGCAAAGGGGGATGGAGAACTGGGGGTAGCAACCAGAAAGTCCCCAAAGCCAGGAAAGCAAGAGCCTCCCAGGATCACACAGGGATGACATTAGTGGAAATA

>P232

CTAAGCGGGGGTTCAAGGTGGGGCAGAAATTGCGTGGGTTAAGGTTTGATGAGCCTCTAAAAAGTGTGTTTTTTAAGATAAAACATCTGTCAATGTAAGACATAAAGAATAGACAAAATC

>P233*

GTACCTTATAAATGGAAAGGGTTTTTCATG

>P234

CAACCGAGGACTCTGACCGTCTCGGAGAAGGCACATGAACTCTAATATGTGTTAATCTGAAGTCCATGTATTGGGGAGGGTGGTATGAGTTGGACGGGGTGTGTC

>P235*

CTTTAACTAAGGGGC

>P236

AGAGGGTACGACCCTCGAAAAATGGACCAACGAGCCACAACTTGACTCCAGGAGTGTGAACGGGTCGTTTGTGGAATGGGTAAAATGGATAAATCAGTAAAAGGGTCGGGGAAGGGACGGGAAATTTAACTTTAT

>P237

CGGGCCGCATCCCGTCGTCGCCCTGCGGCGTCCGCTCGACCTCAGGTTCGTCGAACCACCGGCTTGGGTTGGAGAGCGCGACGGCGGCGGCGGCGGCGGCGGGGACGGCGACGGGGGCGGCGGTAGTGGCGGCGA

>P238

TGGAGACTAATGTATGATATGTGTGTGTCTCTATGTTGAAAAATTAGAAAATCAATCAATCAATCAATCAATGTGAGATATAAAATAAGGGGAGGGTCAGATGGGAGGCTTACCAGGTGTAAGGCATGGAGGAAATACGGGGGGTACAGAGGTGTTCCTACAGGG

>P239

TCATTTAAAAAGATTTAGACCATGATTTGTGGGGGTGGGGAGAAGGGAGGGAGAGGGAGGGAGGGGCAGGTAGCACACGTGTGGAGTCAGAGGACAGCTTTTGGAAGGCAGGATACATCTCATCACCCACTGAGCCATCTCACTGGTCCA

>P240

GGGGTGGTGAGCACACTGCGTGGGGGGGGGGGAGGCGCGGGCTGGAGGTGGGGTTGGAGGGGGGATTGCGCAAGGCCGGGGTTTCTGGAGGCCGCGGCCGCGGTGTTTTCCGCGAGGTTATTATGAGCTGAGTGT

>P241

TTAGAGATGGGGACATCATTTATGTCTGTGAGGGGCTGGGTGTTGGTGGGGCTACAATGCTCACCTTTCTTTAGGCCCTGCCTGGACTTCACCCCGCTGAGTGGTTCCTTTGAGGCCAACCCTCCGTTCTGCGAG

>P242*

ATGTGGAAGTCAAGTTATTTCTTGCTCTCTTTTGATATTGGTTGT

>P243

CTTTTCCTTCAGTCTCACATACCTCGATCACCGTGCCTACTTGACAAAGAACAGCACAGACCTGAATGTCCAGGATCAAATGGCAAAGAAGGGGTGAACGGGTGGGGACTCCGTCGGGCACAACAGAAGAAGGAC

>P244

GTCATACTCACCGGGACGAATCTGAGGTCGTAGGGAGACTGTCGGGGGGGTCACAAAGTCTTGATGCACCTCAAG

>P245*

CCGGTTTCAATCATACTTTGGTTTGTTTTG

>P246

CCCTTTCGTGTCAAGACCCGAGAGCCCCCCCCCCCCCATAGAGAGATGAGAAGTATTTAAAAGTATAATACAAACATTTTCACCCGTCGTGTCTGTACTCTACTCACTACCTAGGGAGGAAGAAGAAGAAGAAGAAGAAGAAGAAGAAGAAGAAGAAGAAGAAGAAGAAGAAGAAGAAGAAGAAGAAGAAGAAGAAGAAG

>P247

TATTTTCATGTAGGCTCACCATTCTTGGGGGGGGGGGTGGTCCTTCAGTCAGGGTATGGTGGTGTACACTGCATTCCCAGCATACAGAAGGCAAAGGGTGGACGATTCTAAGTTCTAGGACAGCCTGACCTTAATAGCAAGACCTTGTCTGAAACAAAACAACAA

>P248

TCCTAGACGAAAGTGAACAGACCTCTGACGGTTAGACTGAAAAGGGTAGTTCAAAACCACCACCACCACAAAGGGTCAAACAAAAGAGGGGGGGGGGGAAGGGGGTAATTACATTGACTTCCTTAGAGAAAAATT

>P249

TAGAACACAGGCGTGGAAGGGAGGCTTCGGGCACGGGTGGGATGCTGACTTACGAGGTGTTTGCTGCAGGAATGGCTGGACTGGCTGGCTTAAATTTGCAAGAGAACCTGGCAATTCCAGGCTTTCCCAGCCTCT

>P250

CCAAACTGAGACTTGTCTGGATCCATAACAGGTGGCTGGGGGTGGGGGAAGTCCTCATCAAAGCACACACTATCCATCTTTGAATTATGGCCCCATCCTCCTCAAGCGCAAACTATCTCT

>P251

AAGGAGTAAGAATGTGATGGGGTGGGGCTGGCTGTCAAGAAGGTTGGGATGTTAACAGAT

>P252

ATAAAGAAAAAAAAAAAAGAAAAACGTCAAAAAAATTTAATCCATAAATAAAGTAAATGTAAAGGTTACGATAGGGTTTTAAGGGGGTGTGCAAGGGGGTGAGTGAGGGGGTGGGTGGGTGAGGGTGAAGAACCG

>P253

ATACGTACGAGTGGATAGAACAACTGTGTACATATGTCTACATACACGTTCATGGTGCTTGTACACTTCCGGTCTTCAATGGGAACCCACAGAAAACATACAAACGAGTCCTAGGGTGGGAGTATCAACGTTTTTCCCCAAAATAGACCTTGGTAGAAGGGTCGGGAATCTTACACAGACATCAGTGGTTGTGGGTTAGA

>P254

GGTCAGGTAGGTAGTCAAGTGGGTAGTTAGGAGATGATGGATAGTTATGAATGAAGGAAGTGGATGGATGGATGGACGAGTGGGTGGTTGGGTAAGTAGGTGGGTAGACAGTGGGAAGGAGAGAAGATAAGGAGA

>P255

AAATGAGGGAGGGGTGAAGATCTGCAGATAGCTTTCCTATTTCTACCCATCCTCCCATTGATGCCCAATTAGGCCATCCCCCATGTGCAT

>P256

TTCTAGAAAAGAGATGAGGAGAGAGGGAGGGAAGAGAGAGAAGAAAGGTGAAAAGTAGGTGAAGAAGGGAGAGGGAGAAGAAAAGGAAGGCAAAATAATGTATTGAGAATATATACTATATACACACACTCATATGAAAACACACAGACA

>P257

CGGGTAAACAGACCGAAACCGAGAACTTTGGACCCTAATTACCTTACATGGTGGTACTACCGGAAAAATCAGAAGAAAGACGAGAGAGAACAAACACAGGGGAGGGGGGTAGAGAGGAAA

>P258

GAGATGGAATGGGGATGGGGAGGGGCCATCGTTCCAGTGAGGAGCTGTTGATGGGGTAAGGTGTATGTAGTCCAGAGAAGAATCCGAGGTATACAAGATGTTTGTAAGTCCTTAAGACCCTGCCACAGTGACAGAAGGAAAGGGCAGAATATGCCTATGTACCAG

>P259

GTGTGTGTGTGTGTGTGTGTGCGCGCGCGCGCGTGCGTGTGGTGGGGGTGGGGGTAGATACGCGCCTTGTCTGTGCTAGGCAAGCGTTCCATTGCTGAGCTACATCCCAGAGTCTTCTTGACTCCCTAGTTTCCC

>P260

AAGAGGAAGAGGAAGAGGAAGAGGAAGAGGAAGAGGAAGAGGAAGAGGAAGAGGAAGAGGAAGAGGAGGAGGAGGAGGAGGAGGAGGAGGAGGAGGAGGAGGAGGAAGAAGAAGAAGAGGAAGAGGAGAGAGAGAGAGAAAGAGAGAGAGAGAGTATGTGTGTGTGTGTGTGTGTGTGTGTGTGTGTGTGTGTGT

>P261*

GGAAGTCTCGGACTTTCGGGACCATTCCTAGTTTTCGAAGCCGTT

>P262

CTTCATTACTGTTTAAACTCCTTTGTTGGGGCGGGGGGAGAATTATGAGCTGGTTATAGATGCTTCATGAATGCCACAGAACCAACGTCCTGCTTTAGTAGCTAGATCACTGGTTACGAAGGAGACAACTATAAG

>P263

AAGTCTTGATTACTTAAGATGACCAGACCTCTGACCCCAGACAAAGAGTTCCGTTCTGAAGGTCTCTAAATATACCGAGACACGACCTGTGGTTCGTGGTAGGGCGGGTAGTACTTCGTCAGGAACTTTCAGGGAGTCGTGACTCTCCGA

>P264

CCAGGGCACAGAGGGTAATGACCCCAGGAACAATCCCAATGATAGTATCTAAGGTAACTCAAAGTGAACTTGATCTAAAGGTGGACGGGG

>P265

CAGGGTGGGGAGAGGGTATAGGGAACTTTCGGGATAGCATTTAAAATGTAAATAAAGAAAATATCTAATAAAAGAAAAGAAAAAAAGAAA

>P266

GATCAAGGGGGGGAGGCCCCTTGTGGGTGGGACCATCTCTGGGTTGGTAGTCTTGGGTCCTATAAGAGAGCAGGCTGAGCAAACCAGGGGAAGCAAGCCAGTAAGTAACATCCCTCTATG

>P267

GCTACCTATCTCTAAGATTTATAGATGGGCAATAGGGGTGGGGGGAATGGGACTGGGAGTTGCTGTTGTTTATAGTAATATGTCAGCCTATAAGTTTCCCGTGCTCCTCTAAGAAAATTCAATTAAACTCATTGACTGATCAAGCTAGATTTGAGTAGAACTATT

>P268

GTAGGGAAACTACACCCTAGAAAAAGCAAGAAGGGTGGGTGGGTCTAACTACAAAAGCAGGTCTAGCTGTTAGGCTCTGTTTCTTCAGCTCTGGGTTTTTAGGCCATGGGGCAGCAGAATGTGTGGTGGCTCCTGTCGAGGGTCATCTCATTCAGAAGAGGGTCA

>P269

TAGCTGAAGAGGTCAAATGTCCTGTTGTGGGGTTGGGGCAGGGGAGGAGAAAAACAAAGTTCAGATTAAAACCTAATGTTCCAGACTGCT

>P270

AAAGGACACCTAGATCCTGGGTTGACTCCAGACGGACACCGTATAAAAGAAGTTAATGTAAAGCTTAAAAAGATGTAAGGACAAAAGGGGAGGTTTTTTGTGGGATGGGGTGGAGAAGAGAGGAGGGGGACGACTGGTTGGGTGGGTGTGGGAGAAGAACCGGGA

>P271

GAAGCCAGAGATGAGTCCAGAATGAGGACGGGGAGGAGAAGGAGGAGGAGGAGGAGGAGG

>P272

AAAATCACGGGGGAACACAAACCATCGTTCGATTCGTCGAGAGACAAATAGAGGACAGAGGACAGTGGACAGGAGCCAGGGGGACAGGGACAGGGAGAGGTTTAACAGGGAGAGGTGTCGGTGAGACAAGGTATT

>P273

TTTAAAAATTATGTATTTTATGGGGGCGGGGCTCATGTGCCATGGAAAGCATATGGAGATCAAAAGACAATCTCCAGGGGTCAGTCCTCTCTCTCTACCACAGAG

>P274

CCTTCAATGGACTGTAGGTCCTAACCCCTGTTGTGTCGTCCGAGGTCTCCACGGACTCTCCGGTTAAGATAATAGATCGATGGAAGGAAAGAGGAGGGAGGAGGGAGAGGGGAAGAGAAGAAGGAGAAGGGGGAA

>P275*

GTTACTGTGGGAAAC

>P276

CTTCGTGGAGGAAGGGATCCGACTCGAGGTCCCAGGACCGGGGGTCACCCTCGAAGCACAGACGAAGTACATGTGAGGGTAAGGACCTTGGACCGTCGTAGGGGCCTGGGGCGGACGACTAGGACAGGTAAGTAGGGAGAGACCCCGGTGAAGGTTTCGACGGGACCCCAACAAGACAGGTGGGTGACTCGACTCACTGT

>P277

CATCGGGTTACTATCTTTACATTGGGGGCATTGGGTTCAAGTTTTGGCTTGTCCGAGTTCAGAAGCCATAAGTGGTGCGCAAGTAGGGTTCAAGATGAGGTCTGCCTTCTTTAGAGCTGGTTTTAACACATAGGA

>P278

AGGGGAGAGGAAGGGGGAGAAGGGGAGGAGGGAGAAGAGAAACAAGAGGGAGAAGGGAAGGAGAAGGAGAAGGAAGAGGAGAAGAAAGAAGAGAAGGAAAAGGAAGAGGAGAAGAAGGAGAGGAAGGAATGGGAGGGAGTAGAGAGAGGAGGGGAGGAGAAGAAGAAGGAAGAGGAGAAGAAGAGGGAGAGGAAGGACAG

>P279

AACCTCCTCCAAACAGTAACCCTACCCGTAACACCAAAGTTTTCAGGTGTGGTCCAATTCAGGGGGAGAGGGACAGGGAGGGGGAGGTGGGAGAGAGGGACAGAG

>P280

TACATAAGAGGAGAAGGAGGAGGAGGAAGAAGAGAGGGAGATAGG

>P281

GTGAAACATCTGGTCCGATCGGAGCTTGAGTCTTTAGACGGACGGAGACAGAGGGCTCACGAAGCTCCGGGCGGGGGGGCAAGAAGAAGAGAGAAAAAAAAAAAAAAAAAAAAAAAAAAC

>P282

GGCGGTTTTCTTTCCGTCGTCTCCCGGTTCGTCGGATGACTACGTTTCGGGGTCCGACCCGTGCTCACAGGAGTCCCACGCACCGAGGACAGGTAGGGAGGGACGTAGGGTCGGCCGACCTTTAGACCGAGGACCCCTGTCACGAACGACAGAGGTCTCTTTCTGTCACCCATGTTTCTCCTCACACGGGGTCGAGTGAC

>P283

GAGAGAGAGAGAGGAGGAGAGGAGGAGGAGAGGTAAAGAAAGAGAGGGTAAGGGAGTAGGAGAGGGAGAGAAAGAGGGAGAAGGAAAGGG

>P284

GGGAAGAAAGGAATTGAAAAGATAAAACGAAAGTACCGGAGGGAAGAGTAAAGAATAGGATATGAGGAGGAGAGAGTTAAAGAGAGAAGAGAGAAAAGAGGAGAGAGAGAGAGAGAGAGAGAGAGAAGAGAGAGAAAGAGAGAGAGAGAGAGAGAGAGAGAGAGAGAGAGAGAGAGAGAG

>P285*

CGAAGAACTTTCTGAACCTAGTTCACTCGGGTTCAGGTAATTACCCGATACTTTGACTCTCACGTCGGGTTAGAGAGAGAGAGAGAGAGAGAGAGAGAGAGAGAGACAGAGAGACTGAGA

>P286

GGAAGGGAGGGGTGGGGGTGCGGGGTGGGGGTACGAGGTGGTCAATTCTGTCAATTCTGA

>P287

AACTCTCAATTGTATGTAAGTATTGGATTGTCTTTAATAAGAAACAACATTTCAATAGGTAAATGAAAAAATAACCTATAAAAGGAGTAAAGGAAAAGTTTACAATAAAGGAAAGGTTCAAAGAGGAGGGGTGACTTTGGAGGATCGGGGAAGGGGTGGGACGAA

>P288

CAGTCTGGATCGGTTTGGGTTGGAAGGACTGAATAAAGGATCATGAGGGGCATCGGAGGGAATTCCTCGTTGAGGGTCGACAAGGAATCTGTGGTTTGTGTCAGGGTAGAGGGAGAGGGGTCTCCGTCGCGTTCGAAGGGAGTGAAGGAC

>P289

CCAGGACAGGGAAGCAGGAGTGGGTGGGTTTGTGAGGGGGGAAGGTGGGATGGGATAGAATGTTTTCAGAGGGGAAATGAGGAAAGGAGATAACATTTAAAATATAAATAAAGAAAATATCTAGTAGAAAAATAGAAAAAGAAAAATGCAGAAATTTGTAGACAC

>P290

ATGGGGGAGGGGCGCTGCAGTATGTGGGGGCAGCGGGAATAATGGCCTTCTTCCAGGATATCTGTAGGTCATTGAGACAGTAGGCTTTTGCAGGGACTCCAGTGATGAGGAGAGTCCTCA

>P291

TCCCGACATGGGAGGAATGGTACGGAAGTACGGGTCTGTGATTATCGACAGTGGAGTGTTCCGGACCACAGTATGGGAGTCAGACGGACGGACAGGAAAGACTGGGTGAGGGAGGGTGAAGATCGGACAGTGTTCCGAGACTCTGGAAACTGGAGGACTGGGGAT

>P292

ACAACGACTCTGGGTGTTGTAGACGTTTCCATTATTATCGGAAACTTGGTTCTACACAAACAGAATTTTGGAGGCGTGGTTCATCGGGCAACGACGACACTAAGTATACCTTGGGATTTCTTGGACAAGAACAAGGGGTGGGATAGGGTCTTTTTACGGTTTTAT

>P293

GGCGGGGCCGGAAGTTTTAGATGTGAGGGGGGAGGGGGGGAGGGAGAGGGAGGAAGGAGACTAAGGAGGAGGGGAAGACAAGACAGAGGGACAGAGAGAGGGGAAGATGGACGAAGAGGAGGTGGAAGGTGGAGC

>P294

CGAGGGAGGCACATGGAGTCAACCCGGGACACTGTCGGACTCCTACTGGAACTTAAGGACTGGGAGGACAGAGATGGAGGGTTCATGGTC

>P295

AAAAAGTAGAGGAAAGAGAGGGGTCCCGGACGGGAAGAAACAGGAACGAGACTGTCGGTCGTGAATAGAGCCCGGAGGCCTCGGGCACGGGTAGTCGGGAGGGGGACCGGTCCCGGCGCC

>P296

GAGAATGAAGAGTGAGAATGAAGAAAAAAAAATAATAATGCATAAAAGGAGTTAATGTAAAGGTTACGAGAGGGTTTTCAGGGGGTATGGGAGGGGGGGTGAAGGGACGGGTGGGTAAGG

>P297

GACGGAGGGTTTACGACCCCAATTTCCGTATGTGGTGGTGACAGAACACGGTTAAAAATTTTAATTAATTAATAAAATAAATAAATGTGGGGTGAACGACGAGAGGAGGTCCAAGAACGTCTCAAGAAGGAGAGTAGGGGGGAGGGGAAGTGTGAGGGGATCCAAAGGAGGGTAAAACCC

>P298*

GCATTGAAAATGTAAACGAGGAAAATACCTAATTAAAAAAAAAGAACGCTTCTTTGTAGCAATGACTTCAGAGCG

>P299

TACGGAAAACTACCTCTGAATGTCCCCTTCCTCCGTACCGAGTCACCGTTTCTTAAACGGACCGTATCCACTCGGAAGTGCAAAGGGGGTACAAGACCGGGGTCGTCAAAAGGGAGGGATGGTGTGTATTTTTTT

>P300

GGTGTGTAGCAGTGGGGGGTGGGGAACTGAGAGTAGCCACTAGAAAGTCCCAGATGCCATGAAAGCAAGAGGCTCCCAGGATCCAATGCGGATGGCATTAGCTGA

>P301

AGAGAAGAGAATACACAGCCTGGGGCAGGAAGGGGATGAGGGAGGGGACGGGGAGGGGGGGAGGTACAGAGTGCAGGGAGGATGCATGGGTGGAAGAGGTTCCTCTGGTAACTTGGAGCAGAAAGTAGAAGGCCG

>P302

TGAGGAGGGGTCAATGGATCGTTGTCATTCTATTCTGTCGTATGAAATTTTCCCCGACAAACCGGAGAGGAGTGAGAGAGAATGAAGAGTGAGAGTGGGGGTAGAGAGAAACAGAAGAGGAGAGGAGAGAGGGAGAATGAGAGAAAGAGAGATCGGAAAGGAGAGAGAGAGAGAGAGAAAAGATGGAAGAGAGAAAGAGG

>P303

AAACGGAAACCTTTTTCTTTAACAATGAGTTAACTACCGAAAGGAGTCGAATTGGTGTACGGCTGAAGGTGGGACGGGACCAAAGGGTTCAGTAGGGACCGATAC

>P304

AAAAAAGAGTAAATGAAGGGATGGGGGAGGAAAGAAAACAGAATAATGAAGGAAGAGATGAGAAAAAAGAGGAGACAGGGTGGGGAGGAAAGAGGTCAGGAGAGGAAGACTCACAAGTATGAACCACTCGGGATTCAAATCCCTTCAGGTATGAAGTTTAAAAAG

>P305

GACGTCCTGAAGAGGAGAAAGAGGTGTAGGAGGAGTTGTACAAGA

>P306*

TATTTGTTGACCGTAAAAAAACACACAAAGATTTAGTACGGTTTCAAGGTGTGTCTCTTGCCAACACCCGAATAAATCGACCCCAGAAGAGAAGAGAAGAGAAGAGAAGAGAAGAGAAGA

>P307

ATGGGAAATGTGAGTCTTCAGAGGGGGTGTGGGGGAAAGGAGAGGGACCCCAGTATTTTAACATCTACCCAATTTCATATCTTCCCCCTACCCCCCAACAGCTCA

>P308

CATTGACATTTTAGAGAGAATCCTAGTCCCGTCCCGTCCCGTCCCGTTATGTGGAACGATACTTTCGTCCACGATCGGGGTGGGTTCGAGTCGGGGGAGGAGGAGGAGACGTCCCGCCCT

>P309

ATAGTCGTTAGTGTTCTCTTTCGATCTGGCAAGAAGTGCTAAAGGAACTCAGGACGTCCTCTCTCTCCTTTGGTGTACTCAAGGGACATGGGGGAGGTGGGACGGGTTTAGGAGAGTTCTCGACACATTTGAAAC

>P310

CAAGTCGTGAGTCGTCGTTCGGAGTGTAGAAAAAAAAAATTAAAAAATAATCTATAAAAGAAATAAATGTGAAGTTTACGATAGGGCTTTCAAGGGATATGGGAGGGGTCTGGACGAGGGGATGGGTGGGTGAGTGTGAAGAACCGGGACCGTAAGGGGACATGA

>P311

GAACTACAGGATGTCGAAAAAGATAATGTGACGTAGATGAATGGGAACGACCCGTGTGTGACACATGAGACGAGAAGACTCTGTACCAGGGGGTATTGGGGGCGATGAGCTTTTACTGAGAAAAATAGAGAAACT

>P312

CCACCCAATTGGGTCTTTACCATGGGCTGGGGATGGGGTTGGGGG

>P313

AGGAAGGAAGGAAGGAAGGAAGGAAGGAGGGAAGGAGGGAAGGAGGGAAGGAGGGAAGGAGGGAAGGAGGGAAGGAGGGAAGGAGGAAAGGAGGGAGGGAGGGAGGGAGGAAGAGAGGGAGGGAAGAAAAGAAGG

>P314

TGTGGGAGGATGGGGAGGTGGGTGGCAAACTGTGGTTGAGGGGCTTGTTGAGTTCGGTGGGTGGGGGAGCACTCTCTCAGAGGCAAAGTTAAGGGGGGATGTGATAAGAACTTGTGGATGGGGACCTGGGGAAGGGGGCAATATTTGTAATATAAGCAAATAAAACAATTTTTTAAAATTAAAAATAAATAAATAAATAG

>P315

GTCGACTCTCTCGAGTGTAGAGTTGTCGTGATTCGTCTCTCGCGATTGATCCTTACCGTGTTCAGAAAACTTTTGCAGTTTCGGGCGGGGGTCACTGTGTGGAGG

>P316

GAGGGAGAGAATATAGGGAAGAGGGGACATTGTGGAAAGCTAGAAGAAACAGAGGGATAGGAAGGTAGCCAGGATGTATTGTATGAGAGAAGTATCTGTTTTCAA

>P317

AGAAATAAATGGTGGTGGTGGTGGAACACAGACTAAAAATTTTAGTAAAAAGAGTGGAGGTGACGATGACGTGTCAGGGATTGGATGGAGGGATTTAGGTGATAAAACATAAGGGTGGGGGTGTGGAGTTGGATGCCTAGGAAGCTCCTT

>P318

TTTTTAAAGAGACTAGTTCCGGGCACTCAAATCAAATGATTCTCTGGCACGAATATTCCCCCTTCCGGGTAGGGGGTGGGGTGTGGGGGGTGAGGTAGGAACAAC

>P319

AACGTATTTAAGTAAGAAAAAAATTTTTATAAAATAATCCATAAAAGGAGTAAATGTAAAGGTTACGATAGGGTTTTCAGGGGTTATGGGAGGGGGACTAAGGGGATGGGTGGGTGAGGG

>P320

TCACCTTCCATGAGATTCATCATCTGGGCCAAAAGGAAGCTGGGAACAGTACCAGTAAATTTGGTAGGTCAAACCACTATACTTCGAGGGAGTTGGAGGGTGGGAGGGATGTGACACTTCGTTGGTGTCGATATA

>P321

CACCAATACTCGGTGGTTCACCAAAGACCCAGACTCAAGTTTGAGTCCTGGAAGCCTTCTCGTCAGTCACGAGAATGGGCGACTCGGTAGAGTGGGCGGGTCAAGATATGAAAAATTTCCGACTGAGCTGAAGGT

>P322

CGGGGGAGCTTCTGGTGGTGGAAGTTTTGGGGGAGCAGTTCTCTTCCCTGCCAGTGTTGCAGCTCTTATGACAGA

>P323

TTGAAACGATTGTGTCATTTCCCAGTGGATGAGTGGTTGGTCCAAGTTGATACAAGATGGGAAGAATAAAGTAAAGTTAAGGTGGGTGGGGTGGGGTCTGTGGGGAGGTTGTACTCTCTG

>P324

GGGTATACTTTGAGTGGGCTATAATTGGGGAGGGAGATTTTACCTTAGGCAGTATATAATTAAGTGCCAAACTGAGTGGCAGTCTGTGCTCTGGAGATCAGAGGCAGAGAGATCAGTGGGAGCTGAGCAACTGTG

>P325

TATACATTTTGTTTATTTGGAGTGTGTGTGTGTGGGGGGGGAGGGTACAAGGTCCAAATATAGTCTAGGTTGCAAGCATGCACCACCACGGCCAGCAGACAGATA

>P326

AATGCTAGGGCAGTGAGACAGGAGTGGATGAGTGGGTGGAGGGGCACCCTCATAGAGGGAAACATGAGGAGGGAGGGGAGATGGGATGGCAGGTTGTGGAGGTGTAATAGGGAAAGGGGATATCATTTGAAGTGCAAATGAATAAAATGATTAATTAAAAAAATTAGACTCATAAATGCA

>P327

CACTTTCCTCCAGGTCGGTGTGTGAGTCGGGGGTGGGGGTTGTTGAAGATAGACTAGCAATAAGAGGATGCAGGTGTTGCGCGTAATGGAATGAGTTCAGGCTTA

>P328*

CTCTTCTGTAGGTGTAAAAGAAAGAGATTGGAAGTAATCACGTGAACCGTGTACACGAGAGAGAGAGAGAGAGAGAGAGTATGTGTGTGTGTGTGTGTGTGTATG

>P329

CTTATTCCTGTTCTGAGGGACAGGGTGGGAAACGTGGCCCAAATGCCAACTTTGGAAACACGACCCAGGTTCCTGTGATGGCTGCCACTCCAGGCGGGGTTCTCTGGTACTTGGAACCTCCTCTGTGCCAAAGGAACAAGAGGTTAAGTT

>P330

ACCCCAGTGTAGGGGAACTGAGGGTGGGGAGGAAGCAGGGGGAAAGAGAATGGGATAGGGGGTTTCTGGGAGCAGGGGGTGGGGGTAAGGGAACCAGGAAGGGGGACAACATTTGAAATGTAATAAAGAAAATATCCAAGAAAGAGAGAGAGAAAGGAAGAAAGA

>P331

GGCCCCAGGGCAGCTCAGTCAGCCCTGGAGCTGAAGGGAGGAGAGGGCAGGGAGAGAGTGGGAGGTGCTGGGTGGTTCTCACATGGGCAAGAGTCCTTGGTCAGGTCCGTGACTGGAGCTCATGAAGGCCTTCTGGTGGGAGTTTAGACTCAGCTTGTTAGGGGA

>P332

CACATGAGCTGGTGAGTGGTGGGGAAGATGGGTGAGGCAGGAGAGTAAGGACATAGCACGAAAGATCTTGCTTTTGGGCATGGTGATGCACTCTGTAATCCTAGCACTTGAAAGGCTGAA

>P333

GGAAAAAAAATGAAAATGTGAGGTGATGGAGTGGGTGGGGGAACACCTTCTCAGAGGCAAAGGGAGGAAGTAGCGGGTGAAGAACTCTGGGAGGGGGAACTGGGAGGAGAGACATTTTGAAAGTAAAAAAAGAAAGAGAGAGAGAGAGAGAGAGAGAGAGAGAGA

>P334

AGCAGTGTCATATGAGAGGAGGAAACGCTTTGTGCCAGGCTCTGGGCAGCTTTCAGAAGATCTGATTACACTTATCACGACGTGTTACGG

>P335*

GTCCGATATTGATGGTCTCGACTGAATCGA

>P336

CTGAGTCTTACCTCTAGTCTTAATTTTAACAACAGTACGCAAGGTGTTCGTGGCCAATACCTAGTGCCACCCTTATCTTTTCTCCTGTAGGGACGGAGGGTGTAGACGAGGTGAAATAAA

>P337

GTTTAGTGAACTCTAGAACGTCAGGTCTTTCTAGTCCATGACCACTGGGGACGTGTTTCGGACAAGGGTAAGGGTGGCATGAGACAGGGAAGTACAGGAAAAGGGAAGAGGGAACAGCGGAGGAGGAGTAAGGAAGAGGAAGGAGAGGAAGGTGAGAAGGTATAGGGGTGGGCGGGGAATGAAAGAGAGAGTGGGGCGGG

>P338*

GCTGCTAGATGTAAGAGAGGGCAAATTGTTCATGATCTGAAACCTTGAGACATGGTTACT

>P339

GGGGTCCCCCACCCACTCAGAGGAGAAGGGGAGGGAGGATGGGAAAAGAATTGTGGGAGGGGGCAATGAGTGAGATGGAAATTGAAGAGGTAAAATGATGAGATGGTGGTGGTGGTGATGAAGATGATAAAAGCCAGTATTGCTAAGTTTTTTTTTAAAAATCTGTCTGTAAAGCATTTT

>P340

AAGATGTGCATCAGTCAGAGGGCTGGGGTTGGGGTGATGGGGGACTTCCAGAAAAAGCTAGAGTTGTATTGTATCCTGTAGTTTCTGGGGGTCTGGAAAGTATCAATGTAAGCACTCTAATATCAAAGAAATGCT

>P341

GATACCTTCACGAGTCGACGACATCAGTAGTGATCGAGGGGGAGGAATAGGGGAGGGAGG

>P342

AAAGGGAAAGGACTGGTTGGAAAGATCCGAAGACCCCTTTGTCGACCCGTCCGGGTCGACACAAAAGGAGGAAAGAGAGACGGGGCGGGGGTGGGGTCGTTCCTTTTCCTTCTCCTGTTA

>P343

CTTCTCTCATGTCCTATATGTTCTGTTTCTGTGTTAGGTAGATAGAATGGGTAGATAGATGAGTGGATGGGTTGGGAGGGAGGGAGGGAGGTCGGTGGGTGGATAGGTAGGATAGGTAGG

>P344

GATCAGGATGCACAGGTGAGCTGAGGGAGCCCAGGGCGAGGGGGAGGAGGATGAGTGAGGTTTCTGGTACCATCGGTGGTAGTGCTCCAACAAGAGCAGAAATTGTTCCATATGAGCCAT

>P345

TAAAAGAAAGAAAGAAAGAAAGAGAGAATGGAAGGGAGGGGGGAA

>P346

TCTGAGGAAAGGTGTACAGAGAGGACGTAGTGTCCGGTTTCAAATCCCCAACTTGAACGGATCGACATTAGTCGCGGGGGTATCTGTGAGTCATCCGTCGGACCATGGTGACAGCGGACGGAGGTCTCGGGTGGG

>P347

GCTGCCCGCCCCGGCGCCCCGCCCGCGCGCGTTCGCCCCTCGCGGGCCCCGCCGCACGGACGCCCCCGCGCGTGCGCCTCGACGCCCCGGTCCGCGCCCCGGCCCCGCCGCTTAGGCGTCGAGGGCGGCGGGAGGGTCGGGTTCGCGCGGGCAGACCCACCCCCGGCGGGCCCTCCCCGC

>P348

CAGATGGAGGGAGGGAACTGAATGGGAGGGGAGTGGGGGGGGCTTTTCAGGATCGTGTGTGGGGAGAGACTGGAGAGATGGACAGATGGTCGTGAGAATGAATGGAAGTCTGCGGCTAGT

>P349

TTTAGTCACAGATGAATTTGGAGTGTGGGCCCTGCCTGGGCTGGGTGCTGGGAGAGAGAC

>P350*

AAACCAGGAAAAGGGATAACATTTGAAATGTAAATAAAGAAAATACCTAATTAAAAAAAAACACAAAAAAGTGTT

>P351

GAAACGACGTTGTGTCCCCCGGCCCCCGGAAGACGTCTAGATACGTGACAGTAGGGAAAACAAGGAAAGATTGAGTCGAAGTCCGGGGTGGGGGAGACTCGGAACCGTAAGAACTCAATT

>P352

GGTGAAGAGGTGGTAGAAGAGTAGGGGTCCAGCGGGGATAGCAAGCTGCTCAGGGCAGTTGGAAGTGCCTGAGAGGATTTAGGGGTCAGGGAAGATGGGTGCCAGGATGGAATCCAACCTGTATGGTTCTGGATCAGCAGGTCTGATGTAGGTAGATGGAAAGGT

>P353

ACTTGGGTAGCTTAGGAGATGGGGTAGACTTGGGTAGCTTAGGAGATGGGATAGACTTGGGTAGCTTAGGAGATGGGGTAGACTTGGGTAGCTTAGGAGATGGGGTAGACTTGGGTAGCTTAGGAGATGGGGTAGACTTAGGTAGCTTAGGAGATGGGGTAGATGGGTAGCTTGGGAGATGGAGTAGATTTAGATAGATT

>P354

GGGAGAGTGTAGAGTGGGTGGGGTTAGAGGAGTGATGTTAGGGAAGTCGGTCGCTGTGTGTGTGAGCCCAAGGAAGTGGGAATAAAAGCCAAGAGTGTCAACCCA

>P355

TCCCTCAGTAGACTCTGACTTGCCAACTTCTGGAGGGGGGGGGGTAATAGAATTTCTTTGTGACTTCTTCTAAAGAGTCATCTTGATTTGGACACTGGTGACAGATCACCATATTCAGGGAGAGGAAGGCTGTGACTCCAGGTATGTTTT

>P356

TTCTGTGTTACTGAAGTTGCTCCGAGAGAAGTAGGAGTCCGAAGAGTTATGAAGACTCGGTCCTTTTCCGGGAGCGAAGAGTACCTCGAGATCACCCCAACGGGTCGGGTCGGGTCGGGTTATCTGCGGTTCTAC

>P357*

GCAGCCGTTCCCTGC

>P358

GAAAAGGAAGACAGGCTTGAGGGAAGGAGAGAGGAGGGAATGGAAGGAGGGAGAGAGGAGGGAGGGAAAAAGGAGGGAGGGAAAGGAGAGGGAGGGAAGGAAGGAAGGAAGGAAGGAAGGAAGGAAGGAAGGAAG

>P359

CAGGAGGAGGAGCAGGAGGAGCAGGAGCAGGAGGAGCAGGAGGAG

>P360

AAAAATAGTCAAAGCCTTATTTGGGGTTTTTTGGGGCGGGGTTAATTTTTTTATTAGATATTTTCTTCATTTACATTTCAGATGTTATCCTCTTTCCCAGTTTCCTCTCCAAAAGTCCCC

>P361

TTATTCGATGTTTGTGGTACTCTAATTTAAATATCAAAAAATCAAATTAAATTAGAATAGACATTAAGTAAAAAAGTGAGGTATAAGGTAAGGGACGGGGAGGGGTAGGTGAGAGGCTGGCGAGTTGTAGGGTATGAAGGAAGGGTAGGGTGGGGTAGAGATACACCTACAAGCATAGGGGATGGGGTGGACTGG

>P362

TGACTGGAGGGGGGGGGGAGGGAGCTCAGTCAGTAAAGTGTTTGCTTTGCAACCATGAGGACCTGAGTTTGATTCCCCAGAACCCATGTGGGGAAAAACAAAAAGAGAGAGATTGAGACAGAGACAGAGAGAGAAAGAGAGATATACAGACAGAGACAGAGACAGAGAAAGCAAGGGACAGAGAGAGACTGTAATGGTTT

>P363

GTGCCTCCCTACAAGTCATGACCGAACGAAGAAGACTGAACGAGTCGGACGAGAGAATATCTTGGGTTCTGACGGTCGGGTCTCTACCAGGGTGGGTGTTCCCTG

>P364

AGGGGAAAGTGTCATGAGGGGGGGGGTTATTGTGGAACGGGTAGAGGGGTAGGGGATCAAGATACTACCACAAGTGGGTGGGTGGGTGAGGGCAAAGGGACGGTACTGTCAGGGGATGTG

>P365

AAAAAACGACAGGTGGTCGAGGAGGTTAAGGTCTTAGGTACGTACCGAATTTCTCTTTCGACCCGTCCCGTTTGTAGGGTTGTAGGGTGGGAGGTTATGACGAGTTGGTGTTATCCCCGACAACGTTGGGGAGGGATGAATCTCTTTGGG

>P366

ACTAGGTCTACCGTTTGAAGTTGAAGTACGGAGTTCTTACTGTCGACCCTCCCCTCGACTCCAATAGGGGTGTACGTTCCCTTAGGGTCG

>P367

TAGAGTCGTCAGGGAATGTTTGTATCTTCAAAATAAAGGAACTCACAAAGGATACTGTATAAAGGTAGTAGAAGTGGAGACAATGGGAAGGGATGGGTGGGGAGGGAGAGGGACGGAGGAAGGGTCCTTCGGGGAGGAGAAGAAAGTACT

>P368

ATTAGGCCCCGGGTCCCTCGACGCGAACCACCCCGGACTCCCCGTCTCTCCTGCCCCTGAGTCGCAACTCACGAACGGGTCGGGGTAGCGGTCGGTACCACCGGG

>P369

CCTGAGCTAACTGTGGAGGGTGGGGTGAGGCGGGCATTGAGAACAACTCAAGAAACAAAATCGTTCTGAAAGATCAGTGATAGGAAGTTGTTATCCCCACCCTCAAAGGAAGCAACCCTGAGCAAAACTGTTTAGCCGCCTCCCTCTACCATTCGGCTTCCAACATGGCGGCTGCAGGGTTAGCCTCAAGACTCT

>P370

AGGGAAGAAGGAAAGAAGGACAGGAGAGGGGAGGGGAGGGGAGGGGAGGGGAGGGGAGGG

>P371

GAATTTTCCTATTCTTGGTGGTGGTGGTGGGGGCAGGGCTGGGGGCGGACCCAGTGCCCTGTACGTGCTGAGTAAGCATTCCACCATGAGGTATACCCGTGATGCACTTAGTAGTAGTAGTAGTAGTAGTAGTAG

>P372

AAAGAAGGAAGAGGAAGATGGGAGGAGTATGAGGAAGAGGAGGAAGAAGAAAGATGAAGAAGAATATGATGATGATGAATAAGCTGGTTCTAGTTTTTTTCTCATCTATAAAGCATTTAA

>P373

AGGAAGGAAGGAAGGAAGGAAGGAAGGAAGGAAGGAAGGAAGGACCCCAAGTGTTACTGCACTCCCCCAGACAGGGCCTGAGAGCTGCCACAGGTCAGAAAGCCCCTTCCTCCGCCCAAG

>P374

GTTTTATTTTGTTGTTGTTGAGTCTTCAAGTATTAATAAAGGAAAAGTCCTTCCGTCCTTTTAGTCGGGTCACTGAACAGGAAAGAAGGA

>P375

GGGAATGCCAGGGCCAAAAAGGGGGAGTGGGTGGGTAGGGGAGTG

>P376

TGATCTGTATCTTTACGTATCCGGGGTATGGTGAATGATTAAAAATTTTTTGTAAAATTAAATAAAAGATAGGGTTAGTGTCGGGGGAGGAAGGAGAGAAGATTCAAGGTGGGAATGTTTAAGAAGGGGGTGTAACGGGGAACCTATGGT

>P377

GTCAAGATTGGGGCTTGGGAGGGATAGAGACTGTATGTGGGTTCATATGACCTGTGCCCAGTGTTCTTACCTTTCATTCCTGATGTAGCCCTGATGCAGTGATGTCTTTGGTGAACGGAA

>P378

GACGAGTCTTTTATTCTCCTGTCACAGGACTCGTGGTGGGTGGGTGGGTGGGTGTGTCCA

>P379

TTGAGGCCTGAAAGCCTTCTCGTCAGCCCACGAGAATGTGTGACTCGGTAGAGTGGTCGGGAGGGAGGGAGAAGG

>P380

AAGGAAGGAAGGAAGGAAGGAAGGAAGGAAGGAAGGAAGGAAGGAAGGAAAGAAAGAAAG

>P381

GGGCCCCGAAGTAGGTCTTGTCGTCCCGTCTCCGGTGTCCCAGATCTCCTAAGTTTTCGA

>P382

GAACCTTAACTTCTGAGGTTCCATCCTTATAACAGATGGGAGAAGTAAGACTGAGCCTGAGGGATGGGTCTGGGGCCATCAAGTGAGGTGAAAGTCTCAGAGTACCCCGACCTGAGTTCACCAGTCTCTTGGACACCAAATACTCCAACTGGGACTGGAGGGTCTGGGAAATGAGAGCCCTCTCATTCCCATTCCAGGAC

>P383

TTTCGATCCTTCACGGACTAGGGTTTCCCCTTCCTTCTCACGTCCCGTGTCTAGAGTCGTCTCGGTGGAGGGGACCCTTTTATGGGAACAGGAGTGAAGTTCATCCTATCTATCGGGTGTGAGGGGGAGAGACGA

>P384

AGCTTCTTGGAGGCGCTCATTTTTTTTGGGGGGGGGGCTCCTTTTTTCTTCTTGATGCTTTACAACTTGGAGGTGGGGTCTGCCCTTGGAAGGGAAGGCAGGGCTTTCTCTCCCATGCAAAGCAAGCCCCCAGGC

>P385

CTCTAAACAATCAGAGCTAGGAGTCCGTCGTCCCTAGTGCGTCTGTGTCTGTCTGTCTGTCTGTCCGTGTGTGGTCTGTGTATCTGTCAGTCTGTCTGTGTGGGGTCCGTGGGACCGGGAGGGTTCACCGACTTGAGCGCCTACCGACTT

>P386

GGACGGAAGGACGGAAGGACGGAAGGACGGAAGGACGGAAGAAGGGAGGGAGGGAGGGGGGGAGAGAGAGAGAAGAGGGAGGAAGGGAAGGAAGAGAGACTAGGAGTAAGGGACATAAGA

>P387

TAGTACTGTGTCCCGAGACTCACCAGCATGTACCGAAGGAGAACACGGTATATCTATAGAGAGAGAGAGAGAGAGAGAGGGAGAGGGAGGGGGGGAGAGAGAGAGAGTGTGGGTGTGTGT

>P388

AGGGGAGGGGAGGGGAGGGGAGAAAGGAAAGAAGGAAGAAAGAAGGAAAGAAAGAAAGAAAGAAAGAAAGAAAGGAAGGAAGGAAGGAAG

>P389

GAAAGTCCTTGATGTGCCAGGGAGGGGTGATACCCAGGGCATGAGGGAGGGACCTCCCCTTCTTAGAGGGGAAGAAGAAGGGGGAACTGTGTATGGTGGGGGAGACTAGGAGGAGAGGGGGAACTGATATTAGAAAGTTAAGTGAACAAATAAATAAATGAATGAAAAAAGTCTTTACTTTGGAGATATTTGGACAATGT

>P390

AAAAAGTGGGAATGGGTGGGTAGGGAATTGGGGGGGAGGGTATGGGGGACTTTTGGGATAGCATTGGAAATATAATTGAGAAAAATACGTAATAAAAAGTATTAGAAAAAAAGAGAAGACAACAGCATCACTAAG

>P391

TAATCCCAGCACTTGGGAAGCAGAGGCAGGCGGATTTCTGAGTTCCAGGCCAGCCTGGTCTACAGAGTGAATGCCAGAACAGCCAGGGCTACACAGAGAAACCCTGTCTGGGGGGGGGGGGCGGGGGACTGGTAGGGGGCTGTGTACATTTGACAGGAAAAAAGGAGAGTGCACACAGAGACTACAGAGAAGTAACAACA

>P392

GGAGACCAGGGGTCAGGTGGGCTGGGGGACAGGGACAGAGAAACAGAGAGAAAGGCAGAGTTAGCTAGGGGGACAGCAGCTGAGCTGTGTAGGGTGAGGGCAGGCTAGGTACCACATGCCCACTGGGAATGGAACAGGCGTCCATATGTGGGGACCCAGGTGGCACGGGCCTGGCCCTCCAGTCACCAGCCACTC

>P393

GTCAAACTGGAAGGTAGGATGGTAATGGGACCCAACAAATCCTTAGAGGTACCCTACGCCAGTCGGTTCTTAAGTTGACTTACATGGGGTTAGGGTGGTGATCTT

>P394

AATTTTTACACACACAAAGAAAAAAAAAGGTAAAAATAATCCATAAATCGAGTAAATGTAAAGGTTACGATATGGTTTTCAGGGGGTATGGCTGGGTGGGGGTGAGGGGATGGATGGGTGAGGGGGAAAAACTGGGACCGTAAGGGGACATGACCCCGTATATTT

>P395

TTAGGTAAGAAAAAAAGGTAAAATAAAAAAAAGAGAAAAAAATAATCCATAAAAGGAGCAAATGTAAAGGTTACGATAGGGTTTTCAGGGGGTATGGGTGGGGGGGGTTAGGGGATGGGTGGGTGAGGGGGAAAAACCGGGACCGCAAGGGGACATTCCCCGTAT

>P396

TTTGTCTGCTTAAACCACAATATTCAGATTTTATAAGTTCATTTTATTTTATCAATTTAAGGAAAAGGAAGGGGGGAGGAGGAAAGGAAGAAGGGAGGGGGAGAGGGAAGGAGGGACCTG

>P397

AAGAAGGGGAAAGAGAAGGAGGAGAGAGAAGAGGAAAAAGAAAAAGGGGGGAGAAGAAGGAGGAAGTAGTAGAGA

>P398

GTCTTGAGTAATATCTACTAGTATTCGGTAATACACCAACAACCCGTAACTTGAGTCCTGGAGACCTTCTCGTCGGTCACGAGAATTGGTGACTCGGTGGGGAGGTCTCGTGACTCGGTGGGGAGGTCAGGGGTCACCAGGTAGAACTAA

>P399

GAGGGTTCACGACCCTAATTTCCGCACGCGGTGGTGCGAGCCGACACGAATTAAAGATTTTCACGTGTCGGACGTACGAAGTGGGACGTCTGGGACACATCGTGGTGTGTGTGTCAGGGTGGGAGACCCGGGTCG

>P400

CTCAAAGGCACAAATTTCCGACTCATCGAGGGGGGTGGAACATTCAATTCTCTGAAGACGAATCAAAAGGCACCGGAGGAATCAGACACAAGAGGGTTAAAGTAGGAGAGGGTGGTCGGGGCCTCGGGGTGCGGAACCGAGTCCGACAAG

>P401

GGACCGAGTGAAAGAGGAAGACATATCAGACCCCATCGTCCGATCACGACGGGTGTAAGACCCTGTCAGAAGGTAGTGGTCACTTTGGAGGGACCTGTCGGGGGGAGGGGGGCTACATAG

>P402

TACATAAGAGGAGAAGGAGGAGGAGGAAGAAGAGAGGGAGATAGG

>P403

GAAGAAGAAGAAGAGGAAGAAGAAGAAGAAGAAGAAGAAAAGAGGAAGAGGAAGAGGAAGAGGAAGAGGAAGAGGAAGAGGAAGAGGAGGAGGAGGAGGAGGAGGAGGAGGAGGAGGAGG

>P404

ATGGGGAGGGAGGGAGAGAAAGGGAGGGAGGAGGAGGGGAAAGGAAGGAGGGAGAAAGAGAGGAGGGAGGGAGGGAGAGACAGACAGAGAGAGAGAGAGAGAGAGAGAGAGAGAGAGAGAGAGAGAGAGACTGAAGGTCCTGCAATCTCCTTTAAAGGTGAGTCCTCAGGGACCTGGGAGGAGGACATTTGCCACCCAGA

>P405

ACAATAGGGAGAGGGGATAAGAGGGAAGGGAGAGAGGAAGGGAGGGAGAGAAAGAGGGGAGGGAGGGAGGGAGGGAGAGAATAAAGAAATACAGGCAGCCCAGGACCTGAGAGCTGAGAGCCAGGTAACCAGAGGGCAAGGGACTGAGGA

>P406

GAAGCCAATAAGGTGGGGGTGGGGGTGGGGAGTTGTCTAAAAGGCTTGAAAAGCCATAGGGAAACAGTTTTCATTCGGTTTACATAGACACTTGCCAAGGAGATGCCATTTGTATGACAA

>P407

GAGACTGGCAACCAGCTAAGGTAGGGGTGGGGAGTCAGGAGAATAAGCAACACTTACACACTGGAACTGGGGCCTTAGAAATCCCTAGTT

>P408

GCAGAAAAGGCCAAGTGTGGGGGAGGGAGAAGGGAGACAGTACGGGTGTCTGGCATTTTAAAAATTCTTGTAAAATGGGACTGGAGAGAT

>P409

CCCAGGACCAAGCTGGAGAAGTGTAACTTGGGGTCATGGGTTAGGGCTGGGGCAGGGCTTGCTCTCTCAAATGCATTTGCTGAGTGAACCTGGGGTCCTGTACAGATGTTAGCCCAAGTGTGGTTTATATTAGAAGCCCATAAGCAATCT

>P410

GGCCTCTGTGAGGTTGGGGGAGTGGGAGGGGGGTCTCAGGCACTGCCAGGGATGGAGAGCCAAGAGTATCTCAGATGTGGAGCTCTGTCGGGCAGGCTCTGTCCCCATCCCCCTGGGAGGGAGGCCCTGTCTCAGCCACAGGGCCCAGAAGCCTTGGCTGGCTTTCCTCAGGGCTCTTCTGCCTCTCCCCAGGTT

>P411

ATAACCTACAAAAGAAATAACCTACAAAAGAAATAACCTACAAAAGAAAATAACCTACAAAAGAAATAAGGGGAGACCTTTGGGGGATAGGGTAGGAGGGAGGGAGACTAAGATATTCCCACGAGAGGGTGGGTGGATGAGTGAGGAAGGGAGGACGGGTCCGTA

>P412

GACGTGTATTCGTGAGCAAATGAAGCGTAGACTTGTTTGTGATAAATTCGGAAATTATAAAATTTTAATTAATAAATAAATGTAAAGTTTACAACGGGGGGAGGGTCGTGGGGGAGAATCTTAAGAAGTAGGATAAGGGGGAGGGGAAACAAGACTCTCCCACAAGGAGGTAGGGGGTTC

>P413

TCATCATCTTCCGGTTGTCGTCGTAGAAACCTTCAAAAAAAATAGAGTATTACAGTACCATCCTGAATAAGAGAGAGTGAAGAGAGAGACATAGAGAGAGACATAGAGAGGAGAGAGAGAGAGAGAGAGAGACAGAGACAGAGACAGAGAGAGAGGGAGGAATTA

>P414

TGAACTGACATGGCAGCATTTCTTTCTTTTTTGGGGGGGGGGGATTTCAAAATTTATTTTTAAGCAAAGCATTATATGGCCTTAATCGTGCATTGGCAGAAATCTTAATAATTGATGCAAATTGAAGTTATACTT

>P415

GGAGTGGATGAGTTGGGGACCAGGGGAAGGGGGGAGGGCATAGGGGATTTTCGGAGAGGAAACTAGGAAAGGGGATAGCATTTGAAATGTAAATGAAGAAAATATCTAATAAAAAAAAAG

>P416

CACTAGACGTCGGAAGGGTTAAGATTGAGGACAGAGACTCAGAAGAAAGGCCGAAAGGGTCCTAGTAGGAGTGAGGGAGGAGTCTCCTGACAAGGTGAAGGGTGG

>P417

GCGAAACTAACCGTAGAAGGGTAAAGGAGGGCGAGGAACGAAGACCAGAAGTGAGAATATTGAAGACAAAGAGATAACCGAAGGGGTACAACAAGGGGTGGTGGTGGTGGTGATGGTGGT

>P418

CAGCCCAGAGACCTTCAGTCCACTGAAGTAAATCGAGGGTCGAGTGAGGTCTTTGGGATGTTATGGTTCCAGAAATAGAAGGAATTTTGGGAGGTCACTTGACTCGGGGAAGTGGGTCGGGACAACAACCCTTCT

>P419

TTGTTTTTTGTTTTGTTTTGTTTTTTTTCCTGTCCTTGGATCTTTTTCTTGATACGTGAAAGGGTGGAGAGGTGGGTGGGGAAGGACGAGGCGGGGAGTGGAAGGAGGTCGAGGCGGGGA

>P420*

CGGTGAGATGCACTGAAAAATGCACTAAAGACTCGTAACTTAAATCTAAGAGTACAAACTTACCCTTCCTCAAGTAACACAATTGATGGGGGGGTGGGATCGACAGATTCCGAACAAGAT

>P421

TGAACAGGCTACGCGACGGCCGTCAAAACCAGGTGGCGTGGACCTACCCGTCAGTGGTGTGACTCTGACTTCCGGGACGACACGTCCGCAGGGGAGGGACGGGGTACGAGAGGTCCCATG

>P422

TTAGTTAAACATAAACCCGTCCTTTCTTTATCCTTTCTTTGTCTATCTTTCATTCATCCTCCTTGTTGTTATACTTGATTGGACATGGGGGTGGGGTGGGGGAGTCTCGAGCACAGAGAT

>P423

GTCTCCCGTGTTTCATAGGTTTAATGATTTTAGTCTTTCCTTTCCCCTCTGTACTGTAGTCTGTGACTTCTTTGGGGGGGGGGTTTTTTTTAGTAGTCTAGGATGATGTTTTCGGTTATG

>P424

ATAGTGAGGGAGCAAGAATAGAACTGAACAGAGGGAGAGAGAATATCAAGAGTAGCTAGATTATAAGAAGGAGGAGTAGCTGGGGAGAGAAGCCCAGGAAGCGAGCATGGACTTTGAAAT

>P425

GGTACACCCACGGGGTGGGGGTGGGGTGGACTGGAGATTTGAGGG

>P426

CGGTAATGTTCCCCGCGGGGGGACGATTGAATAATTATTTTTACATCACTTGTATTCGAGATACACTCCTCGATCCGATCCGTTACCTGTTTTGGCCGTTCAGAAGGGGTGGGGAGGTCCTTTACAGCGAGGGGGTCATTTTGTATTCGT

>P427*

CCAAGTGTATATCGGGAAGAGATTGTAACTTTCGACAGGTTTCTCGACATTGTAATCTTTGGAGTCTCTAGAGAGAGAGAAAGAAAGAGAGAGAGAGAGAGAGAGAGAGAGAGAGAGAGAGAGAGAGAGAGAGAGAGAGGGAGAGAGAGGGAGAGAGAGAGAGAGAGAGAGAGAGTGTGTGTGTGTGTGTGTGTGTGTCT

>P428

TGGGGACAAACTAAGTAAAGCGAGGGTCGTTTGTTACGATGTCCCTTTACTCGCTTGTCAAGAGAGCGCCTGGAGGGTGGAAAGGGGTTGTTGAGGTGAAGGACAGAGGAGGTGAGACGA

>P429

CTTTTGAAAGGAAAAGGTGGGACTGGGGAGAGAAGGGAAGGCTGGGAAGGAGGAGGAATTCAGGGACCTACAACTTTTCTCCTTGTGTTTTACCGCCATTTTCAGGAGCAGGGCAAAATATCTGTGGTTTCCACA

>P430

GTTGGTTCGGTTTACCTTTTTCTTCTGTTCTGACACAAACTTATATTTGGTACTCGGGAAGCTTAGGTCCTTACGTACGACACTCATGGTGTGAGAAAACTAGTTCTTATGGGGGGGGGGGCACCTGTGTGAAATCAACGTCACCACTAG

>P431*

GTGTGTGTGTGTGTGTGTGAGAGAGAGAGAGAGAGAGAGAGAGAGAGAGAGAGAGAGAGATACTTATATTGACAGGATTTCTACAAAACTGAAACATTAACATTGGTAATAATTAGAAGAAATTGCTAAATCTAC

>P432

TGTATCAGTAATGTTTTGTAGAAGGAACGAAACCTGTGTGTTGACAACACTCCGATGTGTGTATATGTTTGTATGTATGTGTGTGTGTGTGTGTGTGTGTGTGTGTGGGGTGGGACGGGTATCTGTAGTGTTTTGTGGAACGTAAGTAAA

>P433

AGCTTCTTGGAGGCGCTCATTTTTTTTGGGGGGGGGGCTCCTTTTTTCTTCTTGATGCTTTACAACTTGGAGGTGGGGTCTGCCCTTGGAAGGGAAGGCAGGGCTTTCTCTCCCATGCAAAGCAAGCCCCCAGGC

>P434

CCATTGCTTGAGTGAGAGTTTTGGGCTGGGAATCTGGGTGGTGGTGGTGGTGGGGTATAACCGGAAGAAACTCAGAAATTTTTCAAGCATAGAAAATTCCGTTATAAGTCGGGCCTGGGGGAACACTTCTTAAAT

>P435

AGAAGAAGGAGGATGAGGAGGAGGAGGAGGAGGAGGAGGGGGGGCAGGGGAATGGGGAGA

>P436

TCCAAGGTCGGGATCCAGTAGTGGATACAGGACTGGTGTGGGACTATGGTGGTTCAAGGACGAAGGACCATATCGACGGTTTTCTTTCTT

>P437

GGGTCTTCGGAACCTCCCTCTTATAAAGGAATGGAAAAAGTTGAAGATCTTTACCGGTAACAGAAAGGGAGAAAGAGTGTAGTAGGGTGGGAGAAAGAAGACAGTAGTGTAAATAATTAGACAGTAGTGTAAAAA

>P438

ATATATAAAATTTCTAATAATAAAAATAATCTACACAATAAATAAATGTAAAGTTTACGATAGGGGAAAGGACCAAAGGGGAGCTTTTGGGGGTAGGGGAGGGAAGAGGGAAGCGAGTGGTTGGGTGTGTGAGGA

>P439

GTAGGGGAATCGGGGGTGGGGAGGCAAAAGTGGGTAGGTGGATGGAGGAACACCCTTATAGAAGCAGGGGGAAGGGGGATGAGATAGGCAGTTACTGGGAGGGGGTGAAACCAGGTAAGGGGATAACACTTGAAATGTAAATAAATAAATAAATAAATAAATAAATAAATAAATAAATACAATTTAAAAATTAAG

>P440

GTCTTAAGTAAAAATAGAAACTACTTTTCCTTGAGGTGTTTGAACTTACGTCATAAGATCAAAGTATAGACAACGACATTGTTTATAGGACTGGAGGGTAGGGGGTGGATGGGGAGGGGGGGTGGGGGTGGGGGGGTTTTTTTTTGTGGAAACCCTTATTTCCGA

>P441

TTTGTTTTGGTTTTTTTTTAGGGGGGGGGAAAAAAAATAACAAAG

>P442

ATTGTTAATATACTGATAAAAGAAAAAATAATCTATAAAAGAAGTAAATGTAAAGTTTACGATAGGGTTTTCAAGGGATATGGGGAGGGACGGGACGAGGGGTTTTGATAAAAGTATTGA

>P443*

ACACAGAAAAGGGGATTTGGGGCTTCCTAAGAAGTCTAGTAACAC

>P444

AGAGGACCGGAGTGTCGTCGACCAGGGTGTCACCTGGTACAAGAAGAGACACCCGAAGGAAGGACCCATGGGGGGGGGGGTCATGGAGGGTGGAGACTCATGGAGGATGGGGACCCATGGAGGGTGGGGACTCAT

>P445

CTTTCGTTTATCCCTCAATAGAATTAATTTAGTAAACTTCATCCTTCTGGGTGAAAAATTTAAAATTAGAAAAAATTTGAGATATGAAATAGGGAAAGGGTCAGGTGGGAGACTGACAAG

>P446

GAGAAACAACAATTTAGAAAAAAATAACCTATAAAAGAAATAAATGTAAAGTTTACAACAGGGGAAAGGTTCAAAGGAGGGGGTCAGGGTCTTTGGGGGATAGGAGAGGGGGATGGGGACGAAGATACTTCACAA

>P447

TGTTTTAGAATGGGAGACGACTGCGAGAAGGAGGATGATATGGAGTTCTTCTTGGTTGTTCTTTCTTCTGGGACGACCCTGACCTGGGACCATGGGTGGGGTAGGTCTCGGGCATAAAGAATTTAAAGTAAAGAT

>P448

GCCTCAAGAAGGAGAACAAGATGACATGGGCGCCCAAGAACAAGGGAGGGGAGGAGAGGAAAGCAGACAGTGGAGAAGACTCTCTGGGATGCCTGAATGGTGACACCAAAGGTACAAACAAGTCACCAGCCCACCTGGTGGGCCTCCTTA

>P449

GGGGAATGCCAGGTCCAAGAAGTGGGAGTGGGTAGGTAGGGGAGTGGGAGGGGGAGGGTATGGGGGACTTTAGGGATAGCATTGGAAATGTAAATGAAGAAAATACCTAATTAAAATAAAATAAAATAAAAAATTTCAAAAAAAAAACATTACATTTTGACTAAATATTTCCTGGGTTTGGGGTTGGTTTGGGAGGCAAA

>P450

GGGATGGGAGATGGGGGAGGAAGAATTCTGTGAGGGGGGACTGGAATGGGGCAACACATGAGTTGTAAATGAATAAAATGATAATAAAATTATTTTTATATGTATGGTTGTTTTGCCTGGTGCCTGTAGAATCCA

>P451

TCCGGAGAAATGAAGACATCCTCTTTGTTACAAGGGTTTCATTCGATGATTTAATGGACCTAATCACAGAAAGGGGTGGGGGGTGGGGGTGGGGATACAGAGACAGACCTTAAAATTTATCTTTTCCTTTGAGAT

>P452

TCTGTGGAGCCAAGAGGGAAGGGTGTGGGAAAGGCTGGGTTGAGACACTCCAAGTGTAGCAAGGCTGGGTTGAGACACTCCAAGTGTAGCAGGTGAAGCATTGGAACCCAGAGATGAGGCTTGTAGATAGATCTT

>P453

CCTCTTCATATGCTACAAAACCTTGTATTTTGTTGGGGGAGGGGGGCAATTGAGCCACCAGCATCACCTGTTCTCCATTTGTATGCGTTGAAGTGAGTGTCCAAAGGTGCCATGCCAGTGTTAGACCCAGGGAAAAGTCTTATTTGGTGC

>P454

ACACACGTATCAGTCTCTCGTTTCCCTCTATTGACGGCACGAGTCGAACGAAATAAGAGGGAAGATTTTTAGGGGGGGGGAGAGAGAGGACATAAACACGACTCT

>P455

AGGAAGAAGAGAGGAAGAGAGGAGGAATAAAGTGGAAGAGGAAAA

>P456

AAACCGCCCCACACCGTACACGAAACTACATACCATGTTGTGTACATTTACGACACATTTATGGGGGGTGGGGGACGGTTCCTCCCTAGATCTTCTCCTAAGATT

>P457

TTGTTCCTTTTATGTCCTCTTAGTTTTGTTTTGATAAATAAATAAATAAATAAATAAATTTTTAGAAAAATAACTAAGAAACACTCAAAGTGTAGTACGTGGGGTCAGGGAGAAGAGAGGGGTAGACGAGTATAGGTGGGGAGTGGGAAC

>P458

TCTAGCGAGAAATGAAAAAAAAGTAAAATAAATAAAAAATAATCCATAAAAGAAGTAAATGTAAAGTTTACGATATGGTTTTCAGGGGGTATGGGAGGAGGGGTGAAGGGAGGGGGAGGTGAGGGTAAAGAAGCG

>P459

CCTGTGCTCACACAGTCTGAGGGGGTGGGTGGTGGCGGGTGCTGCTCCCAACCTCATTGTCTGTCCCTGTTAGCCTGGGAGGAAGGGAAGTGTCCCAGGTCTTCCCCTGCTCACAGCCAA

>P460

TCCGAAGGGAGGGTCACGGAGGACGGAGAGAGTGAGTTGAGAACTACGGGGGCCATAGAGAATAATAGAGAGTGAGAGGAGTTGTAGAGACGTAATCTAAAGGTCTCTGAACACGTAGACGGAGTCCTTGGGTCTCGAACGAGGGAGACAAGGTCCGAGGGGCGGGAAGGGGTACGTAGCATATCTCGCCGGGGA

>P461*

ATACACACACAATATGATTTTTTAAAAGCA

>P462

CAGGGAGTCTAGGACCTCGGTAGGGATGGGTGGGAGGCATGGTGAGGGAACAAAGAGAGGGCAGGAAGGAGCATATGAAAACCTGACACAGATAACGTACATCTCTCCCTGGTGTAAATCAGGTGCCTGCATTGA

>P463

GGCGGACCATCCGCCCGAGGGTCTCACACGTACAACAATGTCACCTGGACTCCCCTTCTCTCTGTGACTCAGTTCCGGTCCTCTTGGGTTCTCGGGGCGGGGGAGGGTCTTCCGTCTCGAGTGAGTGGACGTACC

>P464

AGTGCCACCACGAGAACGGAGAGACGCGTGTCTCATAAGCTTAGCACCCACCGTCCGGACGCGGGAGTGTTAGTCTGAACCCCTAAAAGGTGGGGTGTGGAGGGGACGGGGCCCACTTCC

>P465

AGGAAGGAAGGAGGAGGAGGAGGAGGAAGGAGGAGAAGGAGGAGGAGGAGGAGGACGAGG

>P466

AAGGACCGTAGAAATGAGGAGACCCACTGGTGTAGACAGGTCCCGAAATACGGGGTAAGTGGGGTTCTTGAGGTCTTGCGAGGGGAGATCAGAGGGTGTCAGGAGAGGTAGTCGTCATCGATGTAGGTTCCACGGAGTTTAAAGTCTGTGGGGGTTTCCCTGAGTCCAACCGAAACAGGAATTGGGGCGGTAATAGGTAT

>P467

ATCTCAGTGTTAGAATATCGTTTTGAAGAGACCGACTGAGACTTCCTGTTGCGTGCGAGACAGAAACACACGGAACATATTGGAACTAAGGAACAGAAGGGGAGGGGGTGGGGAGCTCTTCACCCTGACAAGACAAGACAAGACAAAGTA

>P468

GGGTGAATGGTCAAGTCTTCAAGACTGAAGGTCGAGGAGATGTCACGATCGGGGAAAAACCGGGAAGGAGGGGTGGAGGTAGGAAGTCGGATCACCTCAAAGAGT

>P469

TCCCTCTGTCGTTCTCCTTCCAACTTTCAGTGACGGGACCTTCGACTGGTACTTCGAGGTATCGGGGAGGTCGAGGTGGGAAGGAGGGAGTTGGGGAGGAGGGGGACGAGGAGGTGGGAATGGGATGAAGACGTA

>P470

GGTAAGTTCGTATGAGGTCTTTAAGATTCACCGTGGGACCGAATTCCGGGAGACTCGTCCCCTGGAACCGTGAGCCATCGGGTACGGTCGAGACTGTCACGGCTCAGGTGGTGGGATTAGGGAGAGGTTCAAACC

>P471

CAGATTCACGAACACTCACAGGGTTTGGTCTGTAAGTTCAGTGGGTGGGTCTCGGGGTCC

>P472

GTGAGTTGCACTCTGGTAGGGATGTTGGTGTCGGGAGGGGCTCTGCATGTGAAGTCAGGGAGATGGAAAACCTCTGCAGCTTCCTCTCGATTTTGTGGTGAACCTGAAACCTCTCTGAAAAAGTCTTTGGGAAAA

>P473

TAGTGTGGGACACCCCATACCCCATCCCCACTCCTACTGTTAATAGTCCGTCGGTAAGAGACGAGGTCGACCTCGGATGGTAGGGGAGTGGGGTGAGACGGTCGGTACAAGTGTCGTGGCAGGACTGTGTTGAAAGGGTACTCAGATGTCCGACTTTAGGGTAACGAGAATACCGGGTAGGTCTAAGGTCGGTAGAGAAG

>P474

ACCTAGGTGCAGGAAGGCAGTGCTACAGGATGGGGGTGGGGCACTGTGGGTGGATGGGAAGGCAGTTGAGGAAGCTACAGAGTCAGAGTTGAGAAACTCTCCAAAGAGATCAGGCTTGGAGCAGGGCTGGGTGTCTGAGTTGGATGACAG

>P475

GTAGGAATTGGAGGAGAAGTAGGAGATTATTAAGAAGAAGAAGAAGAAGAAGAAGAAGAAGAAGAAGAAGAAGAAGAAGAAGAAGAAGAAGAAGAAGAAGAAGAAGAAGAAGAAGTAGTAGTAACAGTAGTAGTA

>P476

ACATATAATAAGTACCACCTAATTAAAAATGTAAAAAATTAATCCATAAAAGAAGTAAATATAAAGTTTACGATAGCCTTTTCAGAGGGTATGGGAGGGGGGAGGTGGGGGGATCGGTGCGTGAGGGTGAAGAACCGGGACCACAAGGGG

>P477

TCTTCGATGCGGGAGGGGGAATAGGGAAGAAGACCGGATCCTCGAAGAATCTCTGTTATTTAAGAGGGGGTTCCGTACAGACGAGGTAGGGGTTTCGGACGTCGGGGGTAGTGTCCTATAAACCAGTAGGGTGGAGGAGGACGTGTGGTATTGGACCCTCTGGTTCAGCCGGGAGAAGGGGTAAACTACGATTGACGGAC

>P478

CCGTCGAGGAAGGGGTCCCAGTGAATCCGACATCAACTCCCTCGGACGTGTCGGATTTGAGATCGGGAGAAGTACCTATTGACTGTCTCTTGACTACCTAGTGGCGGGGGGTGGGGGGCTGAAAGAATTACATAA

>P479

GAACATCAGGGCTTGCTGTGGTAGGGTTTCTGGGCTCTAGGGGAGACATATCGTCTTGTCTCTTACTGATAGTGTTTTTGTTTTTGTTTTTTTTATTTTATTTATTATATGTAAGTACACTGTAGTTGTCTTCAG

>P480

TTGTTACCATAAACTGAGTAATTTAAGACTAGCATCACACAAATGTGGGGGTGGAAGGGGTGGGGGTGGGAGTGGTCGTCGTGGTGGGGGAGGGGGACGTCACCG

>P481

TAGATTTAAGCAGGGGCATTGGCAGGGAAGGAGAAGGGGCTGGGAGGCAGACACGGCTAACACCCATGTGGCAAGCACCCAGGAGAGGTCCTGGTTACTCCAGAGAGAGCAAGCTGTCAGCCTCTTTTCCTATGA

>P482

TCCCGCGCGCGGTGTGTGGGTGGCGGGGGGGGGGGGGGGTAGGGCAGAGGAAGATAACTATTGGCTTGACATTCACAATACTCTGCCCGGTAAATTATGTTCGGTGGTCACGAGGGATCGGTGGCTTCTGTGGGTGGGGGTGTGTCACCTTCTTTATTAGCACTGGCTCGCGGAGCCTGGAACTGGCACCCCAGT

>P483

TTCCTAGCGTCGTCCCTTCTGAAAAAGAGAAGGTCGGGGAGGTCATGTGAACCGGGAACGAGGGTTAGTCTGACCTAAGACGAGTAGGGCCCAAGGTAGCCTTGGGAGGGAGTACACTGGGAGGGAGGGGTGTGGAGACGGACCACGCAT

>P484

GTACAGGGGAACACCAGGGCCAAGAAGTGGGAATGGGTGGGTAGGAGAGCAGGGGGAAAGGGGAAGGGTATGGGGGACTTTTGGGATAGCACTGGAAATGTAAATGAAGAAAATACCAAATAAATAAATAAATAA

>P485

CAGGTGTGGGGATGGGGTGGGGGTAGGGTCTTCTCTAGTATCCTC

>P486

TTTTTAGAAAAAAAATAACATAAAAATAAAAATAAAAAAATAAAAAAAAAGTAAAAAATAATCCATAAATCGAGTAAATGTAAAGGTTACGATATGGTTTTCAGGGGGTATCGGTGGGTGGGGGTGAGGGAATGGGTGGGTGAGGGGGAAAAACCGGGACCGCAA

>P487

TGTCCCTGTAACATCCAGGACCCTAGGTCCCCTAAGGTCTTCCATACACAGGAGGAACGAGATCACGAACGAGAGAAGAAGGACAGGGGACTGGGGAGGGACGTGGAGAGGAGTGGAAGGGTGACGTGGGATGGGGGTCTGGACCTCGTTCTTGGGTAGGGAGAG

>P488

CTGAGTGAACTAGTGGACGAGAAGGGCAAACCGGACGAGAAGACGGACGAGTCGAAGACAGTGTAGGACCCTAGTTTAATCCCAGTCGGTTCGGGGTCACGACAGTTTCCTGACGGGACG

>P489

AAACCCCCCCCGTCCCTATTCTCAACGGAACCAGTACCACAGAGAAGTATCGTGATCTTGTCCCTGATTCTCTTGAGGGTTGAAGTACGACTGCTGGGGTGGGGTCGGGACGGGAGGGAACCATCCAGAGAGGAGAGGCAGGGTCCGTTC

>P490*

CCACTTGTTTTGGGTCCAGTGAGGTCTATCCCACGGTTGTTGTCT

>P491

TGTAGGTTATGTATCGACTGGAGACAGGGAATAGGTGAGTGGGGTTGAGGAGGGTTACGAAAAAGACACGAAGAGAGAGCTGGTAACAAATCGTCGACAAGGGTGAGTGTAAAGGGAAGTACTTGGGGTACCTGTGAATGGGTTCTCCTTTAGTGCCCTGTAAGGAGAGAAACTGGATGGTGGGCTGGGAGGGGGAGGGA

>P492*

CACACATGAAAAGAGAAGAGAAGAGAAGAGAAGAGAAGAGAAGAGAAGAGAAGAGAAGAGAAGAGAAGAGAAGAG

>P493

CAGTTCAGTAGCTAGAGATTTCAACAAGAAAGAAAGAAAGAAAGAAAGAAAGAAAGAAAGAAAGAAAGAAAGAAAGAAAAAGAAGGAAGGAAGGAAGGAAGGAAGGAAGGAAGGAAGGAAGGAAAGTAAGAAAGT

>P494

ATGTCCTCAAAATAAGGACATATTAAATACAGAATTGAGTTCTTTTTCAAACTATAAAAGGATGATTAAATAAATAATTAAGTAAAACGTAGGTCTAGGGACGGGGGGGGGGAAGGACCAGGGAGTGTGTGTCAGGGAAAGGGGTATTGGAGGAGGGAAAGAGGAGACTCTCTCACCTCTAGAGGGTATTCATGGGGGGA

>P495

ACACACACTCACACACAAAGATAAAAATTACTTGTAGTAAAATGGAGGGGATTGAGAAAGGCCCGTGAAAACAAAAGGAAACGTGGGGGGGGGGGTGGTAGGGCAGGGAGTACGAATTAAATCGATGATCACATC

>P496

AGGACACGTCTACTATTGTCGTTGGTCTCGAGGTATCGTCTGCGGAGGGGGTAGTTCGTTCTCTTGTCGTCGTTGAGGTCGGGACGGGGTCTCTGGTTGGGTGGG

>P497*

CCCCAGCAGGAGAGAGAGCAAGAGAAAGAGAGAGAGAGAGAGAGAGAGAGAGAGAGAGAGAGAGAGAGAGAGAGAGAGAGAGAGAGAGACCTCAAGGCAAGCTGCATATCTGAAACAGCTGAATTACCAAGCTCTGGTTTCAAGCTAGAG

>P498

GCAAGTAGAAAGGGGTGGGGTGGGGAGGAATAACTATGAGGTAGATGAGGCTGAGAAAACAAACAGGTTTCATACCTGGCAAAACAGCAACTTAAAAGCTTCAGATCTGTCCTCCCTGAG

>P499

TTATATCAATTTTAACGATAAAAAATGTTAAAAAAAAATCTATAAAAGAAGGAAATGTTAAGTTTACGATAGGGTTTTCAGGGTATATGGGAGGACACGGGACGAGGGGATGGGTGGGTGAGGGATAAGAACCGGGACCGAAAGGAGATA

>P500*

GTGAGTATAATTCCCGTCGTACCTTCTTGTTTACACTTGTGAGAA

>P501

TGGGTGAGCAGAGAGGAGGGTAGAAGTGAGCTCAGGCTGAGGGGAAGGTGAACTGGTTCGGACCCTACAAGAATTAATAGAAGCAAGTAT

>P502

TGGCAGTGGCGGGAGCTCTGTCTGGGGACTGTCTGAAGAGTCCTG

>P503

AATAAATGAACGTAAAGTTTATAATAGGGGAAAGGACCAAAGGGGAGGTCTTGAAGGGTAGGGTAGGGGAGAGGGGGACTAAGATACTCT

>P504

AGAGACTTGGGGCTCCAGGGAGTGGGGAGGTCTGGTAGAGTTGGAGAGTGGGGACATCCTCTAGGAGACAGGAGGAGGAGGTATGGGATGTGGAACAGTTAGAAGGTGGACCAAGAATATCAGGGCTTGGAGCTTCCACTTGAGCTGGACCCTAATTTGGGCCTG

>P505

GGAGGAGCAGGAGGAGCAGGAGGAGGAGGAGGAGCAGGAGGAGGAGAAGGAGCAGGAGGAGGAGGAGGAGCAGGAGGAAGAGGAGGAGGAGCAGGAGCAGCAGGAGAAGGAGGAGGAGGAGGAGGAGGATAAGGAGCAGGAGGAGGAGGGGGAGGAGGAGGAGCAGGAGCAGGAGGAGGAGGAGCAGGAGCAGGAGGAGG

>P506

GGAATGCTTTGGCCAGGAAGCTGGAGTGGGTGGGTTGGGAAGCAGGAGGAGCGGGGAGGGGATAGGGGATTTTCAGAGGGGAAACTAGAAAAGAGGATAGCATTTGAAATGTAAATGAAGAAAATATCTAATAAAAAATATATTAAAAAA

>P507

AATGGGGGACCCGGTGTCGTGTCCTCGACACTTCCCCGAGTCTTCCTTTCACACTCCATTCTCCGACTTTCACCCTCAGTTTCAGAGGCGGGAATGGGTCGTGGGTCCTACAGTCTTCGG

>P508

CACGCTTATACGGGGTGGGAGGGAGGGGGATCGCGGGGGCCGGGGAGAGGGAGAGGAAGAGAAAGGAAGAGGGGGTGGGGAGAGAAATAGAGGAACCTATGTATTTTCATGCCGTTTAAAGAAGAAATGGGCAGTCCTGTTGAGTTCTGCCTGGATACGGGAGGGATGCTCTACCAGTGC

>P509

TGTAACACCATGCCTCATCTTATTCAGTGCTGGGGGGGGGGGGGTAGGGGGGGCTGGGCTGTAACCCAGGCCTCTGTGCACGCTACCTAAAGATTCTACCGAGTAAGATAAATCCCAAGCCCTACACATTTATTTTCATAAGATTAATATAATTTTATGATGTTA

>P510

TCATTTTGTTACCCTCTACCAGGGGGTTGTCGGGACGGGACTGATCTGAGGCGAGAAGTCCTTTGAACGTTCCCT

>P511

CTTCTTGTTGTTGGGGGCGTTGTTTTGTTTGTTTGGTTGGTTTCTGTTTTTTAGGACAGGATCTCTCTGCCTAGGGTTAAAGGCATGTGCCACCACACAGTGCCTAAAAGTTATTTTTCC

>P512

AATTTACATCGTAAAAAAATGAATAAATTTTAATTTTATATCAAAATAGTAAAGGGGGAAAACGGGGAGGTTGGGGAAGGTACAAAGAAGGGAGGTTGAAGGGTT

>P513

GAACATACAAACAAAGAAGTACGACAGACAGGAGGTAAGTCTTAAAATCATGATACTCTCAGATTGTGTACCTTTATTTGGAATAAATAGAAAAGGAGGGAGGGGAAATGAAAGAGATAGAGGGGAGGGAGGAAAGGAAAGTAGGGAACGTATAACATATCATGG

>P514*

AAGAGGCAGAGAAGGCGTATTACGAGAGAGAGAGAGAGAGAGAGAGAGAGAGAGAGAGAGAGAGAAGAGGCAGAAGAAAGAGAAGACAAGAGGGATCATAGACAAGAGAGAAATTTGACTAGAAACTTGGACACAGTGGTAGGAGAGAAA

>P515

CGTAGTAGTCTCTGAGAGTCTAGAAGGAGAATGTAGGTGGACTCCCGTGTCGGGTTGGGAAGTACGAACGTGGACGAGTGGGGGGAGGGGGTGGGGTGGGGGTGGAGGTGTACTGTGCAC

>P516

AGCCGAGGCACCGGGCGGCGGCGGCGGCGGCGGTGACCGCGGCGCGCGCCATGTCGTTCAGTGAGATGAACCGCAGGACGCTGGCGTTCC

>P517

ATACCGTAAAAGTTTCTCAGACCCAAACAATCAGGAGGTTAGTGGAGAAGAAGAGGTAGGGAGAGGAAGGAAGGGTCACAGGAAAGGAGGTGGGAGGGGAGGTAGGAGGGGTCACAGGAGAGGACATAGGAGGGGAGACAGGAGGGGAGGTAGGAGGGGAGACAGGAAGGGAGGCAAGAGGGGACTTGGGAGGGGAGACA

>P518

GAGGGTGGAACAGTACTGAAGGGAGGGGGTTAGTGGGGGTCTGCTCAAGGACTAATTTTATGGCTGTCGTAACTGTCTGCGACCTGACCT

>P519

AAGATGGAGGGAGGGAACCGACCACTCTCATCTCTTCTCGAGTCGAGACCAAGGGAGGGAGGTGACCGACGGAGAGAGGGAGGGTAGACCGTCTTAGTCAGTCGG

>P520

TGGGATTTCATAACAAATATCTCATGCCTGGAGGTGTAGGTTTGAATCCTGACATTATTTCAGTTAGTGGTCCGTTACGCCAGCGTCCAG

>P521

TCTGGAGTGCTTTGAGTTTCTGGGAAGGGCAGGCCCAGGGGAGGGGAGCTGCCTGGTGGCTCATAAAGGACAGAGCATCCCTGCCTGGGATGAATGGGTGGCGGCCTCAGGCAGGGATGCTCTATGGTCCCTTTCAGCCTAAGTGCAACA

>P522

CCGACCGGAGTTTGAGTTTTTAGGTGGACGGAGACGAAGGATTTACGACCCTAATTTCCACACGCGGTGGAGATGGGCTGGGACGAAAAAAGGAAAAACCAAAAAGAAAGACCCCGTGACGTGAGCACCCGTGGGAGGGAGGGGTCTATGGGTGTGTCGTACTGA

>P523

TAAAATAGACATTTAAAAGTGAAATTTATATAAAAACTTATATCTAAGAAGATAGTATATTATACAGACCGGTGTCAAAGGGAAGGGAGGGGAGGAGGTTCTAGA

>P524

CAAAAAGTGAGAAATAATAAAATAAATGTAAAGTTTAAAACAGGGAGAAGGACCAGTGGGGAGACATTCGTGACGTGGGCTAAGGGGGAGGTGAAGTGGAGATTCTCCCACGAGGGGGTGGGTGGGTTGATGAGGGTGGAGTGGGGATCG

>P525

TCCCACAAGAGTCTGGTACCAGCTTCCTGGGGGTTGGGGGGTGGCAGATGAGGCTGACCACCAGAAAGGTCCTCTGAAAGTTTGGAGCTGCTGCTTCTGTCTTGGGAGAACTGAGAAGGT

>P526

GCACACCAGAAGCAGCCTTGAGGGAGCATGGTTGAGGGAGGGGCTTGTTGTCCCAGGGATCTGGGGACACTTCCACGGACATGAGGCTTGCAGCTGGAGAGTAGAGAAGGCCACATAGGCACACCCTCCAGCCTG

>P527

AAATAATGAAGAGAATTAATGACGAGGAACAAGCAGGAATAGAGTGGTTGGGGCTCAAACAGGGACTCACGGACTGAAACAAAGACCCCTGTGTGGGTGTCTCTCGTAGGGGGACGGGTGGGCACTGTGTGGTGTCTCTCGTAAGGGTACAGGTGGGGTCACTGTGTGGGTGTCTTTTGT

>P528

GGCTTACAGGCTGCTGGCAGGGATGGGCATTGGGCAAATACCCAAATGAATATGTCCTTGTCAACTCGAGGGCTCATCACACTCAGCCAATACTTAGTATGCACTGGTGTGTGACCGGTC

>P529

TTTTCTGATCTTCTTTGACTCAAGTCCTACAAGTCTGACCACCTAGGTATCGGTTTCCGGGGACTACCAATACGACTCCGATAGGGAAATTCTGGGGGGGAATCGGGAGACCAGACTTGAACACGATGTTAAAAC

>P530

CTGACGAGGATTTAGGAACCTGGTTTTAATAGATGTGACGTGGGGTGAAGGATGTTCACAGTCGAGAGAGCACCGTCGGGGGTGGGGGTGGGTCTCATCTCCCCTTCTTCGTCCCTTGTC

>P531

TCATCCATGTGCCAGGCAGGAAGGGAATGGTTGGAGGTGGGGGTGGGCGGGAGCTGAGTCCAGTTTCGTTCTGAGTGAGTGCTAGAAGAGAAAAGGCTTTTGTCTTCTTAGGGAGATTCAGTCACCGCTCTGTGGTGATCCTTGATAAAG

>P532

TGAAGACTACATCCAGATCCACAGGCAGCAGGGAGAGAGGGAAGAAGGGAGGGAGGGAGG

>P533

ATCTATATTCTGAAGGGTTGTAAGGGGGGGTGGGGTGGGGAAAAGGAAAGGAAAACAAAAACAAAAACGAAAAGA

>P534

GAGAACGGAATCTTCGAATGATAAAGATGGGGGGGGGGTGTGGAGAGAGCGCACAAAGACAGGGCATCATGAAAAGGACCACAGATCCTTCCCCCTAGATGTGGGCAAGGGGGTGAGACATGGAAACTTGAAGCT

>P535

AGGAATGCCAGGGCCAAGAAGTGGGAGTGGGTGGGTTGGGGAGCAGGGCAGGGGAAGGGTATAGAGGACTTTTTGGATAGCATTTGAAATGAAAATGAAGAAAATATCTAATAAAAAATTTAAAAAGGAATATAA

>P536

TCTTGAACTTCCTCCAGAGAGACGAGACACTATTTTTCAGTTTAATGTAAGAAGTTTGAGAGACCTCTGAATCTAGAAGTGGGGGGGGGGTTATGGGACATAGTCTCAAATATACCCTTT

>P537

GACCCCACTCTGGTCCTTGAGAAGGGGCTCGACTCCGCGGGATGGACGTCCGTCCTTCCCTGTCTCCTTTGGTGAGGGGGGCGGGGGATG

>P538*

TATATACGAGTACGAAAATGGTTTGAATGTAACCATTTAGTTGTC

>P539

ACCCCTTGTCGGTAGTGACGAGAAGGGACCCGAGAAAGTGACACAGTAGGTACCGGTGTCATTGGGGTGAGTGAGAGGGTGTGGATTGACCATTCCGAGAGAGGAGGGAAGAGGGACGGGTGAAGAAGGTAGAAGAGAGGATTGTCCGGACGAAAAAATCACAACGGGGAAGGACGGTGTGGGGAGTGAGTGGGGTTCAA

>P540

TATGAAGCTGTGAAAGGGTGGGGAAATGGGCATAGATCTTGTAGGAGCTACAAGGATGAATATGTTTAAAATACATTGTCAGAACTTCCTTTTGGGGGGGGGTTGGTTTTTCGAGACAGGGTTTCTCAGAACTTCTTGAATTCATTAAAATATATTGTATAAATCAGTCATATAACATTTTTAAAAATATATATT

>P541

ATGAGGAAAATATCTAATAAAAATATTTAAAAAAAGGAAAGAAAAAAGGGAGGGAGGGAG

>P542

GAACCCTCCGTCTCCGTCCACCTAAAGACTCAAGTTCCGGTCGGACCAGATGTTTCACTCAAGGTCCTGTCGGTCCCGATATGTCTCTTTGGGACAGAGTTTTTTGATTGGGAGGGGGGACGGGGGGGGAGGGGGGGTTTTTTCTTTGAG

>P543

GAGGGAAGGTCCAATTGGGAGATGATGAGGGGTTAGAGTAGACGGAATAACGGGTAGGGGAAACGGAGAACCTCC

>P544

GGGGGTGGGGGGGAGTCGGGGTCACAGTGGAGTGCTAGGGTGGAGCTGTGCTCAGCAGGGGGCAGCCTAAGCTACAAAAGGATGGATGGCTCTGGCCTCACCTAGGTTTACCTCTTGCTA

>P545*

CATTGACCTTTTGACGGATATATAAAAAAGTTCCGTAATATATGA

>P546

TGTGTGTGTGTGTGTTGTGGGGACAGGGTAGGGTAGGGGGGAAGGATGTAGGGAATGGAGGGAGGGAGGGAGGGA

>P547

TTTTTAAAGATTTTGTGTGATTTGGGGTGGGGGAGTAGGTGCCACATGAGTGTGGGTGCCTGCAGAGGCCAGAAGAGGCTTATTTCCCCT

>P548

GAGGGAGAGTAGGGTAAGCTCTCTCCATCCTATCCTCTCCCTGAGCCAGACCTAGAGACAGTCATTTTGGGGAAA

>P549

TACATCAACGACCTTTAACTTGAGTCCTGGAGACCTTCTCGTCGGTCACTAGAATTGGTGACTTGATAGAGAGGGAAAAACGTAGGGAGGGGAGAAAACTCGTAAAGTATGTACTTACGATATAAATACAACGGTGCGATGGGGAGGGGCTTGTAGGGGGATAGGGACTGGGGGGTGGGGAGTCGACATACTGGAGAAAC

>P550

GGGGGTGGGGGGTAGGGACGAGGACGGGGG

>P551

CCACGACTTTATAACGATAGAGGTAAAAGTGTCTCCGTGGGGATTGCTTGGGGAGACGGGGGGCCTCTTGTTGTCTACTACCAAACTCTT

>P552

GAGGAGAGGCATCCCGCACTGTGAAGGTACGGAGGTGTTAGACAAGTTGGACATCCCCTTTCGCCCAGTGGTGCGTCTGGCTTCAGGGTTCGGAGGGCGGGAACGAGGAGCAAGGGTGCACCGTCTCACCTAGAC

>P553

ATAAAAACAAACGAAAAGACCCACAGCTTTGTCCCAAAGACACTAATTAGATCCGTATATTGGTTGGAGTAGGGGGGGGGGAGGTGGAGA

>P554

AGGTAACACTGAGGGAAGTGAGGGTGGGAATCCAAGCAGGGCAGGGACCTGGTAGTAAGCAGTGAAGCAGAAGCCATGAATGTATGCTGCTTATTGACTTGATCTCCCCATGACTGGCTC

>P555

ATGAGAGAGAGAAAAGGGGTGGATCCAGAGGGGAGGGCGGGTGCGGAGGAACTAGGAGGAGCAGAAGGAGAGGACAGTGTAATGAGGTATATTGCATGAGATAAG

>P556

GTGGAACATTTTAAAAGAAGGAGGGGGAGGAGGAGGAGGAGGAGGAGGAGGAGAAGGAAGAAAAAGAAGAAGAAGAAGAAGAAGAAGAAG

>P557

AAAGAAGGGAAGAAGAATAAATAAAAAACAAAAAATAAAATAAAAAATAACATATAAAAAGATGAATGTAAAGTTTGCAATAGGGGAAAGGTCCAAAGGGGAGGTGTTATGGGGATAGGGTAGGAGGGGGGTTGGGACGAAGATACTCCCACGAGGAGATGGGTCGGTGGCCGAGGGTAGAGGAACGGGATCGTAAGGGG

>P558

CTAGGGACAGGTCTGGGATGTGGACGGTGTGTGGGGTGAAGGTACGATAGCCGAAATGGGGTGGGGTGTCCTGAGTTCCGTTGCAGAAAGACTTCCAGGTTCCGAAGGGTATGTAGAGTGGGGTTGTTGTTTCCGGGACGGGTACGTCAATCGTGGCGGAGGCAGGTGTCCGGGGCGGGGTTCGTTCTCTTTGGGGAGGG

>P559

GTAGAGGGTCTCTCCTTGATTCGTGTTCCTTCAAATTCGTCAAAGAGTCTCACCCCGCCGTCCGGAACTTTCAGTGGCCCGAGGCACTCGTGAGTGGAGGGCACGACGAGTCGTGTAGGGTTCCGGGGTGTCTGTGTCGATCGAGGGGAGATGGGTAGAGTTCGT

>P560

GTGTGTGTATGAGAGAGAGAGAGAGAGAGAGAGAGAGAGAGAGAGTTAGTTTATGTGATCCGTAGCAAATGTGCAGCTTGCTATTCATGTGGGTCCCCTAACAAGTGGGGGAGGAGCTGT

>P561

ACGATCGAGAAGGAAATGGGTCAAGGATCGAGACCCGAGAAGGGGACGGAGGAGAAAGAGAAGAAGGAAGGTGAAGGGGACGGGGAGGAAAAAAGAGGGGGGTGGGGGAACAAAAAACTCTATCCTAGAATGTCTGGACCGACCGGACCT

>P562

AAATAAAAAAATTTCTGTCTGAAAGTACGACGACTTGGACCTCGGAGGTTACACCGTTCTGGTCGACTGGTGGTCCGAAGTCCCCGGGTGGGAGGAGGTGGAGAG

>P563

AGACATTCCCATTCAAAAAAAAAGAGAATTAAAAAAGGGGGCGGGTGAAAGGAAGCCACCAGTTCTAAGCAACTTACAAATCCATCTGTGAAAGCTCCGTTAGTTCTCATGGCTGGGAAT

>P564

GGGCCTAGGTCTTCAGGGGAGGGGCGGCCGAGGGCCTGAGGGGCGGAATTTCCGTGTTCGAGGAAAACAGACCCGTGGGGGAGGGGGAGGGTCTCCGCCCCCGTGTCGACGGGTGAGGGGAGGGGCCCCGGGCCCAGCCCACTTCCTCCGGTCGCAGGGCGCGCG

>P565

TTGTTTTCTAGGGAAGGGCCTACATGGGGTTTGCCCTGGCATGTGGTGAAAACTGAGTCTTAAACTTTTCTTTTCTTTGAAGTTTTCTTG

>P566

TGAACCCGGGAGATCTTCGTGTCCTTTTCTTCTTGTCGAAAACGAGAATTCGATGTGGGGGGGGGGGGGCAATCA

>P567

GGGGGGGGGGGAGCAAGAGGAACTGCTAATGTTATTCATTTTGATTTTTTTTTTTTTTTTTTTTACCAAGTTACA

>P568

GTCGACGACCAACCCGGAGAGTCTCCTGTCGGTACGATCCGAGGACAGACATTCGTGTCATTACCACATTCCGGAACCAGGTGGGTGAGGTGGGTTGGGGGCGGA

>P569

ACAACATCAACACCTTGTACAAACCGAAGTCTACGGTCTTGTTTGTCAACTCCACGTATAAAATGTATCGTTTCGTCTGGACCGGAGGTCCACGATGGTCGTAGGGGGTCAGGGATGGACCGTATAGGATGGGGATTGGGACTTAAAAGGTCGGGACCCCGACCCGACAGGACGGGGGTC

>P570

GCTCTATGAATGTGGACATCGATGTGCAGGGCGTGGGTGGGGATGGGTTGGGGATTAGGGGTTGGGGGGGGGGTAGGGTAAGCAACTTGT

>P571

TTCGTTTGGAAAAGAGTGCAAGTCCACGAAGAAAAGTCCCATCAAATAGTGTCGTTGACCTTTCCCCGCTCCTGTGTAGACATATGAACTCGAGCGAGGGAGAAGGGGAACAAGAAGAGGGAGAGGGGCAGGGGAGAGAGACAAATCATTTCAAGTGGAGGGTCG

>P572

CAGAAAAAAAAAAGGGGGGGGGCTTCTATAAGGAAGGATACGTAGGCTTTTGCAGAGTTCAACCTGTTTGGGACCCACCCTAGCTGAGTTGCAGGATTAATTTAGGCAGAGTATTTTTGG

>P573

ATATGTGTCAAGTTGATAAAGAAGAAAGAGGGGGAGGGGGAAGGGGAAGGGGGAAGAAAGGGGAAGAAGGGGGAAGAAGGGGGAAGAAGAAGGGGGAAGGAAGGGGGAAGGGTGAAGGAGGAAAGGGGAAGGGGGAAGGGGGAAGGAGGA

>P574

AATAAACAACTCAAGAACCAACCAACCAAACCGATAAATTAGATAAGGTACAAGGATATTAGTCGTTACTCCAAATGTCTTTTTTTATGGGGAGGGAGGGAAACGATCTCAGAATGGTGTAACTTTTATCTCCAC

>P575

GAAGAGGAAGAGGAAGAGGAAGAGGAAGAGGAAGAAGAAGAAGAAGAAGAAGAAGAAGAAGAAGAAGAAGAAGAAGAAGAAGAAGAAGAAGGAGGAGAGGAGGAAGGAGAAGAAGAGGTTGAATTAGGAGTGCAC

>P576

ACATAGTGGGGGTACAGATAAGAAGTAACCAGAGGGGGTAACATGGGCGATGGCCACCCAGCTAGGGGTCTGCTCATCCCAGCTCTGGCCTCTGCCTGGCCTAATTCCACAGCAGGTGCCGTGTTCCCCTCTACAACATGCCCTGGGGGT

>P577

ACACACACACACACACACAGAGAGAGAGAGAGAGAGAGAGAGAGAGAGAGAACAGACAATTAAAATAAGAAAAAAAAAGGAGGTGCTTGGGTTGTCATCCCTCTGGTAGTTTACCCGACT

>P578

TCGGACAGATCCGGTCGTATGAGGTCACAAGATGTCTGTGCGGTCTTACCGAATACATCCCGACACCCGTCGACAGGTGGGGTGAGGGGGTGGGGTGGGTACCGACGGTGTCTTGAAAGATAGGAGGGGAACGACCCGTGGGACCCGTACCAAGGAGGGGTCGTCTGGGTCGGGTATGAG

>P579*

GAGCCCGTAGGACTG

>P580

GGGGGGGGTTCCTTTTTAAAAATCTTCTCATTTTTGGTTTTTTGTTTGTTTCTCACGTCTACTAAACGTGCTCTCAGATAATGTCACACATGTACATGTGTATAC

>P581

AAGACTGTGTGAGTTCTAGTGTCCACTCCGGTGTTGTAGAAAGGGTTGTGGGTAGTATTGACCCTGGGTGTTTCCTCGATACCTTTGTTTGGGTCAGTCTCCGTGTCCAAAGAAGGTCGAATGTGGACATGGACCTCGTTTGGGTCCCAAGACCGAGGGGTGGGTTGGGACATTGTGTGTCTTTTCCGAACTGAA

>P582

GGTTAGCAGCATTCAGGGATCCCTGGGCAGGGGTGGGGGTGGGGATGGGGAATCTGAAAG

>P583

GATCCGAGTGTGTAAGAAGTGTAAAGAAAAAACAAAATTAATCCATAAAAGGAGTAAATGTAAAGGTCACGATAGGGTTTTCAGGGGGAATGGGAGGGGAGGTGAGGGGATGGGTGGGTGAGGGTGAAAAACCGG

>P584*

CGAAAGGATTTAACCTAGTGGGTTCAGATGATCAAGAAACGATAATTTGTCCTACTGGTTTCCACAACACTTCAT

>P585*

GGAAGTCTCGGACTTTCGGGACCATTCCTAGTTTTCGAAGCCGTT

>P586

CAGAAGACGGACAGGCAGAGAGGAGGTTGTGAAAATGTAAGACCGGTCGTCGAATGCGAAGACCCTTATTACTAGGGGGGGGGGTAGAGGTGTGAGTTAGAAAAGGGAGATCAAACGAGTGATACCTCAACATCGGATGCCTTCCCTGAGGTCGGTCTAACTTTTATACCGGGACCGTCCGGAACGGGAAGAGGGGGGGT

>P587

CGAAGGAGGAGGAAGAGAAAGATGAAGGAGGAGGAGGAAGAGGGAGAAAGGAGGAGCAAGAAAGGGAATGGAAAGGAAGGGAATGGAACAGATGGAGAGGAGAGGAGAGGAGGGGAGGGG

>P588

GTATCACACATGGGATGGTGGTGTCCTTGTGTGTACGTATGTCACACGTGGGGTGGTATC

>P589

ATTTATTTGATTTTTGTTACAGTTCACCCTTCTATAATGGACTGAAGGAGACTAGGATCAAGAGATTTTTTTTAGGGAGTCAAAATAGTCACTCTTCTCATTTGAACGTTGGTATCAGAGGGGGGGGGCGGGTAA

>P590

TGTGACGGCTGTGGGGGAGGGAGAGGGAAAGCAGTTGGGCAGTGGACTTCCAGATCCTCCCCCTCCCCCTCCCCCCCCCTCCCCCCCCCGTTGACGATTAGAGGGACATGGTCTTTCTTGGTAAGTTGGATGTGT

>P591

CAACCATCCATCCCCCATCCATTCCGATAAGACTTTAGTCCTTGGTCTCTCCCCACCACTTACGAACTCTCGAGAACGAGAGAGAGAGAGAGAGAGAGAGAGAGAGAGAGAGAGAGAGAAAGAGTATGACCCTTA

>P592

AAAAAATTGGGGAAGGGTGGGACGGTCCTGGGGAGATGGCAAGGAAGAGAGAAAGGAAGAGAGAAAGAAAGAGAGAAAGAAAGAGAGAAAGAAAGAGAGAAAGAAAGAGAGAAAGAAAGAGAGAAAGAAAGAAGAAAAGAAAGAGGGCTGGTGAGATGGCTCAGC

>P593

TTGAACACCAAAGAGGCCCGGTACACGAAGACAAGGCGAAAAGAATAAGGAGGAGGAGACGTAGGAAAGGGAGAAGGTTAAAGAGGAAGAAGATAAAAGAAAAACGAGGTGGAAGGGAGA

>P594

ATAACCTAACATAATCTATGATTCTTATACTGTCGTGAGATGTGCGGAGAATATTCGATCCAAACGTAAAGAAGAAGGAGAAGAAGAAGGAGAAGAAGAAGAAGGAGAAGAAGAAGAAGAAGAAGAAGAAGAAGAAGAAGAAGAAGAAGAAGAAGAAGAAGAAGA

>P595

CGGTAGAACCACCGGTCCTCATCGGTAACAGTTTCTCCAATCAAGGACCTACTTACGATTTCTTGGGGACTCAATCTTAGTGTTAAAGAATTTCTTCAGGACTAGGGGCAAGGGGGGTGTGTGTGAGTTTATATGGGTAGGGGAGTTAAT

>P596

ATCCATTTTATTTATAAAAAAGGGTTATAGTGTCCCCAAACAACAATAAAACCTAAAGAGGAGGAGGAGGAGGAGGAGGAGGAGGAGGAGAAGGAGGAGGAGGAGAAGGAGGAGGAGGAGGAGGAGGAAGAGAAG

>P597

AGGTCGGTTCGTCTTGGGAAAGGTTTTACTCTTGTTCATGGATGCAGGCCTTCGACTTTTGACCGTGGAAAGTTTTTACCAGTAAAAGGTCCGAGTCGACGGTGTTAGAGAGGGACTGGGGTGGGGAGGTAAAGTCCCGTTTCGGTGACT

>P598

CACACGGTTCCAGAGGAGGTAGTTGCTGTTCTCACTGACCTTCCAATACTCCTTCCGCGACAGGCAGTAACTGAAGTGACTCCTACTTCACCTCCAATCCCGAGAGACACTGGGGAGGGGTAGTGTGGGGTTTACATGGGTGAAGAAAAC

>P599

ACTCCCTCCGTACGAGTCGTTTGGGACCGAATTAAGTTAACTTAATTTTAATGGATCCCGTGAGTTAGGGACTGGGGGATGGGGGTTGAGAGTGGGACCGACAGGACTTGAGTGAGACAT

>P600*

AGGGAAGAGAAGAAGAAGAGAGACAGAGACAGAGACAGAGACAGAGACAGAGACAGAGACAGAGACAGAGACAGAGAGAGAGAGAGAGAGAGAGAGAGAGAGAGAGAGAGAGAGACACACACACACACACACACA

>P601

TATGTAAAGTTTACAATAGGGGAAAGGACCAAAGGGGATGTTTTGGGGGAATAGGGAAGGGAGTAGGGGGGCGAGTGGTTAGGTGGGTGAGGACGAAGGACCGGG

>P602

AAGAGAAGGGGGAGGATGAGGGGGAGAAGGAGGAGGGAGGAGGAAAGGGGGGGAGGAGGGGAGAGGAGGAGGAGG

>P603

GGTCCGGTCGGATCAGATGTCTCACTCAAGGTCCTGTCGGTACCGATGTGTCTCTTTGGGACGGGGCGGGGGCTTAGATATTTTTTCAGA

>P604*

GAGGGTGGGCCCTTGTAAGTCTACTGTCATGGACTTCTTGAGACTTTTGAGTTGTTTCAT

>P605

TTTGGCTTCCACTGGGGGTGGGGGGGAATATCCAAAAGATGCTTGCCGTTGGATGAACTCGTGCATTAAGTAATTATCTATAGCAGTACATTCTTCCAATGAGAA

>P606

GAGAGAGAAAGAGAGAGGGAGGGAGGGAGAGGGAGAGGGAGGGACGGAGAGAGAGAGAGAGAGAGTGTGAGTGTGTGTGTGTGTGTGTGTGTGTGTGTGTGTGTGTGTGTACATGTGTTA

>P607

GTGGGTTGGGCACTGCACAGCGTTGGGGTGAGGGGACAGTAGGAACTGTGTGTGAGAGAGAAACATCACCCCAGGACTAAATAGGACAGAGGGCAGCCTAGAGTC

>P608

ATAAATAAATAAATAAAGGTAAAAAATAATCCATAAATGGAGTAAATGTAAAGGTTATCATATGGTTTCCAGGGGTATGGGTGGGTGGGGGTGAGGGGATGGGTGGGTGAGGGTAAAAAACCGGAACCACAAGGG

>P609

GAGTGGGTAGGGAAGTGGGGCGGAGGGTATGGGGGACTTTTGGGATAGCATTGGAAATGTAATTGAGGAAAATACGTTATAATAAAAAATATTTTTAAAAAATTAAAAAAAACGAAAGTGGTTCCAAGCAGCATG

>P610

GTGTACTGATACAAGTTAAAAAAAAGGATTAAAAATAAAAAAATAAACTATAAAAGAAATAAATGTAAAGTTTACAATAGGGGAAAGGGCCAGGGGGGAGAACCTTTGGGGGATAGGGCGAGAGGGAGGGGGACGAAGATATTCCCACAAGGAGATGAGAGGGTGGGTGAGGGTAGAGGGACGGGAGTCAAGGGG

>P611

GGGGAATGCCAGGGCCAGGAAGCGGGAGTGGGTGGGTTGGGTCGCAGGGTGGGGGAGGGTATAGGAGGCTTTGGGGATAGCATTTGAAATGTAAATGAAGAAAATATCTAATAAAAAAAAGTATCAGCGTCCATG

>P612

GGAAAGTACCAATATGGTGTTACATATCGTATATTAAACACTGAAGTAAGAAAGCATATATGTCATAAAGAAGAGGACCTTACGGGAGGTGGATGAGAAGAGTCAGAAATCTGGAGGGGGGTGGAGGAGGAAATTCCGATGTCGAATTTA

>P613

AAGAAGAAGAAGAAGAAGAAGAAGAAGAAGAAGAAGAAAAGAAGAAGAGGAAGAGGAAGAGGAAGAGGAAGAGGAAGAGGAAGAGGAAGAGGAAGAGGAAGAGGAGGAGGAGGAGGAGGAGGAGGAGGAGGAGGAGGAGAAAAAGGGGGTGGAGGAGGGGGAGGAGGAGAAGGAGGAAGAAAAAAGCTCTGTCCC

>P614

GGGGGATGGCGGGGGACTCGGTTTGGCTTTTATTTGGCTTTCCACAGGGCTTCGGAATAATTTGCTGGGTAGTGAGGAAACCTCTCCGCAGTGCATTCTGAGCTT

>P615

AAGCTTTCGGACTACAGAGGTGAGGCTGAGCGGAGTGGGAGCAGGTGGGGTCAGGGTCTACATGTGAAAGAAGTTAGCACTGACTTCCCTTCCGTTCTCCTCTTCCCTAGGGAAGTACGTGGTCCTCTTTTTCTA

>P616

GGATGGGTGGGTGAGGGTAAAAAACCGGAA

>P617

TCAATTAGTGATCAAGGGGGAGAGGCCCCTTGTGGGTGGGACCATCTCTGGGCTGGTAGTCTTGGGTTCTATAAGAGAGCAGGCTGAGCAAGCCAGGGGAAGCAAGCCAGTAAAGAACAT

>P618

CCATGCCCAAAGCAATTTAATTGGGAGGGTTGCTCACACTTTCAGAGGGTGGGTCCATGGCCAACTTGGTGAGAAATGTGGCAGCAGCAGGCAAGCATGGCACTGGAGCAGTAGCTTGGAGTTTACATCTGATCTATAGGATGAGAAAGA

>P619

CAGAAGTGAGAGAAGGGGGTGGGTGGAGGCAGAGGTAGGAAGGAGGGAGAGGAGAGGAGAGCAAGCTGAAGGAGCAAGCTGTGGCAGGGATGGACACTTCTGCTTCAAACGTCAATTATTAAAGTTCCCTTCCCA

>P620

CAGTTCTCGTTAAGTTCTAGGGGACAGGTCCCTTCCATGGTGGGTGTCACCGACCCAGAAGGGTGGATATATTCATTAGTTCTGTTGGGGGGGCGGGGGCTGTACGGGGTACCGGTTGGATTAGATCTACTAAGC

>P621

GGGCAACCTCTACCTCAAAGCTGGGGGTGGGGTGGTCCAATAACCATATTTTCTAATTTTAAAAAACCAATCCTAGTTTTAAAGTCCATATGCTATTATTGGTCA

>P622

GAAGGGATAAAAGAAAAAAAAAAGAAAATTATAAAAAATAATAATGCATAAAAGGAGTTAATGTAAATCTTACGATAGGGTTTTCAGGGGGTATGGGAGGGGGGCGTAAAAGGATGGGTGGGTAAGGGTAAAAAA

>P623

CCTGTCTCAGGAGGAGGAGGAGCAGGAGGAGGAGGAGGAGCAGGAGGAGGAGAAGGAAGGAGAAGAAGTCATAGCTACAGAGATAAAGCTGGAGTGTGGCAGTGCTGGGAAGGCAGAGACAAGCAGGCCTGAGCT

>P624*

GTTTCCTGGGGCATGCCTGAGTCATGGTTTTACGGGATTAAAAATTTTAAATAGCACACACATAAGCCCATATAC

>P625

GATTAAATATCTGGGGGGCGGGGGGTTGAAGGTGTAGTCTATTGTGGTGGGAAAATATGGTGGCCAGAGCTGTTTTAGGTATGGGGACACCTGTGTGCATTGTGCCCACAACCAGGAAGCAGAAAGAGTTGGATG

>P626

GGAATAAAATTAATTATCCCTCCTCTCTCTAACCTCTGGCATGACAGAAGTGACGGAGGGTGGGGTTTTATTTTTTCGGTAAACCGGAAC

>P627

GAAAAACAACAAAATAAAAAATAACAAAATTAATTAGTAAGGAAAGGAAATGTAGAGTATACTATATAGGGAAGGGCTAATGGAGAGGTATTGGGACGGTAGGGTAGGTAAGAGAGGGTGAAGGGATACTCCCACAAGGGTGAGGACGGGGTGGAGAGGTCGTAGGGAGATACGACCCCGTGGTTTGGAGGTGTCCTGGT

>P628*

ATAAAATTAAAAGGCCATAAACAACTCTGTGTGTGTGTGTGAGAGAGAGAGAGAGAGGGAGAGAGAGAGAGAGAGAGAGAGAGAGAGAGAGAGAGAAAGAGAGAGAGAACATTCCATATGACAGACAGCCCTGTTTGAGTCTTATGGTCTAGGGATAATAGAAGTCCAAATGTCTCAACT

>P629

ACAAATATATTGGTCCAGTAGCTTTCTCGGGGTGGGGCAAGATGTGAAAGGAAAGATATAACTAAGTGTTCTAGATAAGAGCTTCCTGAA

>P630

GTGGGACAGAGGTAGCCCTGGTTGTGGGGGTTGGGTTGGGTTGGGTTGGGGGACTGGGGGGTGGCAAGTTGCTGGGAAGAGCTTAGATAATTCTCAGACCCAGATCCAGGGCTTTGAATTGGCCCATTCGAACAT

>P631

GGAAGGAAGGAAGGAAGGAAGGAAGGATGAGGAGAGGGGAGGGCATGGAAGGGAAGGGAAGAGAGAAAGGAAAGAAAAGAAGGTTGTTCTGGAAAGACAGGAAAGTTGGTCATTTTTGCTTTAATGCTGTGTTAA

>P632

CGAAGTTAACGAACTGGAGAAGTAGAGATTAGGTGTATCCAAATTAAAGATTTGGACCGATACATCATATCCGAAGGTGTAGGGGTTTGGGGATAGAGTTCGTAGGTGAAGAGATTCGTT

>P633

GAGAGAGAGAGAGAGAGAGAGAGAGAGAGAGAGAGAGAGAGAGAAGGAGGAGGAGGAGGA

>P634

AAAGGAAAAGAAGGAGAGATGATGGGGAGAGGGGAGAAAGGATTATGGGGTGCTGTGGCTGGGATGGTGAAAGGACAACATGAACCCCATTTGTCTCCGTTTAAGGACCAGGCCTGATTTTAAAAAGAAGGCCATAAGGCAAAAGCTCCA

>P635

TAACCTTAAGGACCATCGGGCCTTGTGCCCTTTGACTTTACAGGGGACTCGACGACAGTGAACAGACCGGTACTCCGGGAGGGAAGATCATTTACGGGGGTGGAGACGGGGGACAGTCAGTGGGGATTCGTGTACGACGCGACACTTCGT

>P636

GTCTTCGAGAGTCCTAATACTGAGTCGTGTGGGCCAGGGTGTGAAGGTTAGACAATGAACGGTCTCCTGAGAGGGAAGATTGAAAGAAGG

>P637

GTGGTAGAAATGATTACTCTGTCCGCTGGAATCCAAAGAACCCCAGACCGAGGGAGCCACCGTCAACAATAACGTATACTTTCGACAGTCGGTGGGGGCGGGGGATTTATGTAGAAATAC

>P638

CCAAGGACACAGTCTTCCGAGATGTACGAACGAGACGAGATTCCCTCCTCGCCCCACCCTGCCTACCACGATCACGAGGAGGAGTGGGGTGGGATCACAAGAGATACCCTAAATGTCACC

>P639

CGGAGGGTTTCCTCGTGAACCTACGGTCCTTTCCGGACGGTCTTGGGTGGATGTCTGGAGTGACAGGTGACCATCCCTGTTGGGTCACGGGACGGTGACTGACTCAGGGGGTCGGAGGGAGGTCCAACGGGGAAA

>P640

GAAGGGGAGGGGAGGGAAGGGGGAGAGAGAGAGGGAAAGGGGAAGGGCGGAGAGGGGGAGAAATAAAGAGGAAGAGAGAGAACTCTATTAAAGGTCCAATTGATTATAAAAGAAGTTTCTAGTTCCTCCCCTCATCTTGGCAGGCTGAACCATATCCCCACCTTGATGGGAGATGGGGGTGACTCTAAACCAAGTGGTGG

>P641

GAAGACAGGTCGTGGGGATCGAGACGGTCGACAGGAAGGCCTCAGGGGAAGTCTCAGGACATGGGTCTGGTGGGTGGGTAGATGTGGAGGTAGAAAAGTAAGGAG

>P642

GGGAGGGGAGTGCCTTACAGAGAGGGGGCAGGGAAGGTTGTTCCAGGATTTCTAATAACCCACATTGAAGATGGACCAGAAACAAAACCAGGATGTCAAGATGGTGAACCTCCCAGGGTTATCAGGCCCAAAGCA

>P643*

TAATTTGTATGAGAGAGAGAGAGGGAGAGAGAAAGAAAGAGAGAGAGGGAGAGAGAGAGAGAGAGAGAGAGAGAGAGAGAGAGAGAGAACAAGAACAAGAACAGACTTGCTCACCAATGAAGCTGTGGGGTAACTCCAGACTCAGACACCAGATACAGGCAGAAC

>P644*

CTGTTGGAGACTTCC

>P645

AGTATATCACCGGGGGTGGGGGAATGTATTTACTTGAAGGGTCTATCCTGTCCCTGCTGTCCCTGGTCATTTTCTGTCTGGTATATACATATATATGTTTTGCCA

>P646

TTGCCAGTGAGTCTCTAAAAGCAAAGGGGGGGGAGGGCATCACTTTATGCATGTGACAGATGCTACACAAAGGCATGGAGGCAATGACTGGCTTACTTCTTAGCCAAACTTAGAGCAAAA

>P647

CAGGACCTTAAATTGTCCTAAGGGAACTTGAGTTGTCTATAGGAGGAATATGAGGACGAGACGGTGGGTCATGACCCTGATATCAGCACGCGGTGGTGCGGGTCAGGGATGGGATAAACAAAGAGGAGGAGAAGGAGGAGGAGGAGAGACGGAGAGAGACAGAGACAGAGAGACATACAT

>P648

GTCTCCGTCCGCCTAAAGACTCAAGCTCCGGTCAGACCAGATGTCTCACTCAAGGTCCTGTCGGTCTCGATATGTCTCTTTTGGACAGAGCTTTTTGGTGGGGGGGGGGGTTTTTTTTTTCTTTTTTTTTTCTCT

>P649

GGAGAGAATGGGGTGGGGAAAAGGGAGCTACCCGCAAGCATGAAGCATGTTTTAGTTTATACAGCTGCAAAGGTAATCATTATATGCTTACCAGGTTTTTTTTTGGGGGGGGGGGGGTCTACTGTCACAGGATAAAGATGCCACCAGGAGACAGAATCTGCACACATGAACTCAGTAGAAAAGCACTACTCACGC

>P650

TCAGATGGATACCTGGATGGGCACAGTGGCCCTCGGGAGTCGGGGGCAGGGCACGGGCATGGCAGGTTCTTCCTGAGCCCTGGAAGATGCAGGGATGATGGTCACTATGGACAGGGTGGGAAGGAACTTATGCTA

>P651

TGAGGGCTTACTTTCTGTATTTGTGTCTGGAATGTAAAAATATATGACCCGGTGAAAAATTGGAGGTACAATTAGGTGGAGGGGGATGGG

>P652

GGTCTTTGGGTGAGGCGGGAATGGGGGTGCACTTGTGTCTGTCATATCTGCGTCCTTCTGATACTTCTGAAGCCGAGGCATACAAAGCTGTTGATGATTGGTTTTGTTTCATGTCACCGCCATGTCACTCAGGCT

>P653

GATTGAGAAGTGGGGGACATGGGAAGGATTCTAAAGAAGATAACTGGGAGGGGCTGGAGGGAGGAAAGATTTGGGAAAGTGATATAATTCTATCTCTGCCACGCACTCTGGTCGAGTCAGCTTGACAGCTTGGAAGCTACTCACTGAAGA

>P654

CTGTTCTACTGTTTTTGAATTGACGGTGTGTAAAAGGAATTAGGGGAAGAGAAAGGGTAAAAAGAAATGGAGGAAGAGGGAGGGAGGAAGGGAGGAGGGAGGGAG

>P655

TAGGAGGTCCATATCGACGATACAGAGGTTGTAGTCCTAGGAGTTGGACGAACCCTGATCTAGTTAGGGGGCTCCCGGAGTATCTTGGGGTCGAAAGGGGGAATTCGGTGTAGACGACAGGAACAATATTGTTTGTACTCCTTGTCACTCCGCGTCCGACCGACACTGGGAACGGGTGGTGCCAGAGGAGGACTA

>P656

AGAGAGTGTTTGTGCTGGGTGGGTGGTGGGGAGGAAGATGCTCTCAGGGCAGAAGCCAGTGGTGGTGACCTGAGGAAGGGGCGGACAACTTGGAAGGAGGAAGAGGCTTCTGGGAGATGG

>P657

CCGCTCACCCCCTCATCCTGGTACTCAAGGGGACAGTGTCGAGAACTTCCTAAATCCCTAGTCCCTCCGGTGACCGACAGGTGGGAGGTTATGGGTGGAGGGTGTACGTGAATCATTTTATCCAACAGACATCCA

>P658

TAAATCCGAAAGACAAAATCGAAAGAGGGGGTCCGGTATGAAAAGAGGGAGGGGAAAGGA

>P659

TGTTAATAACACGAGTCCCGAAAGTATGGGTTCCGGAAATACCCGAGGTAGGGAATTTCCAAGGGTGGAGATTGGGGAGAGGAAAAGGTTGACAAGCGTAGAACGTGGTGGACTCGTCAT

>P660*

ATTATCAGTCTTCGTAGTGTCTCTTTCACGGACAACTTGTTTGGAAAGAAATAGAAAGAGAGAGAAAGAGAGAGAAACAAACAAACAAACAAAGAAAGAAAGAAAGAAGGAAGGAGGGAG

>P661

TGGATATGGGGGACTTTTGGTATAGCATTGGAAATGTATATGTGCTAAATACCTAATAAAAATGGAAAAAAAATAAAAAAAATTATGTATAAGTAAATCTAAGGA

>P662

CGTACAGGGGAATGTCAGGGCCAGGAAGTGGGAGTGGGTGGGGGAGTATATAGGGGGCTTTCGGGATAGCATTTGAAATGTAAAAGAAGAAAATATCTAATAAAAAATTGTTTTTTTAAAAAAAATTCTTTAGAT

>P663

AGGTCTATGTGTGTACATGTGGACGTGGATGTGTACGTTTGTACGTACATGTGTCCGTACCGCCACCCTCCACCCCGTTCGCGAGAGAGAGAGAGAGAGGGAGAGAGAGAGAGAGAGAGAGAGAGAGAGAGAGACCCTTCTTTCTAGATA

>P664

AGGGCCAAAAGGGGGAGTGGGTGGGTAGGGGATTGGGGGGGTGGGTATGGGGGACCTTTGGGATAGCATTGAAAATGTAAACGAGGAAAATACCTAATTTAAAAAAAAAAGAAGAGCAATGCCAATAAGTTGTTC

>P665

TCCATGATGAGCTCCTGAGAAACAACAGATTTAACAAGTGGGGGGGGGGTGGTAAAAGTGGGTGATCTTCTCTATTGACAGAGGCACCTCATGCTAAACTCCATCAGCTTCCCTGGAAAAGACTCTTTGTCAGTCTTCAATGGCTATACT

>P666

ACAGAAAGGAAGAGTATAGACGGAGACGGGTCAGGACTTATCAAGGACTCAATTTAAGAAAACCAACGGACTGAAACCTAGACATCCAAACATAAGGGGGGGGGGGGGGGTCTGTCCCAA

>P667*

TGACGAAGACTTCTT

>P668

GGTTTTTTTTGGGGGGGGGGTTGAGACAGGGTCTCACTATATAGCACCGGCTGACCTGGCTGACCTGGCACTCACTATGGAGAGCAGGATGGCCCCAAACTCAGAGGTCCATCTGCCTCT

>P669

ACAGGGGAACGCCAGGGCCAAAGTGTGGGAATGGGTGGGTAGGGTGCTTTGGGATAGCATTGGAAATGTAATTGAGGAAAATACATAATAAAAAATATATAAAATAAAAAAAACATGTTCATAAGCCTATTTATT

>P670

CTACGGTTGGTGTTCGGTTTCCCTTGATTGACCCTTTATCGTACCTGAAAACTTTGGAGTTTTGGATGGGACACACTATGTGGAGGAGACTCTTCCGGTGTGGAGGTTTAGGAAGGGTTT

>P671*

ATGCCTAGACAGAAGGCAAAACCCACTTTAACTGTGGTTGAGAAGTCGTGGTGTAAGTCG

>P672

ACTCTCCTCGACCTAGGGCAGGGGTGGGGACTGTGGCTGCCTTCCCACCCCCACCAGTCCTGCCTCACTCCACACCTGGCCAGGTGAGGGAGCTCACAAGGGAAGGGAGAGGACGGAGAGGTTCCCTCAGCCCCCTGGCAGCTCTCCAGTGCCTGGGCCGCGAGCTGCTCCGCTTCCTGCACGTGGTCTCTTCTTGCTGG

>P673

AAGGTAGATGGTAGTAGCTTAACAGGGGTGGGGTGGGGGTGGGGGTGCAGTGCTGTGTGTGTTATATTTCTGTTGCTGTGACAAAACAGCACAAACACACGCAACTGGCATTCAAAGGAAGGGGGTTTATTTTAT

>P674

TAGGTCTGACCCTACGACCTCCGATTCTTGGACCATGGACCCCGACGGGGAACAGGTGGTGGGAGTCACACCCCAGTCACCGAAGTGTGTCGGTCTGCGGGTGGGTCCCGTCACACCTTT

>P675

TTAATTTGGGGGGGGGGCAGTATTTCAGTGCAACATGGTAGAGGAAATCTCGTAGCTCCATAAAGTCACCTGTTC

>P676

TGGGACTCCCCGGGAAGGCGCGTGAGTCGGGCTGTGGCAGGCCTCGTCGGGCGGTTGCCGAGTCGTGAGTCGGGAGCCCAGGGCGGCGGGCACGCGTGGAGCGCACACGGCCGGGCGGCGGGGGCTGTGTGAGTG

>P677

CCTCTGGACCAAAAATAGACGTCTTGGGTGGATTCACACCTTCCTTCCTATGCTGAGGTACTCTCAACAGGAGACGGGAGGTGTACCCGTGTCTCCGTCTAGAGGGGGGGGGGTATTATC

>P678

GAGGGAGAGAGTAATTATATTAAGAAAACGTGCGGTGTGCATGCTGGGATGAATTGCTGTTGAGAGAAAGGGAAGATGACCTAAATAAATGAAAAGATTTGTCAC

>P679

GGAAGCAGGTGTGTGTGGAGAGGAAGCAGGTGTGTGTGGAGAGGAAACAGGTGTGTGTGGAGAGGAAGCAGGTGTGTGTGGAGGGTTCTT

>P680

AGAGATCATGGGAAAAGTTGGAGAGAGGATGGGGTGGGGTGGAAAAGATGTAAATACAGTACACAATTATGATATTCCCAAAAATAAATAAATATTTTGAAAAGA

>P681

GGTGGTACGGACGTTATCATCATGAGACCCCCAACTCCGTCCTCCTAACTGTTAAGTTACGGTCGAACTATACACCTTACTTTCATCTACCGGACCCGGTATTTTGTTCGCGGGGGGGGGGGGTATTCTCTTTCACCGAATACCTTTGAT

>P682

TTTCTTCTAGACTCTAGTAGGTCGTCTTTCGGAACCTCCGACCCCGGAGTCCTCCCGAATCAAACAATGACAGTGGGTGCTCAGGGTGGGCGGGGGGTGGAGACGGCTCCGTCCGTCGGT

>P683

AGAGGTCAGGTGGGGTGGGGGCATCCACGTGGAGACAGGGTGGGGTGGAGAGTGGGGGCATCCGCATGGAAATGGGGTGGGGTGGGGAGGAGGTGTGGGATGTGGAGCAGTCAAAGGGTGGATGGGGGGATGGGGAATGGAATATGGAGTGTAAAAAATGAATTAAAAATAAAATAAAATTTAAAAATATTTCATATACA

>P684

ACACACAGAGGGGGGCGAGAGGGAGGAGAGGAGGAGGGGGTGGGGAAGAAGGAGGGGAGGGGAGAAAAGGGGGAGGGAGAGAGGTAGGGGGAGAGGAAGGGGAGG

>P685

GAGGGGAGGGGAGGGGAGGGGAGGGAAGAGATGACAGAGTATCTC

>P686

GGGGGAAAAGAGAAGGGGAGGAGAAGGAAGAGAAGAAGGAGGAGGAGGAGAGGGAGGAGAATAAGGAGGAGTAGTAGAAAGAAGAGAAGAAGGAGAAGGAGAAGGAGAAGAGGGAGGGGCTATGAGGAGGAGAAGGAGGGGGAAGAAGGAGAAGGGGAGGAGAAGGAAAACGAGGAAGAGGTGTAGAAGAAAGAGGAGAA

>P687

AGAGCGTCCCTTGTTCCCGTGCCCAATAGGAGGACGGAGCGGGAGGGAGGCGGGGGTGCGGTGCGTGCCGCGGATCCCCGGGCGCGCACGCGGGGCGCTCGCGGACAGACCGACGCGGGCGCGCGGCCGCCGAGGGTCCCGCGCGCGGCGCCACGTACCTGACTGTAACCCCGCGCCGGG

>P688

ACAGAGGGGTCGGTGAATGAGTCTCGACTGGAGAGGGTAGGTAGGGAATAGAAGGTTGAGATTGGGGAAGTAGGGAAGTCGGAGATTGGGTCTTATTTGGGACGGGACAGGTCGGGTAGT

>P689

GCTTAAAAGGAGTTAATGTAAAGGTTACGATAGGGTTTTCAGGGGGTATGGGAGGGGGGGGGGTGAAGGGATGGGTGGGTAAGGGTAAATAAAACCGGAACCGCA

>P690

CCATGCTACGTGGTACAGAAGGGGGGAGGGGGGGAACCAGCCATAGACCCTCTGCTGCCATGCCTTAGGAGAGGCTATAGACCAAGCCATGCCACGCTGTGCAGGCAGAGGTCACCCAGA

>P691

TATTCCTATGAACTGGGATATTTCAGAGAGAGAGAGGGAGGGAGGGAGAGAGAGAGAGAGAGAGAGAGAGAGAGAGAGAGAGAGAGAGAGAGCAAACCTATGTGACCTAGCTTTGTTGTGAGGATAAAAATGAAACAATGCACATGACTCTAGTTGGGGGATAATTGGATCCTGGCAGTC

>P692

GTATGGTGAGTGGGGCATTAGAACTGGGAGGCAGAGGGAATTCAGAGGCTGCACACAGTCTGGCCATTGGTGGATGCTGACCTGTTAGAAGCTGGACCTGAAGACCGAGGATGGGCGAAG

>P693

AAGGAATGGGGGTCCTGTTTGAATTACCAGGGGAGGGGGTGGAGGAAGGGTTGGTATGGCATATGCTGTAGAGCCTCACTTTGCACAAGCAGGGCACCGAGGCCTAGGGATCTCATTTGGCTTCTGGTCGCATTT

>P694

TGAGAAAAAAAAGAAGTAAAAAAAGAAAAAATTATAAAATAATCCATAAAAGGAGTAAATGTAAAGGTCACGATAGAGTTTTCAGGGGGTATGGGAGGGGTGCTGAGGGGATGGGTGGGTGAGGGTGAAAAATCGGGACCGCAAGGGGTATAACCCCGTATATTT

>P695

AGGAGGAGGAGAAAGAGAAGGAGGAAGAGGAGGAGGAGGGAGGAG

>P696

GGGATGAGAGTCATATCAAAGACCCTGAAATAAACCCACACACCTATGGACACTTGATTTTTGACAAAGGAAAAATTCCATAAAATGGGGTGGGGGGAAGCATCTTCAACAAATGGTGCTGCTGTAACTGGATCATGTGCATGTAGAAGA

>P697*

ATAACTACTGTTAACGTGAAATGTCACCGT

>P698

AAAATTATGTCCACAAGAGATTGTCCACACCATGGGTCTTTCGGACGTCCGGGAGGGTCCCTGGTCGTTCGTCCTCGGTCGGGGTCTCCGTCTAAGAATCTTTTGTCCTACGAGGGGTGGAGGTGGGAGTTAGGAAGGTAGAGGAGGGGCAGAATTGAAGGTTTT

>P699

TGTCTCCAAAGTCGCTCAGGGTACCCTGTCCCGGTGTATCTCTAGTTTTCCGACTGTCTTGTCTCTTCGTCACAGTCTTCGTTGACCCGTCTCCCTGTATCGTTACGAGTCCAGGACGGG

>P700

GGGGGGGGGAGGGAGTATCCTCCTGTAAGCAGGGGGAGGGAGGATGAGATACAGGGTTTGCAGACGGGGAAATTGTGAAAGAGAATAACATTTGAAATGTAAATCCATAAAATATTCAAGAAAAAATAATGTATA

>P701

CTATCGTGGTCCGAGGACGGAAATTCCGTGAATGTCGGGTACGAG

>P702

TTCCAAACACAAACTCTTCGGTGTCGACAAGTGGAAAAAGGGGGAGTCTTCGGAAAGACTCAGGACTCGGAGTGTGTCGGGGGTCCACGA

>P703

TGTAGTGATAAGTGGTCCTTTTGTATTGAGTTTTGGTATCACTCCGTGGTACAATGAAGGTAAACGATTATACTC

>P704

GAGGCTGCCTGTGAGCAAGGGTGGAGGGGAAAGAGCAGGAAAAGAAGGCACTGGCTTTTTATTAGAGGGTATAGCATGAGTGCATCGGGAAAATCTCCACCTCAC

>P705

CTGGGGTGGGGGTGGGGGTGCTCTCAAGCTGCCCAGGGCTGAGCTCGGCTGATCCACGGGAGCTGGCTCAGACATCTGGGAAACGTAAAGCTGACATCACCACCC

>P706

GCAGCTGGAAACAGGGAAAGTGTGCAGAGGGCGGGAGCAGGTAGTGAGCACCTGCAGGCCAGGTAGGGAGGAGGGGCGGTGGGCGACCGGACAGAGGTTTTGAGAATCAAGTAAATTAATTAATACTGGAGAGGGGCCTTGGGTTGAAGCCAGATTCCAGTGGCGTGAGGTGTGGAATCAACGCGTCACTGAAGGCAAGA

>P707

AGGAAGGGTGGGTGGCCAGGCAGGTTGCTGAGGTACAGAAGTAGAGAAGCTGGGAAGCAACTCGGAGACACATAGCTGACACAACTTAGTTACTTTTCATTCTGAGGGGCTCGGGTGAAAGAGATGTGACTGGCAACTTCAGGTTTCCCAATGGGATGATTAGAT

>P708

CAAGTCAAAGAAATATGTATTTTGCTTTTATTAACACAGTGACCACGACGTCCTTCTAAGACTCCTACCGTATGAACATTTAGTTTCATGTTTACAGGGGGGGGGGTATTATCTTGATGGGTTTGATTAACGGTG

>P709

TACCAAAATAACAAAAAAAAAGGTAAAAAATAATCCATAAATCAAGTAAATGTAAAGGTTACGATATGGTTTTCAGGTGGTATGGGTGGGTGGGGCTGAGGGGAAGGGTGGGTGAGGGGGAAAAACCGGGATCGC

>P710

TGTCGTAGATACACTCAAAGGTCTCTGTTCCTCCTTCTTCGGGTCGAATGTGAGGTGTGTTATTCTGTACCTAATCGCCTAAATACGTAAGTCTACGTGAGGGTGGGGAAATTCCTGGGGGTACGGGAGACTGAG

>P711

ACCAGGGCCAAGAAGTGGGAGTGGGTGGGTAGGGGAGCAAGGCGGGGGGAGGGTATAGGGAACTTTTGGGATAGCATTTGATATGTAAATAAAGAAAATGTCTAA

>P712

GAGTCGGAAGGGTTAAGACACTGGTAAATCATGTCAGGTAGGGAGTACAAAGTTACTGGGGGTTGGTATTTTGATGTAGTGACTATAAAGTGTGGCCATTAAAACGATGACAACACTTAATATTACATTTATAGACTATATGTCCTACAGACTATACACTGTGGGCGGGGGTTTCCGTAGTACTGGGTGTTCAACTCTTG

>P713

GAGGGGAACGCCAGGGCCAAGAAGTGGGAGTGGGTGGGTAGGGGAGTGGTGGTGGGGGGGGGGGTGTGTGTGGGACTTTTGGGAAAGCATTTGAAATGTAAATGAAATAAATACCTAATT

>P714

CTAATGGCAGTCCACGTCTTTCGTCGGGAAACAAGGCACCCGTAAGGGGGGGTGGGGGGCCTAAACTGACAATAAATGTCCGGTACACTC

>P715

ATGGGGGGGGGGGGAGGAAGGGAGGAAGAGAGGAGGGTGAAGAGAGGAGAAGGGGAGGGAGGAAGGGAAGGTGGGAGGAGAGGAGGATGGGAGGAAGGGAGAAGCACACAGAAAAGTGTACAACTTGGCCTCAGAACTTAGCACCTCTTG

>P716

CAGGGCATGGAGGTGGGAGTGGGTGGCTGGGTGGGGAAACACCCTCATAGAAGCAGGGGGAGAGGAGATCGAGTAGGAGTTTTCTGAAGTGGAATCCAGGAAAGGAAATAACATATGAAA

>P717

GAAGATCTAACAGGGTCTTCACGGAGGCGGGGGTAAAGTCAACAGACAGGATAAAAAGAGAGCTACTCAAGAGGACGGTGAACGAGGGAGGACAAGGAAGGTGGGGTGGGGTGGGGTAGGGTAGGTAGGTGTCACAGATAAGATAAGGGA

>P718

ATTAAAAAGTCTCCTTTCTCCGGTAACTCCTTGGGTATGTCCGAGGAGACAAAAGGTTCATTACCCATATATTGGGAGTTGCCGGGGATCCTTTTTGGGTTGGGG

>P719

GGTAGAGGGTGGGAGGGATTTGGGAGGAAGAGGAGGAGGAGAGGGAAAAAGGGGTCAGCATCAGGTATGAGAAGAGACAGGGTTGATACAGAGAGGGTCAGGAATTTGAATAGAGGTATGTAAAAATGGGGGATGGGGAACTGGGGGGTAGCCACCAGCAAGTCCCAGATGCCAGGGAAGTAAGAGGCTCCCAGGACCCA

>P720

CCACCACCACCACGACTACGACAACGACGACGAGGAGGAGGAGAAGGAGGAGGAGGAGAAGGAGGGGGGGGGTCG

>P721

GAAGGAGAAGAAGAAGAGGAGGAAGAAGAAGGAGAAGAAGAAGAGGGAGGAGGAGGAGGGGGAGAGGGAGAGCATGAAGGAGGAGGAGGAGTAGAAGGAGAAGGAGAGGAAGAAGATTTGGAAGAGGTAGAAGGAGAAGAAGAAGAGGAGGAAGAAGAAGGAGAAGAAGAAGAAGAAGAAGAAGAAGAAGAAGAAGAAGA

>P722

AGAGAGAGAGAGAGAGAGGAGTAGTGGGTAGGGGGTAAGTACATCCTAAACATATACATACACCACACACTCACAAAATCAAAGTGGTTAATAAAATGGCAGAGTTAACATAGTGTGATG

>P723

GGGATATAGAGGAAGATAAGTAGGGTCTTAGGGGGTGGGGGTGAGTCATGGGGGAAGTGTTGAAGTAGAAGAGAA

>P724

TTGAATTAATTATCCCCCCTCTCTCCAACCTCTGGTATGACAACCTTCCCGTTCGGGAAGTGAAGGAGGGTGGGTTTATTATTCGGTTAA

>P725

TGAAGACTAGGAGGACAAAGAACTACAGGTACAGGTGAAACACGCCCCGAAGTACTATCGATTCTTAGGATGGTTGGTATAGGGGTTGGGGAGGCGACAAAAGGAACACCCATCGTTGAACTCTTCAATCGAGATGGGTGTCGTCACTCTGGTAGGTGTCGGGGTCTGAGGAGAGAGGTGGAAACACAGAACGTCGGGGA

>P726

ATAAATTACATAACACATACAATCCTTATCATAACTTATTTATTTCTATTCTTTTTACAAACACGTATGAGTCAAGTCTATATTGTGGGGAGAGGACAGGGACGGGGTAAAGAGTCGAGATGGGGAAAAGGGTGA

>P727

TATAGCAAAACTGGGGGAGGGGGAGGGGCTGGCGGGGAGAGTGACAGTGACATCAAAGGGGGAGGCAAGCATTGTGAAACACACCACACATTGCATGGCAGCCCCAGCAGAGTCCTAGGTATTCCCCAAGGACCA

>P728

ACAAGAAACCACTCCTACCTATGTCATTCGATTTTTGTGTTCCCCGGGTATTTGAGTCTTGAGTCAGAGAGAGAGAGAGACAGAGGGGGAGAGAGAGGGAGGGAGGGAGAGAGGGAGAAAGAGAGAGAGAGAAGGAGGGAGGGAGGGAAAGAGAGGGAGGGAGGGAGAAGAGAGAGAGAGAGAGAGTGTGTGAGAAGGTA

>P729

CCAAACACCTGCCAAGAAGCAACTTAAGGAAGGAGGGAGGGAGGGAGGAATGGGGAGAGGAAGCGAGGGACTCTGTTAACTCACAGTTTGAGGGCATACAGCCCGTTTGAGAGAGGGAGACATGACGGCAGTGGCGCATGGTAGAGTGCTGTACAGCTCTACAGA

>P730

GACGTCCTGAAGAGGAGAAAGAGGTGTAGGAGGAGTTGTACAAGA

>P731

GGGAAAGGAAGTGCAGAGCCTAGCAGACTTGAAGGGTCAGGGTGGGAGGATAACAAGGGGGGGGGATATCCATTCAGAGGAGAAGGGGAAGGGGGATGAGAGAAAGATTGTGGGAGGGGATGATTGGCAGGAGCCAGTGACCAGGATGTAAAATGAATAAGTAAAAAATAAAATAAAATAATAAATAATAATAAT

>P732

AAGAGAACTTCGGTGGGACTGTGGGGGTGCATTTCCCTAGGTGGG

>P733

GTGAAAAAAACGTTGAACTTTTTTTTTCACACTAATTCGTGAAAAATAATCTACAAAAGAAGTAAATGTGAAGTTTACGATAGGGGAAAAGATCAAAGGAGAGGTTTTTAGGGGATATGGGAAGGGGGAGAGGGACGAGGGGTTGGGTGGGTGAGGGTGAGGGTCGGGACCGTAAGAGGATATGACTCAGTATCT

>P734

GAGCCCTCCGTCTGTGTCCGTCTAGAGACTCAAGCTCCGGTCGGACCAGATGTCTCTCAAGGTCCTGTCGGTCCCGATGTGTCTCTTTGAGACAGAGCTTGGCGGGGGGGGGGGTTTTTTTTCTACCTTTTAATATTTCTGTCACTTTTA

>P735

GGAGTATGCAGATGTGGGTGACTTGAGACCAGGAGTGTCAGAGGTGGCCATGTGGGGTAAACAGAAAGGAAAATGGAGGCCACTAAGACC

>P736*

GAGAGAGACGTGCATACATCACACATATGAACATGTATGTACATCTCACCCACCCAAAAATAGTAAAGGGGAAAAAATCAGTGAATATCT

>P737

GAGGGATTCTCCTTTACAGGAATGGCGATTAAGTAAAATTTTGTAAGTAATTTGTGGGTAATGCACGGACATCGACACGATCCTCGAGGTCTATGTTTTGATTCTCGTCTTGGGGGAACAGGGGGAGAAGTGGGGTGGAGACACGACAAACATGGAGCCTTTGTC

>P738

CCCCCACCGAATCCTGACGACTCGGTCACGTCTGAGACGGGTGGGGGGTCCGGTTCGTGGATAGATAGGAAACAG

>P739

AGGAAGGAAGGAAGGAAGAGAGAGAGAGAGAGAGAGAGAGAGAGAAAGGAAGAAAGAAAGAGAGAGAGAAAGAACAAATTCAAACATGTGTCAGAGAATAGAAACCAAGAAACTATGAGG

>P740

GGGTGGGGTAAAAGATGGGTGGCAGGGACTCCACTCAGTGATGATGGTGATGGCTGGTGCTCAGCTCTGCTGTCTGCCTCTGAGTCTTGC

>P741

AGGGGGGCTTTGGGAGAGAAGAAGAAATGTATAGAGAGGGTATTGGGGAGTAAGAGAAAGTGCTATGAGGGGTTAAGAAAGACAAATGTCATATTTTCTTTCATATTTAGAAATATTTGCACATATATGTATACAGACACATACATAACC

>P742

TGGGTGGGTGGGGGTGAGGGGATAGGTGGGTGAGGGGGAAAAACCGGGACCGTAAGGGGA

>P743

TCCTCTCCTAAGACATTGTATGGTGGGGGTAGTGGGGGCAGGGAAGAGAAAGAGAAGGGAAGAAAGCAAAAGAAATTAGAAAGGAAGGTATCTCCCCTTTTGATCTTGAACAACACAAAT

>P744

GGTGGGCCTGGGCCTTCTTGTACCTGTCACATCCCTCCACAGTACTGGACGGAAGGTATAGAGCATGGGAGCTATGGCCCACACCATAGAGAGCAAGAGTTGATCTACACAGAGACTCTCTGTGGGGTGGGAGGGAGAGGGGCTACAATTCAGATGGACAGGGCTTTTTTTCTTTTTTCAAGACAGAGTTTCTCTGTGTA

>P745

TTTCTTTGGTAATTTCGGCCCGGGACTCCTTAACGTGAAGGGGGGACCTTCCGAAAATCCTGTCTCATTTTTACGGAACTGAGTTCGGGGGTACGGAGAGACCTGGGGAATTTAGGTCCGCGGTTCGCACCGGGGTCAGGGGCTTCGGGAAGACTGGACGACCAGCCCCGGTCTCCGACGGTCGTTTGGTTGGAGGTGGG

>P746

AAAAATAAAAATAGCTTTGAGCTGGGGGGAGGGGAGCATCTAGGAAGGCCGCTCAATGGGTAAGTGCTGACAGCAGAAGCATGGAGATCTGAATTCCGATCCCAGAACTACACAGAAAGC

>P747

GAGTTCCCTGAGGTCAATGAACGAGATATAGGTCTCGGTGGGTGGGTTTCTTTGGTTTTC

>P748

TATGGGGAGGGGAGAGTGGGCAGAGAATGGCGGGTCTGAGGATTAATGTTGATGGCTGTCTGTGGCTTTCTGGTCTCTCCCTGGCCCTCA

>P749*

ATTGGAAATGTAAATGAGCTAAATACCTAATAAAAATGGAAAAAA

>P750

ACTCAAGGGAAGAGGAAGGAAGTTCAAGAAGAAAAAGGTAACGGTAAGAGAAGAGAAGGGACATGTAAAAGGATTCCGTACCGTCGAGGAAGGGAAGAGAGGAAGGAGAGTCAGGGATTGGTCCCTTTGAATGTG

>P751

AGTTCAATGAAATCTTTTAATCGTTCGAATGAACCTCCGTCCTCACGGATAGGGAATCACCGACATATCACGCAGTGATCGGTCACGGAGGGAGGTAGGGAGGTAGGGAGGTAGGGAGGT

>P752

GACATGATAAAAACCTGATGGGCCCTGGGTATGGGTAGGTGTAGGGGCTAAATCATTTAACACACAAAATTTGGCAAACTCATTCTTGTATTTTTGGGAATTAGAATTCTGTATCAAACCTGAAGCATATACAGT

>P753

AATAACTTTGTATTCCGAGACTACCCGTTGACTCCTTTGATTATGAGTTGACACTCAAACTTCTCCAGTCGAGTCGTGGAAGCCTTTTAAGGTGGTCTGGGGAGGGGAGGACCTTCTCTCTCCAGTAACTCGTGTATCCGTGGGGAGGGGAGGACTTCTATCTCT

>P754

TCAAGAACCCCGAGTGGAGGAAACAGAAATTATCGAGGTAATAGATTTAGTCCTGGATCACAGACGAATGCTTTATACTCGGGTCTAAAGTTTGTTTTTGTGTAA

>P755

TGACCTGACCTTGATCCCAGAGCCCACAGTGGAAGGGGGGGGGGTGCTGACTCTGAAAAGCTGTCTTCTTAGCAGTACATGTGCACCAAGGCAGGCAGATAGGAGCACGCACATCACACCACACACACACACACACACACACACACACACACACACACACACACA

>P756

ATTGTATTGTAGAACTAACTAAGAGACCCTTAAAGGGTAGTACGTGGGACTAGTGTAAATGAAGGGTCAGGAAGATACAGATGGGGGATTGGGAGCGGTGGGGACGTTTCATTTTTATTTTTAGTCCTTTTGTTTGTTTTTCAGGTTAAACACAATAAATAGATGAATGGCCTTGTACCAGTTTGAGAGTCACCGGACGG

>P757*

TATATATATACAGAGAGAGAGAGAGGGAGGGAGAGAGAGAGAGAGAGAGAGAGAGAGAGAGAGAGTCCAAGCCAACACGTTGGCTCACACTACTGTACTCATGTGTCATTATCTCTTTGCCATACGCACAAGTCA

>P758

TGTGCGCGCCGAGTGCCAGGGACCGGGCGGCGTGGGGGCCCGAGCGGCTCGACCCGAGCCCTGCGTCAGGCCGAGGCTCGCGCACACCTGCCCGCCGCCGCTCCGAGCCGCGTCATTGGC

>P759

CAGTGTGAAACTGGGATGGGTTGGGTAGGGTTCTCTAACTGCTACTGTGATAACCGGGAACCTCAGCGAGTGCATTAGGAACCGTGCAGGGGGAGGTTAGCTGGTCTCTGGAGGAGGGCTCTGGAGGCGTGGGTCTCAGGCTGTAAACGTCCAGGAGGGACCGCACTTGCTAGTCTCTGCACATGCAGGTGAATAGTTCA

>P760

TCGAACGCAGTTCAAGTATATACTTTTCCCCATCTACTTATTTATTTATGTAAATGGGTCCTTTGGGACCAAAGTAGGGGTTATGGGGTT

>P761

CCTCCTATCCCGAGACGTTGTGCCCCGACAAAGATGGTAACGGGTCGTAACGAGGACTCTCACCTCTCGGACCCGTCGAGACGGGGGGGG

>P762

AAAACCTGTCTTGAAAAACCAAAGTGGGGAGTTGGGGGGCGGGAGGGAGAGACAGAGAGAAATGGGAAGCAGCCCCGCCCCTTCGTAAAAAGGAAGGTTTGTGTGCCCAGTCTTTTGCTGTGAAATCTAATCATATGAATCAATTTGAAA

>P763

TATTTTTGTTTATTTATTAGTCAATTTTCTTGACTCACATTCGTGACAAGATTAAGGTGAGTCTCGAGATATCTCAGTCAGGTGATTACGTCATGGGTGGGTCGGATACGCACAAGTGTGAGTGACCGTTTTGGA

>P764

ATGCCCCAGTGTAGGGGAACTGAGGGAGGGAGGTGGGAGTGGGTGGGTGGGTGGAGGAACACCCTCATAGAAGCAGGGGGTAGGGTGGGGGTGGGTAGGTGGGAGATGGGAAAAGGGGATAACATTTGAAATGTAACTAAAGAAAATATCCAATTTAAAAAGAAAAGGGGGAGGAGAGAGAGAGAGAGAGAGAGA

>P765

AGTTGGCCTGAAGACATACCTGGTTTGGGGTGGGGTGGTTATCATTACTGTTAGTTGATGTCTGAAGACACAACCAATTGTAGGCAGCACTATTCCCTAAGCAGG

>P766

TAGTGAGAGAAGGAGGAGGGGGAGGGGGAGAGGGAGAAAGGGAGAGGGGGAGGCGGGGTG

>P767

TTTCATTTAAGATTTCCCCGACAATTTCCTCTCTACCGAGCCACCGATTCTCGTGAACGACGAGAAAGTCTTCTGGGGGGTGGGGTTAAG

>P768

AGAAAGTTTAGTGGTTTGGAAAGAAGGGTATTTAGTATACAAGTGGAGATTGGATCTCAGGGTGTCGTATCTGATCTGTGTCGGAAGTTATAGGAAGGGGAGAGACGTGGGTTGTGGTAGAAGGAATTTAGGTCG

>P769

GGGGAATGATTGGGAAGAGAGGCAGGAGTGGGTGGAGGGAACCCTCATTGAAGCGGGGGAGGGGGAGGGGGAGTAGGATAGGGGATTTGTGGAAGAGAAACTGGGGAGGGGGATAGCATTTGAAATGCAAATAATTATAATAACCAATAAAAAATCCTAGAAAATGACTAATTAATTTGACATTTCTAGAGTTCT

>P770*

TGTGTGTGTGTGTGTGTGTGTAGAGAGAGAGAGAGAGAGAGAGAGAGAGAGAGAGAGAGA

>P771

ATTCGTGACTCGGTAGAAAGTTCGGGAATGTGGTAGATGTCACAACTCGTAAAAATTGTCACGGGATTACTTGGGAGAGTCTAAACGTGTCGGGCAGTAACGAAGAACGTTATTTTGACA

>P772

GACGACCGGTTTTGTATCCTTTTAAAACCCATTGTAGTTTTTTTTATAACTTTTGTGTATTTCATATAAATCTAAGTGGTGGTGGTGGGGGAGTTTTTTTTGTTTTGTTTTTGTTTTTGG

>P773

TGGGTGGGTGGGGGAGTGGGTGGGGGAGCATGTGGGGGACTTTTGGCATAGCATTGGAAATGTAATGAAATAAATACCCAATTTAAAAAGGGGGGAGAAGTTTAT

>P774

TTCAAGCTGGTTTGGGTGGGCTGGAGGTTAAGTAAGGGAAATTACAGGTAATAAATCATGGGATTTCGAATGACAGATTGGTAAAAGTAGCCACAGAGATAACAT

>P775

CCCAAAAGTCAGAGGTCACAAAGGACTTCTGAGTGAAAGAGATAAAAGAACAGGGACACGGACGGACGGACGGACGGACGGACGGACGGAAGAAAGGAAGGAAGGAAGGAAGGAAGGAAGGAAGGAAGGAAGGAAGGAAGGAAGGAAGGAAGGACAATAGGGAAATCCTACAAAAACCAC

>P776*

ATTGAAAATGTAAACGAGGAAAATACCTAATAAAAAAAACTTACG

>P777

TATCAAGTGTCATTATAAGGAATACTAAAAAAGTCTACGGAATAACAAAACGGATGGATGGAGAAAGGAAAGCAAAGATAGAAGGAGGTAGGAGGAATGGATGGAAAAGGGTAAACGGGT

>P778

TGAGATGAGACTTGGTGTGGTTTGAGTTGAGATGAGTTGTCTTGACTGACTTGGATGAAAGAGAGAGAGAGAGAGAGAGAGAGAGAGAGAGAGAGAGAGAGAGAGAGGGAGGGAGGGAGGAAGGGAGGGAGACGACGAGAATTAATCGAAAAGAGGACTTTCAAC

>P779

TTCTTTTCCGAGAGAGAGAGAGAGAGAGGGAGAGAGAGAGAGAGAGAGAGAGAGAGAGAGAGAGAGAGAGAGAGAGAGTATATAGAATTGGGTGGGCATGGAGGTGGGATCTGGGAAAAGTTGGGAGAGAAGGAAAATATGATCAAAATATATTGTATGAAAAAAATGATTAAAAATGAAAATGCAAGATATCAG

>P780

GAGGAGAGGAGAGGAGAGGAGAGGAGAGGAGAGGAGAGGAGAGGAGAGGAAAGGAAACAGGAAAGGAAACAGGAAAGGAAAGGAAAGGAAAGGAAAGGAAAGGAAAGGAAAGGAAAGGAAAGGAAAGGAAAGGAAAGGAAAGGAAAGGAAAGGAAAGGAAAGGAAAGGAAAGGAAAGGAA

>P781

AAGAAGGAGGAGTAGGAGAAGAGGAGAAGGAGGAAGAGGAAGAAGGAATAGACTTAAATGTGAATAGCAACCAAGATTAAATCAGAGAGAGCAGGGGGAGAGTGG

>P782

TATTTTCCCCGACGAACCAGGGAGGAGCGAGAGAGAACGAGAGAGGAGAACAAGGACGAGGGAATGGGAGAAGGGTGAGGAGAGAGAGAGAGAGAGAGAGAGGGGAGGGAGGGAGGAAGAGTTGAGGAGAGGGGTGTGGGACTTATTTGAGACAAGATATGATATGTCACCACGTCGGAGTCTCCCCCTTCCCTTAGGGG

>P783

AAAATATATAAATGAATCAATAACCTATAAAATACATAAATGTAAAACTTACGATAGGAGAAAGACCCACAGGGGATACTCAGGAAGATAGGGTAGAGGGGAGGGATACGAAGATACTCCCACGAGAGGGTATGTGGGTGTGTGAGGGTGGAGTGACGGGACTGTAAGGGGATGAGACTT

>P784

TTTGGAGTTGGAGGGAGTTGGAGGGAGCAGGGAAGGACTGCTTAGATGATGTTAATATAATCTCATTATTATAACATACCCGAAAGCCAAAGAGAACCACACAACCATAACTGGGTTTCA

>P785

CTACCAGACCTGGAGAAACCGATATGCCTCTAGACACCCTTTTATTTTGGTGGTTCCGAGGACCACTCGGTGTTCGGACTCTGTGGGGGTGGGGGTGAAAGAGTA

>P786

TTTTTAACCCATAAATAAAGTAAATGTAAAGGTTACGATAGGGTTTTCAGGGTGTGTGTGAGGGGGTGGGTGAGGGGGTGGGTGGGTGAGAGTGAAGAACCGGGA

>P787

GAGGAGGGTACGGAAGTATGGAAGAGAGAGAGAGAGAAAGAGAGAGGAGGCTGGTCAGGGACACATGGTGAAGAGGGAGGGAAGCTAGGAGATGAATGAAGAGACACACAGAGAGAACTGGAGTACCTGAGAGTTTTTCTTATATGCCAA

>P788

GGGGGGGGTACTGGTTAGTTCATATTGTTGTTCCACCTATAGGGTTGCAGATCCCCCCAG

>P789

AGTCACTTAGGGAGAGAGGGAGGGAGGGAGAGAGGGAGGGACAGA

>P790

AGCTGAGGACAAAGCGGCAGCTGCCAGCGGGGTGGGGTGGGGGCCCACAGCCTTTGTGCTTGTGGGGCCCCTCCTGGTGACCTCACCATCTACCAGAGAAGGGCCCTTTCTTCCCAAAGG

>P791

GTTGTAGTACATCGGGGATACACCCACGGGGTCTCGATACCCCTGGGAAGGAGGGTGGGAGAACAGGAGTATCGGGGGTAAAGTGTCCCCATGTTCTCGAAAGGT

>P792*

AGAGAGAGACAGAGAGAGAGAGAGAGACAGAGAGAGAGACAGAGAGACAGAGACAGAGACAGAGAGACAGAGAGAGAAAGAGACACAGAGAGAGAAACAGAGAGAGGGAGAGGAGAGAGAGAGAGAACAGCATAAGTCAATGACATAAAATTATGAGCATTTGAAACTGAGTATTAAAAATGTCATTTTGGGCCTTGAAA

>P793

AGGGTGTCCTTCCGGAGGGTCGCAAAGGTCCGTGGTCTGACCTGGTTCCTGAGAGTGGGAGCCCCCTTTGGTGGGTCGTGAAAAGGGAGGACGGGGAAGAAGGAAGTCGGGTCTCGACTGGTTCGACGGGGGCCCCGGGGGGTCGAGCAAGAGTCGAGAAGACGCTGTGTCGCCACAGACCCTACAGACTCTAACTCTTT

>P794

TGGCTAGGTGGAGGCAGGGAGCACACTGACAGGGGTGGGGCAGAACCTGGCACAAACACGAGCTCACCCCATCTGATGCTCTAGGCAGGCCAGCTCCGCACGGGCCTTTTCTTCCAGGGCCAGCTCACGCAGTCG

>P795

AAACTTTTGTTACAAAAGTTGTACTGGATGGTTGACTGTTACAATGTACATCCGAATTACCCCAGGACGTAGGCGAGGTAGTATCCCGGAGGGTGGGGAGAGTTTTTATAATTGGGTCTT

>P796

GGGTATACCGTGACTTCTTCCCTTCTAGACGAAGCGAGGTCCTTCTCACCGGTCTCCCCACAACACCTAAACCTGTGAACGGGTGGAAGGGGTACGTGGGGGATACGATGCTGGGAGGGGGGTGGAGGGGTGGCT

>P797

GCGAGCCCTATCTGAGGGATGGGGGAGGGGGTGATGATGAAAATGTGTGCACATGGTGGGGCTGACTGAGTTATACTTACTGAACTTATAATTAAGCTAACAAGGCATAACCTTCTGAGCCTTACATGTACACAG

>P798

AGGCTGTAACTGGTGTCCAGTTACAGCACTAGGGGTGAGAATGAGGGGGAGAGACAGAAGGAGAGGGAAGGAGGGAAGGAGGGAGAGACAGAAAGAGAGAGAGGGGGGGAGAGTCAGTCTTGGTTTTCTGGCAGGCATGGGCTGACAAGGTTTTTAAGCCATTTA

>P799

CAAGGTAGTAAGTGGGCAAGGTTTGCATGAGGGCAGGTAGTCTATCAGAAGCCTATCAGG

>P800*

TTCCTCTACTTTCTGTTGTATGTGGAGAGAGAGAGAGAGGGAGAGAGAGAGAGAGAGAGAGAGAGAGAGAGAGAGAGTGTGTGTGTGTGTGTGTGTGTGTGTGTG

>P801

TTTCCTTCTTCGACAGAAGTATTGTTTGTTTCTGTTGAACTAAAAAGAAAGGAAAAGAAACGGAAAGGGAAAAAAGGAAAGGAAAGGAAAGGAAAGGAAAGGAAAGGAAAGGAAAGGAAA

>P802

TGGGTCTCAGCGGGAGGTTGGGGCTATGGGTCTCCTCAAGTTTCCTCAGATGATTCAAGACCATCCTAGTTAATCTTTATTATAAGTGCAGTCTCTATAAAATACATTTGCTGTGGATAAGGGATGCATATGCCA

>P803*

GATGCAACTCAAAAGTTTGAAAAAAGAAGAACACACCAAATCTAGGCACAGTCAATGTCAAGAAGTGATAAAAAT

>P804

AGAAAGGAGGTAGCGAAGAGGGGCTTGTGGGAGGGGAGAGTAAAGGGGAATAGTTACATGATCTCAGCCCCCAGCCCACTCCAGCTGCAGTCTGTAAGTCGGAGGCCCCTCCCCTTGGTT

>P805

CGTGGGTGGAGATGGGGGTGCTGGGACTGAGGAACCGAGGCCCCGGGGACAGATGGATTCTAGGAGCTTGCTGGCCAGCCAGCCAGTCAGTCCAGCTAGAATAGCGAGGGAGCTTCAGGT

>P806*

AGGGTGTTGTGGACCGTCATCGGTTATAGAGAAAACGTTTCATAC

>P807

GAGGGTAAAAACAGAAAGTAGACATCGTCGGGCCACTCTGTCCACTATATAGTCTTACCTTCTGAGTACTTTTAACGTAGATGGGGAGGGGGTGAGTGTGGGGAGGGAGTGGAGGTTCGTAGAATTAAAACCTGG

>P808*

GAAGCCCGTCTTATCTGCACTGTTGCTGGG

>P809

AGATAGTAGTGGCTCATGTGTAAAGGGAGGGGGGAAGCTGTTGCCTAGGACAGAAACCTTTGGATTGAATGCTTTTCTGACTGGGAAAGATCCTAGCTGAAAATATGTTAATGAATCAAC

>P810

TAACCCGGAGAGTAAATCTACGTTCCTTACCCAACGTCGGTAAGAATCCCTTCCTACCTCACTCCCGGGCCCCGACGACGAGAGGAGGGACCAGGGCCAGGTGACATGGGGTGACCGAAA

>P811

GTTACAGAGATCCGAGTCGTACAGTTGTCGACACAGGAGGCGGAGTACGAGTGTGTTCGGAGGGGAGTACGAGGGTGTTCGGAGGGGAGTCCAAGTGTGTTGGGA

>P812

ACAGAGCTTTATTTCGTTTTGATAGGGTTTGTTCAGGACAGTTTTCCAACTTATAGAAATAAAAGAAGAAGGAGCCCAGAAACCGGAAGTCGGGGAATAGAGGGTGGGCATAGAGTTCAGGATATGGTGAGTGAA

>P813

AGTAAGTCATTTTCTTCTTTCTTGAAAAAATAATAAAGTAAACAAATGTAAAGTTTACAATAGGTTGAAGGGTCAGAGGGGAGGTGTTCGGGGTGGGAGATGGAGAGTGAAACGGAGATTCTTTCACGAGGGGGT

>P814

AAAAATGCAGGGCCGGGGCGGGGGAGGGGAGGGGAGGGATGGGGGACTTTTGGGATAGCATTGGAAATGTAATTGAAGAAAATACGTAATAAAAAAAAATCTAAAAAAAAAAAAAATGCAGGGCCTTTGGAGAAG

>P815

GTGGGGACAAGGAATGGAGGGTGGGTAGAAGAGATTATCCCTTGATGGGCCCATGGAACCAAGTGAAGTGCTCTG

>P816

AGAGCAGAGTGGGCGTAGGTCAAGGGGCTAGAAAACCATAGGGGAAGTTCACTGGGGTCAACTGTCCTAGCCCACCTCCAGACGGTGGGGCACACTTGCTGCCTGAGGCTCTGGCATCTGTCCTGGAGAAAGGGAGAGAAGGAGGGCAGGGCAGGGCCTCCACACCAGAAACAGCAAAATCTGGTTTCTTTCATTTATTT

>P817*

TGAGAGAGAGAGAGAGAGAGAGAGAGAGAGAGAGAGAGAGAGAGAGAGAGAGAGAGTGTGTGTGTGTGTGTGTGTGCGTGCAACAGAGAAAGAGCTATGCAGATCTTGACCTATGGACTCAGACCTTCTCCCCTC

>P818

AATGGTAGATTTCTGGGGATTGGAAGGGGTGGGATGGGGTGGGATGGGATGGGATGGGAT

>P819

GATCGGAGAGGCGGGTCCCAGGGGTGAGCCTGGAGATGGGGGGAGGATCATCGGGGGCACAGGCTCAGGGTCAGTGCAGTTGGCTTCCGGTGGAGGGCTGATAGGTGTCTCGAGGATGGG

>P820

CAATCAAGGGTGGGAGAGCCCAGCTCATTGTGGGTGGTGCCATCCCTGGGCTGGTCATCC

>P821

AACCGTGTGAGCCAAGACGGCAAACACCTAAAACTAGGGTCCTGGACCTGAATTTAGTGGAGACTAATAACCAGGGAGGGAAGGGTGGGTGAGAAGGGAACAGTAGGAACGTTGTGAGAC

>P822

GGTCGTGAACAAACTAACTCCGTCCTCCTAACACGTAAACTTCGGTTGTACCTGATATAACTTTAGGGTAGAGTTGGATGGTGTGGGGGATGTGTGGGGGGTTTTTGGGTTGTTCGGTTT

>P823

CGCCTTACAGCGACACAGGAGTCACTCTAACATCTAAGTCAAAGGAGAGCGGTTGGTCAAGTCGATCGTTGACCTTAAGGACTTTACGGGGGGGAACCATGGAATCGGAAGTAGCTTACTGAAACGGGTGGGTCCTATAGGGATAGAGAA

>P824

ATAGGAGAGGGGGGTGTGGGGGTATCTAGTATAGAATATGGAGAAATAGAAAAATATTTG

>P825

TTTGGGTCTGTGGCCATGCAGCTATGGGGGTGTGTGTTAATGTCCATGGCACATGTTACCACCAAAGGCCATGAGAACATCTGTGGCTGG

>P826

CGTAGGGACTTTCGTAACCACATGTCCCTAACGGATTGTCCGAGTTCCGGGACCCAAGTGGGGGTCGTGGAAGGGTTTGTTCTTCGTTGTTGTTCTCGTTTTATCTCTCCTAACGCTATCTGGGAAGGGAAATCCGTCTCGGTTCAGGGTGGTTGAGTCTTCCGT

>P827

CCCGTAGGTTGAAGAGTCATTTACTAAAATGGACAAAACAAACAAAATGACCAATAATATAAATAAATGTAAAGTTTACAACAGAGGGAAGAGACGGAGGGGAGGTGTTTGAGGGGCAGGGTATAGGGAGGGAAA

>P828

AGAGGAGAGGAGGGGAGGGGAGGGGAGGGGAGGGGAGGGGAGGGG

>P829

GGAGACGGGGAGGAAGAAGAGGGAGGGGTAGGGGAGGGGAAAAGGGGGAGCGAAGAGGAGGAGGAAGAGGAGGAGGAAAGCAGAGGCACGAATGAATGTTAAGGGTAGAGGAGAGCAAGCAGCCTGTGACAGGCA

>P830

AGTGGTATTGAATTGGAGGTAATCGGTGTTTTGTTAATTATTTTTGTTTCTGGACAGGATGTCAGAACAGCTACCCTATCTGGAAATTTCTGTGAGAGTGAAAGGTGGGCGGGGTGGGTTAGAGAAGAACAACGA

>P831

GAGACGAGGACAAAGTCAAGGACGGGACTGAAAGAAGTTCCTACCTAATACTATACCTTCATGTTCGACTTATTTGGGAAAGAAGGGGTTCAACGAAATCAGTATTACGAAGTGATGTAGTTGTAATTGGGGCTGCCTCTGTGGTCCGGGAGGGATTAGAATCGT

>P832

AAAGAGGAGCATATGGGAGGGTCTAGAGGGAGGATAGGAAAGAGGAATGAGGGGGTGATTCAATAAATAAAAACAATCATTGAAAAATAATAAATTAGATAGTTT

>P833

CAACCTTTTTTTTTTTCAAGGACCTTTCTGTTGAGTCTCCAATTGTCAAGAATAGTAAGTCAGTATTCCTGGTCTCAAGTCTCGGGTCGT

>P834*

TAACTTAATATTAAGAGAGAGAGAGAGAGAGAGAGAGAGAGAGAGAGAGAGAGAGACACACACACACACACACAC

>P835*

CCGGGCGTGGTGGCGCACGCCTTTAATCCC

>P836

GTGTCTTATCGGAGAACGAACAAGAACAAACCCTTTGAAACAAATGAAAATGGATTAAAATCACCGATCAAGACTCCAGAATACCAATCATAAAACGGGTGGGTTAGAAGGTCAGGGGAGGAGATGAGACTCCAGAGAAGTGGTCGGACA

>P837

AGACAGAAGGTGGGGGGGGGGGGTCTGGGAGGAAAGGAGGGAGAGGAAACCTCCAGTCACTATGTAATGTATGAGAGAAGACTAAAAAAGTCAGACCTCCTTTAT

>P838

GTGGGACTATGGTGGTTCAAGGACGAAGGGGGACATTGACGGATT

>P839

GTGTACACGTGTATATATGAACGTCATTCAACACGTATCTTTACCTTCAATGTCTAGATTTAACCTAAATTTAATCAACAGATAGCGTAGGGACGGGATGGGAGTGGTGTGAACGGTCTTAATCGAAATCTCTTT

>P840

GAATAATGATATGTACATACGTTACACAAGACCAGTATAGAGGGATATGGGATGAACATCGGGTAGAGGTTACGGGAGTGGTACCGAGGG

>P841

GAGACCATGTGAAGGGGAGACTGGGAGGAGAGGAGGGACTGCAATAGGGATGCAAAGTGAATAAATTGATTAATGGAAAGAAAGAAAGAATATGTTCTACACACAGCAATGAAAATTGGA

>P842

GGCGGGCAGGGGCGGGAGGGGAGAAGGGGGACAAGTATATGTTTGTGTGTCTTTAGATCTTATTCCACTTGGGACGCATTGCGGGGGGGGGGGGAGGAGGAGGAGGGAAGGATGGGAGAGAGAAAGGGGAGGGAGGGAGAGAGTGAGGAAGAGGAGGGAAAGAGGGGGCTGGAGAGATGGCTCAGCGGTTAAGAGCACTG

>P843

GTGGTAGAAATGATTACTCTGTCCGCTGGAATCCAAAGAACCCCAGACCGAGGGAGCCACCGTCAACAATAACGTATACTTTCGACAGTCGGTGGGGGCGGGGGATTTATGTAGAAATAC

>P844

GCTGGGGGTGGGAAGACAGCGCTGGGAGGACCAAGAGCTGCCAGAAGAGGGGACTTGAAAAAAGAGAGAGAGAATGTAAAGGCCATAGGA

>P845

GAGGGAGAGAAAGGGAGGGAGGGAGGTAGGGAGGGAGGGAGGGAGGAAGGGAGGGAGGATAGACTTTATCCCAGA

>P846

CAAAAGTACTAATATCAGTAACAGAAGAACATGACGTCAAAGAGGAAGTAGAAAGTCAAAATAGAGGAGGAAGAAAAGGAGAAAAAGAAGAAGAAGAGGAAGAGGAAGAGGAGGAGGAAGAAGAGGAGGAGGAGGAGGATGAGAAGAAGAAGAAGAGGAAGAGGAGGAGGAGGAGGAGGA

>P847

ACTAATCTATACCCGATAACTTTAACGATAGAATCATAGTGATCTATATAAATAAATCAAAGTTTTGAAGGTTGAATTGACAAATTTAATTTTTAAGTTTTTGTTGTTTAAATAACAGCAGGGGGGGGGTTAGTAAGAAATCGGTGGGAATGTTTAGTAGGCATG

>P848

TGTGTGGGGGGGTGGAGGGTCAGGCTCCCCAGATAAGGTAGATAAGAGGAAGCCCCACTGGGAAAGGGAGTCCTCCATGTTGTGCGGAGCTCCGGGGAGCTCTCCCGACATGGTAGACTT

>P849

AGGAGATAGGGAATTTTCAGAGGGGAAACGAGGAAAAGGGATAACATTTGAAATGTAAATAAAAAACATATTCAATAAAAATTTTTAAAA

>P850

CAGATTAAAAAAACGTTTGACTCTAGAACCGCTAGAGGGTGAGGGTGAACCGACGTGACTCGTGCTCCGGGGTCC

>P851

GCAACAGGCCTCTGGGGGCCCTGAAGTGGAGTGGGGGGAGGGGGACCAGAATTCCTAGGGGATGGGGAAGAGACCAGAACAGCAAGCACTAGCTAGCATCCCTCAGGGGTGTGCCACAGAAGTAGCAGCCCTTTC

>P852

ATAACCTATAAAGAAATAAATGTAAAGTTTACGATAGTGACAAGGACCAAAGGGGAGGATTTTTGTACGAAAGGGATAGGGGATGGGGGATGGTGATGGGTGGTGAGAGGGTGAGGGTGA

>P853

TAAATGTCAGGTCAGTGATAGATGGAGGATGAGACGGGAGACAAACAATAGGATATGGAGGAGAAGTACACAGGTCATCCTAAAGAGATGAGGGATGGGGTTGGGTTGATCTGAAGGGAT

>P854

GTCGATTCTCCTCTCCGATCTTGGGTGGGTTTGTCAGTTCGAAGTCAGAGAGTGCCATAGTAGTGGTGGTGGTGG

>P855

GAAGAAAAACTCTTGAGAGATAAGTCCTGGGTCCGGGTGAAAAGTTTACCTAGTAAAAAAATTAATCCATAAAAGAAGTAAATGTAAAGTTTACGATCGGGTTTTCAGGGGGTATGGGGGGTGGGGGGATGGGTGGGTGAGGGTTAAGAACCGGGACCACAAGGGGACATGATTCCGTAT

>P856

AGCAGCCAGGGTGTGGTTGGGTGATGGGGGTGGCAGGGCCCTTTTAGGAGCCACTCAGGGAGGCTCAGCAGAAACTCAGAACTAGTCTTCTTTTCTAAGAGCCGGTATGAGTTTTGTTTAAGCAGGGTCTTTTTA

>P857

CGTCAAGTGCACCTGTCGTTTTGAAAAAGTAAGAACCCAACAAAAAAAAAAAACTCTAGTATTAATATAATAAAGGGGAGAAGAGAAAGGAGGGAGATTTGACAGGTTATCTGGGGTGGAGAGAGAAAAAGTTTA

>P858

ACGGGGCAGCCGGAGGGTCGAGGGGAGGGGCGGGCGCGGGAAGGATCGCGAAGGAGCCGGGAGGCCCCGCCCCTTCCGTAGAGATCTCTA

>P859

TAGTGTCGTACACGAACTACATCCCAATCCTACTTTGTTCCATGAAAAAATTTTTATTTAAAAATAATCTATAAAAGAAGTTAATGTAAAGTTTACGATAGGGGTTTCGGGGGATATGGGAGGGGGGTGGGACGAGGGGATGGGTGGGTGAGGGTGAAGGACCTGGACCGTAAGGGGACA

>P860

AATAGGGGGTCTTGTTTCGGTGGTTGTTTTTAGTAAACCCTTTAACTTAAACTTTTACGTGGGAGACGTCGGGGTGGGGTCTGAAAGCCTTGGTCCTGAGTAGAG

>P861

CAGTGTCTGTCAACACCCACGACCCTTAGCTTGGGTCCAGGAGACCTTCTCGTCTGTAAT

>P862

ATCTGAAACTTGAAAAAGTTTCGTTTGTGTTTAAATAAATAAATAAATAAATAAATAAATAAATAAATAAATGAGTGGAATGTAGGGTGAGTGGTGAGGGGAAGACCAGTATGGGAGGATGTTGGGAGGGAGGGAGGGGGAAGGGGAGGAGGAGACTCGGAGGAACTATAGGAAGGTGGGATTGTGAAGTTCAGA

>P863*

TCGGTAGTCTGACCAAAGAAAACCACAGTCGATTTGTCTCATATTTGTAGTTCAGACAGAAATTAAATTTCCGTAGTAATACTTACCAAGAGAGAGAGAGAGAGAGAGAGAGTGTGTGTGTATATGTGTATATTT

>P864

TACCGTTCTATTAAAAAAATTGACCTATAAAAGAAATAAATGTAAAGTTTACAAAAGAGGAAAGGACCAGAGGTGAAGTCTTTGAGAGATAGGGTACGGGGGAGGGGGACGGAGATATTCCGACGAGGGGGTGGGTGGGTCGGGAGAGTAGAAGGGCGGGACCGT

>P865

CATGTCGATGTCACATGAGTATATAAGTATTCTATTTATTTATTTAGAAATTTTGTATTTTCTTCTGACCCCGAAGACTTCGTTTCGAGGGGTTTGACGGGAGTAGGGCACCTAAGGGTGGGTGGGAAAGGAAGTGTGTGAGACGATAAATAGGTGAGTTGTAGG

>P866

AATGTAAAGGTTACGATATGGTTTTCAGGGGGTATAGGTGGGTGGTGGTGAGGGGACGGGTGGGTGAGGGGGAAAAACCGGGACCACAAG

>P867

TTTGTCTGACAATCGAAGATCACTTTATCTTTGTCACTGATTTTCAAGAGATGGTATGTGGGGGGGGGTTTCACGGATTTCACTCTAATGAAATCGAGTATTTAT

>P868

TAAGGAGTAAGAAGGGAAAGCTGGAGGGTGGGGCTGGGCGGGGCGAGTGAGCAATAGCTTGTTCAGAGGATGCCACCGGGGAGTAACAGGCAGTAATTAATAACATGGCGGTCAGAGTCACAGCCCTAGTGTTAA

>P869

CTCCATAGTAACTAAGAGGTCTCGAGGGATTCCCTAGTCCGACGTCGAAACGGACACGAAAAAGACAACCGACAGGACGTAGTAGAGAGAGAGAGAGAGAGAGAGAGAGAGAGAGAGAGA

>P870

CCTGATCTGGTATTGGGTGAGGGAAAAGGACTGAAGCCCAGAGGGCCAGCAAAAGACTATGGAAGCAGGATACTTCAGGAAGTAGGAGGGTGGGGGAACCCTCCAAAATGCCCAAGAGATCTGGGAGCTAAGAGACTCTCAGGAATCAAAGGGAGGAACCTTAGATGAAATGCCCAACAGTGGGGAGAGGAAACTTACAG

>P871

CGAATGAGATGGTTCGGGGTAGGGAGGTGGGGGACCTTAGCACATGTCAGGGCAGGGCAGGGCTGGCCATGTGGTGGTGTTGTTGAAGCTGGAGAGTGTGAAGGGAACGCAGCAGAGCTGTGGGGCAGGCAGTGAGGGAAGGCCCAGGACAGTGGGAAAGGTTCCAGACCCAAGGGAGGTTTGTAAGACTTGAGCTCTGA

>P872

TCAGGGGGCTCTCCCCTTGCTTTTGGGTTAGGGATGGGGCATGTGTCCACTTCTCCTTTCAGCTCTAAGACTCCATGTGGTACAGACCCACGCAGGCCCTGTGCATGCTGCCTCAGCCTCTAGCAGTTCATTTGC

>P873

TAGTGTGGGACACCCCATACCCCATCCCCACTCCTACTGTTAATAGTCCGTCGGTAAGAGACGAGGTCGACCTCGGATGGTAGGGGAGTGGGGTGAGACGGTCGGTACAAGTGTCGTGGCAGGACTGTGTTGAAAGGGTACTCAGATGTCCGACTTTAGGGTAACGAGAATACCGGGTAGGTCTAAGGTCGGTAGAGAAG

>P874

GTACCTTTATCTTCAGAATAGATAGAAAAATTAAAAAAGAACCTATAAAATAAATGTAAAGTTTACAATAGTGGAAAGGTCCAGGGGGGGGGCTTTTGGTGATAGGGTGGGGGGGGGAGGGACGGGACCGTAACGGGATGTGATCCCATA

>P875

AGGACTGGTTCATCCAAAATAAGTAAAATAAGTAAATGTAAAGGTTACGATAGGGTTTTCAGGGGGTATGGGAGGGGGGGGGTGAGGGGATGGGTGGGTGAGGGT

>P876

AATCCCACCAATAGTCCTGGGGTGGGGAGAGGGAGAGGGAGAGGGAGAGGGAGAGGGAGA

>P877

TTCAATAAGGCGGTCACTCTTATAACTTTTATGAAATGAAAACCGTTTCACTAAAAATTTTTTATAAACTTCTACGTTGAAAAAGTTTGGGTGTATTTATACAGGGAGGGAGGGACCTATTAGTTCTTCCATAAA

>P878*

AGGGTTGGGAGGGGAGAATTTTGATCTAGTTTTAGCTTTGTTTCTTCTGAGGTGTTTGGATGAACCAGTATTGACGGTTAATAGTCTCTC

>P879

CCTGGGCAAGGATTCTGCATGGAGTTTGGGGGGGGGGTGTCTCTGAGTTTGATCTTCTTGAGTTCAGAGATATCTAGGGGCAAAATTGCTGCCAGCCCAGAGGGC

>P880

CTGTGTTTAAGACGTAACGATTCAATTGACAGAATAAAAAATTAGTTTACTTCCGAAAATAAAAAGGAATGGGATAAAAATTATTACCAAGAATTTACGAATTGGAGGGAAGATCGGGAGGTGGGTGGTCTTCATCACCTTTTCTCTCCA

>P881

AAGAGTCTCCTTTCTTCCACGACGGACGACACGAGAACACACTCTCCGTTGACCCCCACCCCACGAACAGCCGTCAAGGGTAGGGTCTGAGGAGAGGGGTGGGGA

>P882

TCATCATGGGTGTTGGGGGTCACTGGGGGCAAGCAACCCAGACACTGCTGGACCACCTCTGCTCTGTTGCTAGCTTGTCCGTCTGCCTGTTGTCACCGTCCCCGGGGCTTCCCTCTGTCTGGGTTTCCCTGAGGCAGGTCTAGTCACAGGCTCTGGGGCGGGTGGCTCACCAGTCTGGGTGGTGGGGACAGAGAGAGTCA

>P883

AGAGAGAGAGAGAGAGAGAGAGTGTGTGTGTGTGTGTGTGTGTGTGTGTGGGAAGAAAGGGGTAAGGAGAGGGGGAGAGGGAGAGAGAGGTGTCCAAGGACTGACCGGAGAGGGGGGTGGGGGTCAGAGAGGAGAGAAAGAGAGAGACGGAAAGAGAGAGAGATGAGGGAGGAGTTGAGGGGAGGGGTACGGGATTTATC

>P884

TCCCAAATGTACCCTTCCAGACTCTTTCTTTTCCCTTGGCGGGTGGAGTGGAGTACTGTACCTGACGAACGAATGATGTGACGTTAGCGAATGAACCAGAGAGAGAGAGAGAGAGAGAGAATGTGTGTGTGTGTG

>P885

CTTGCTGCATTCCATTGTTAAGTCACAGAACAGGGGTGGCGTGGAGGGGAGGAACTCATTATAATCAGAAGACAGCAATGTGGAATGCAGCAGCAGGCAGCAACTGAGCATTTGACAGAA

>P886

CAAAATATATGTATATATAAAAAAGATTAAAATTCTATCTGTTTTGGATGTTATAAGGTCTAATTATTGACTGTGACAGTAAACACGAGGGGGGCGGGGCACACACACACTCGCACTCAC

>P887

GGGAGGGTATAGGGAACTTTCAGGATAGCATTTGAAATGTATATAAAAATATCTAATAAA

>P888

ACTCACAGGAAAAACAAGATCTCGAAAGTACACATTCAAAGACAATTCAATGTACGTACTCTGGAGAGGAAAGAAAGGAAGGAAGAAAGAAAGAAAGGAAGGAGGGAAGGAGGGAGGGAGGAAGGGAGACAGAAA

>P889

TCATGGGGATGAGGGGGGAGGTGGGCAGGTTGGGGTTCTGGGAGGATGGCAGTGTTAGTTTGGGGGTGTACTGTCTGATTTTGTGTGTCAACTTGACACAGCTGGAGTTATCACAGTGTA

>P890

ACGATGTTGATTGTAATAAGAATTACTTTTCAACACTCAACTACCAAAGATGTAGTATCTGACCGTTACCAGGACACTTACCATTCGGTCACGGGGTGGGGTGGG

>P891

ACAAATCTCTACAGAGATGTAACGGGCAGGGACTACAAATCTGTACAGAGATGTAATGGGCACGCCGACAGGAGCCAGGGCCTGAGCATCAGTGTTGGCGGTTGATGACCGCAGTCCTCA

>P892

AAGGTGGAAGGGAAAGTAGGGAGAGAAAGGAAGGAGAAATGGGAGAGGGAGAGGACTCAGAAAAGGAGGAAGAAAAGCAGAGCAGAAGCACATGGCCTGGAAAAACTGCAAGTTCTAAGG

>P893

TCTCCCTCTCGCCTCGGACCCGATGGGTCGACCCCCAGCTCCGCTGTCGTGGGCCACGTGGATGGGCCTTCTACCTCCACCGCGGAGGCGGTGGGGGCGGGGGCGGCCGGGTCGCAGAGG

>P894

CCAGATGGACGACTGACATGTGTAGGGTATGGAGGAGACGTAAGAGGTACTCCTACAGGGGTGGGAGTGGGGTTGAGGGTGGGTTGGTCTGGAGGGGTGAGGTAC

>P895

GTAGTGAGTTGGCAAACGTGGTTCTTGTGGCCCTCCCCCGTACACTACCCTCGAGAAAAGGGTCTGGTCACAAACCGAATCATATCCAATCTGTGGGGGGTTGGGGGGTCCTAGTGTAGTCAAAACGGGAGAGGGGGTAGACGACCGGGGTTGAGGGACGTCGTGGTAGGGTACAACTCGAATTGTTTGTCAAGA

>P896*

CATAGCTACTACAACATTCAGAAAGTAAATGATACAATGTTACCC

>P897

ACTATAGCTGGGAAGGGGCATGAAGGGTGGGGATCTGGAAGGGAAGTTCCTTCGTGTCCTGGAACAGGTTTGGCTTCCCTTGGGAGCCACAGTCTCCTCTCCTAACCCTGGGAATGTTTTCAGGGCACACACTTG

>P898

TTATGCTCAAGCTGCTTAAAAATTCTTCAATATCTAGGGGGTGGGGTGCGAGCCTTTAGTCCCAGCACTCAGGAAGCAGAGTCAGACAGACCTCTAAGAGTTCAAGCATCCTGGTTCTTC

>P899

CTGGGGCCGGGCGGTGGTGGCGCACACCTTTAATTCCAGGTCTTGGGAGGCAGAGGCAGGTGGATTTCTGAGTTCGAGGCCAGCCTGATCTACAAAGTGAGTTTCAGGACAGCCAGGGCT

>P900

AACCCTTCCTAATTTTCCATACCGAAACAACCTCATCCACTCAGTGACCTCCATCCGAGGCTTCAAAGTTTTCGGGTCCCATCCGAGTCAGGGTGGCGGTGGGAC

>P901

CGGGGGTGATAAGCGGGGGAAGAGGGTAGTTGCGGTGGCTGAGGCTGAGGTGGGGTGGACAGTGCACACAGGATTGAAAGGACAGAACCACCACCCTCTAGTTTTGTGGTCCAGGGTCTTGGGGAAGCTCTTCACCTCCGAATAGCCCTATGGACAGAAGTTTAAACATCCTCTTCTTCCTTAATTCAGCACTATGACTC

>P902

CAATGGGACGTCATCCTTTGAACACAATTTGGTAGAGTCACCCGGTGGGACTTCCTGCCATCCGTGCGGGGGTGGGGGTGGGGGATTGGGGGGTCGTTAGAGGTA

>P903

TGGATAGGGGAGTGGGGGGAAGGGTATGGGGGACTTTTGGGATAGCATTGGAAATGTAATTGAGGAAAATATGTAATAAAAAATATTTTTAAAAATACCAATAAGGAAACGGGGATGACGGAGTAAAGCAGCTGA

>P904

TTATTTTTTCTCAACGGAACCAGTACCACACACAAGTGTCGTCACTTTGGGATTAATTCGGGGGGGGGAATTAAA

>P905

TGATACCTTTTTATATATTAGACTTTAATATTATATTTCATTTGTTATTGAATCATGAAATAAAAAATTTAATAAATAAATAAATTTGAGGTATAAAATGGGGGAGTGGGTAGGTGGGAGGTTGACATAGGGTAGGGTATGGAGGAGGGTTGTGGACACAGATGTGTACCTACAGAGGTG

>P906

AAGAGAGAGAGAGAGAGAGAGAGAGAGAGAGAAAGGTTCTTTGGAGCAAATCCTATGTGTGTTGTTTGGAAATCTGCAGTTCAGTTCACAGGTCAGCAGTGGCAG

>P907

ATTCCCGGGACAATAGAGGGATGGTGACTATTTGGACAGGAACCTCTGGGTCGATTCCGTGAAAGGGTGCCTGAGATAAATCGGATCTTGTCAGGGTTCTAGTCGTATTCTACGTACTGTCTGTCCCAGGGGGGGGGGTTTCTTAATCCAAACTCTTATCTAAAT

>P908

TGTGTATGGGGGGGGTATTCAACCTACCATGGACCAAGCCAGAAA

>P909

TCTGGCCTGCAGGTCACATTGCTACTAATGTGGGGAAGAGGAGGGAGGGGTAGGGGTAGGCAGGGGCTTGGACAGGACACTTCTAGTCATGATTCACTAACCCATGGTAAACTAGATGGAATTCGTTCATTTAGTAAGTAGAATATTCCC

>P910

CAGAGATCGGTCGATTTGACAAAGGAGGGGGTCCGGGAGTCTGGGTGGGTCGGGTTAAGGAGGTAATCGGACAAT

>P911

AAGAAATAGAGGACACAGGGTGGAAACCTCTCTACCTTTGTAAGTGCTGGAAGTCAGATACCTGCCCCTTGGGCTGGGGACTGGGGCTCCTTCTCAGGAGGAGAAGAATAGAGGGGGGAGAGGATAGGGGATTTTCGGAGGGGATAACATTTGAAATGTAAATGAAGAAAATATCTAATAATAAAAAAAGAGTATGTTTG

>P912

TCCATCCAAAACTCTAAAGTTTTCGGGTACGGTCCGGGTCGGAGGGAGGGACGGAGGGAGGGAGGGAGGGAAGGA

>P913

GTTCAGCAAGTAGAGCATTGAAAACTCAGGGTAAAACCACAAGCATTGGAGGAATAAGGAGGTGGAAACGAGTGACCCAGGGGAGGAGCTAGAAGGGAGGGAATCAGAGCCCGTACATAGGAGGAGGCTGTGGCTGGATGGGGGAACATCTTTTTGTAAACAAAATCAAGAAGAGGAAGTAGTATTCAGAAAATTACATT

>P914

ACCACGTCCGTCCTCCTTCTTCTCCTCTTC

>P915*

CACTAATGTCAACACACGATGGACCGGGTCCAGGACCTCTAAGACTCAGTCGAGAGCGAAAGTGTCGTTCGCAAAATGGGTGGCTCGGTA

>P916

GTAAGTGTTACAAGTTGAGGAACAGAGGGAAGGTACTGGTGACTGGGTCACACCGAAAGGAGATTGTCACGGGGAGGGGTCGGGGTGGGGGCGTGGGGTTCAAGGGAAAGGAATGAAGACGGTACTACAAGGACG

>P917

ATCTTTGACTTGAATTCAGAGGGCAGGACTAAAGGGACCCCAGGTCGCTTGGGTGTCGAAGACAACTCCAAGTAACGTTTAGACACAATAGGGGGGTGGGGGGTAAAATTCTTGTAAAGA

>P918

CGTTGGTTTCTGCAGCCTCTGTTTTCTGGGCTCTCGCTTGTGGGGAAGGGAGGCTCTGAGTCCTTTTCTATAGCTCTCTCAAGTCCCTGCACTCCACCATGGCTTCTTCTGGTTCTTGGG

>P919

TATTTATCAGCATAAGAGGGGTTTTTTTTTGGGGGGGGTTGGTTTTATTTACTTAGTACACATGTGAATTTTTGTTATAAAGTTCCTAACTTACTCAAGAGGGTAGACTCAAAAGTCTTTTACAACAACCCCTAG

>P920

ACATGATAATGGCTTACCAGGGGGTGGGGAGGTGACTAAAGGGGCAAGAGAGCTTGCTCTGCAAGCATGAAGTCCTGAATTCAAAGCTCCAGTACCCAAGCGAAAAGCTGGGAATGGTGT

>P921

GGCCCAGGGAGTGTGGAGGCCTGGCCAGAAGGAGGAGAGAGGAGAAATCTGTAGGAGAAGGAATAGGATGAGGAACTATAATAGGTGGCCAATGACTGGAATGCAAAAATAAAATAATAATAATAACAATAACAA

>P922

TCATCCAAAAGGAAGGGAGGGAGGGAGGGAGGGAGGACAAGAAACAAAGAAACAGGGCGTGGTGGCGCACACCTTTAATCCCAGCACTTGGGAGGCAGAGGCAGGCAGATTTCTGAGTTTGAGGCCAGTCTGGTC

>P923

CTTGTTGGGATGCCCGCTGCATTTACGCTCAGTCAAGCATGCAAAGTGGGATGGGGGAAACGGGAAGGAAGGGCCGTTGTGAGCATGTTGCCCAACAACTGAAGCTGGAGGAAAGACGGTAGCCTTGGATGTAGCCTAGTACCCAGTGACAGGGCTTTCAAAGTGATGTCTGCCCAGTCC

>P924

AGGAAGTCTTGAGATAAGATTACAGTGGAGGAATCTATTCGGGAGGAACAGGTGAGTTGTATATTCAGAGGAACGGAGAGAGTGTCCTTCATCTGTGGAGTTGTAGTAGAGGGAGGGAGGGTATGGCATATCGTGGTAGACGACGGAAGG

>P925

AGGAGGAAGAGAAGGAGGAAGAGGAGGAGGAGGAGGAGGGGGAGGAAGAGGAGAAAGGCGCAGGGGTGGGAGCTGTCGCCGGAGCTGCCACAGCAAAAGTTCTCTCCCCTCCCCCTCGCC

>P926

TCTATTTATACCAATATTTGGAGGGGGTTGGGGGGCTTGGGTGAGAGTGTTTTTTGAAACAGGACCTCATGTCACCCATGCTGGCCTCAAACTGGTTATGTAGCT

>P927

CTAATTAAGTCGTGGACGGTGTACCAACGGTACCGAGTGATAGTAGTAATAACAGAGGGTTCTCTGAGTTACTGGCACCCGATAAAAGTAAAGGTGTCAACGAAGGGGGGTGGGGCTGTAAAGAGTTCGGATCAC

>P928

TAGACTATGTTCCTGTGGGGTGTGGGGGAAGGGGGTGCCTCGGCAGGCCAATGCAAAAGAGAGAAGCCACAGGATAGTATAGTATAGAATAGTTTATTCAAGGCA

>P929

GTTTAGATAAGAACAACTAAGAAGAGGAGAGGACAAGAAAGGATAGGGGAACATCAGTGACGAATACCAGTGAAAAGGTAAAGGTACAGTAAACACAGGACCACAGGAGGGGGTGTGAGACTTGGGAAGAAATCAAAGTATCGTATAGGGGTGTGTGGGGTTGTA

>P930

GTGTTTCAGTCACTAGTTAAAAAGAAGAGATGTTCTGAAATCTCAAGTGATCTTGTTTGTCGATTTCACGGAAAGAGAGAGGAAAGGAGGGAGGAAGGGAGAGGGAAGGAGGAGAAAGAAGTAGAAGAAGGAGAGAGAGAGACAGGTACAGAGAGAGAGACAGAGAGAGAGAGAAGAGAGAGAGAGAGAGAGAGAGAGAG

>P931

GGGTGGGGGCTGGAGGGTGCATAGGCATAGCTAGATGTGCCTAGATGTGGATTCTCTGGATGTCACTGCTGTTACCCCTGCCTCTCTAACATAAGTTCAGACAAC

>P932

CGTTCTCCTTAAGGGGAGTAATCTGTCTGGTTCCGAGGGGGTTACGAAAGGGTATAATTGTTGATCAATTCCGGTTCGACGAGGCGGTTCTACCGGTCCGGTGTGGTGTGGGTACTTATT

>P933

CCATGGGAGATGGGAGGGTGGAGGGAGAGAGTGCAGGGAGAATCATCTGGGATGGGAGGTATTTAGGGGACAATGTAGAAACCTAGTGCTTCAGAAACTTCCTGGAATCTATGAGAGTGGCCCTAGTGAAGTGTCCTAGTAATTGTAATC

>P934

TACAGGGACCGCCCTTCCCGGGTGAAGTGTGTGGTCGAGGACTGCAGTCAGACCACCCGTCGGTGTGTCCCGGGTCTAGAGAATCTCCGCGAGGTTGGGGGAAACTGGGTGTCGAATGACTTCATGGGGAGTCTGTTAGTCGGGGTCGCGATCTCCGTCCTCACT

>P935*

CACGTACACAAGGTTTCTCCCCATACGACAATACAGGGTTCAAAATCTAATAAACAATCTTTCCAATTGTTCCAACGAGTAAGAAGAGAGAGAGAGAGAGAGAGAGAGAGAGAGAGAGAG

>P936

TTTGTTGCCCTAGACGGGGGTGGGGGAATTGGGGGCCCCCTGGGGCCACTCTTTTCCTGCTCTTACTTGTTGGCTACCTCTCTGACTTATACTTCTCAGGTCCGGTCTTTCTCCTTTCCGGGCATGGTGGTTCTA

>P937

AGTGAAAGGACAACGACATTATTTTGTGGTACGGGTTTTCGTTGAATTCCTTCACCCAGGGGTGACTACAGGAAGATCGTTTCAACGTGGGGTAGGGTAAAGGGGTTTAAAGGGGTTTGTCCTGGTGTTGACCCC

>P938

GCAACCTCCAGGGGGTAGGGGTGGGGTTGAATCCAAGGCTTCATGTATGCTAGGCAAGCACTCTCCCATCTCTCCTTCCTTTATAATTTGAACGATAAAAATGATAGCTTCTAGATTTGA

>P939

CTAGGAGTGCTGGGGTGGGGGTACAGGTTGGGGGCTTCTCAGAGGATTTTACTGTTATAGCTCTTTTAGATAGCA

>P940

GGAGTGGGGTGCAGACAGGTGGTGTTCTCCTGAGGAAATTTAGGGAAGGGGTAAAACCAAGATGAAAGGGAGGAAGGAGATTTGGCAAGTCCTACTTGCTTGAGTTTCCTGGCCAGCAGCCATTCCTCTAGGGTG

>P941

AAATAAACATTGATAGCTGAGATAAAATAAAATAAAATTAGAAAAAAAATGTGAGGTCTAAAATAAGGGGAGAGGCAGGTGGGAGACTGACAAGGTGTAGCGTGTGGAGGAGGGGTTGGGGTGGACGACAAAGGTGATCGTACAGGGGTTCGGAGAAGTTAGGGGTGAGGTGGTGTGGAGAGGTGAGGGACCCGGAGATC

>P942

TCGAGTCGACCTTTTGGGGACACTGTCTCTCTTTACCTCACAGAATTATAATCATAGACAGACTGTTGGAGAAGGTTCCCTTTGATGGGTGGGTGGTGGGAGTGAAGATCCACCATCTGA

>P943

AGTGGGATGTGGAGGGCACACACACAGTCACAGTGTCTGTTGATGTATCTACGACCCTGGGCACATCACCCCATCCCGGCCACCCCCAGT

>P944

GTGTGTGGGGGGGGGGGGTCTCTAGGAGAAAACTTCAGCGCAGAGCAGGGTAGCGGCAAAGTGTTCCTGTGATCGGCAACCTGCTGCTTGTTTCTTGCCCATTTG

>P945

GCCTTCATTGTTCATTCACAATTAAAAGGGTTCTGGGAGGGTTTGGAGTTAAAGCCAGCTCCCAGCGGGTGCTGGTCCTGGTAACCCATGCCCCACAACTGCCTTCCCGTTACCCTGGGCTGGCTTAGCTTTAATCAACCCCTCACAGCT

>P946

GAAGTAACAGAGGTCAGTCGTTTACAGACACACGGATGCGACACGCGGATGTGACAGACACGCGGATGTGACAGACACGCGGGTGGGACAGACACGCGGGTGGGA

>P947

CTTACCAAAATCAAATAGGTGTGGGGGGGGGGTGGGGAGGGAGGATTTGCCCAGCTTACACTTCCAAGCCATAGTCATCCCTGAGGACTTCAGGGCAGGAATTCAAGCAGGAAATACAGACCAAGCTGGCTGGCT

>P948

TTCCTGTTAACATCGTTTATGAATGTCCTAACCCTATAAAGACTCCTACAATGTTACTCTGGACATCGTTTTGGAAGGGGACCGACGGACCGAGGTTTGTAGGGG

>P949

CGAACCCAGAGGACCGTTATTCTTCAGAGAGCGAGTGAAGGACAGGTCTAAAGAGGCCTAAAATGAAGAGGGAAGGATCTGAGGTTCCTAGGGAGCGGAGAAGAAAGAAGAGAAGGAAAAACGTCTCGACTCCCAACTTGGGTCCCGTGATCCGTACGACCCTTTCGTGGGACGGAGACTCGAAAGGGGGAGGGGTCCAA

>P950

TAAGGCAGGAGTGGGTGGGTAGGTGGGGGAGCACCTCTAGAGGATGGGGGATTTGCAGAGGGGAGAACAGGAAGGGGGAAAACATTTGAAATGTAAATAAATAAAATAACCAATAAAAAAGAGAGAAAGATTATATAATCTCCAAAAAAAAAAAAAAAAAGTCATAGGAGAGCTGAGCCT

>P951*

TATATATCTATACTAGACAAATGTATATCTCAGTTCTGTACTTGTATTTGTGGTATTTGA

>P952

GAAACAGGGGTGTGGGAGAGAGTGAGTGAGAGAGAGAGTAAGAGAAAGAGGGGGAAGAGAAGAAGGAGAGAAGGAGGAGGAAGAGAAAGAGGAAGAGGAAGAAGAGGAGCGAGAGGAAGAAGAGGAAGAAGAAGGAGAGGAAGAAGAAGAGGAGGAGGAAGAAGAGAGGTCAAAAGAGAAATCTGCTACCTCCCACATTG

>P953

TAGATAGGTCATGGTGGGAGAGGAGGGGGAGGGGGAGGGGAGGGGTAGGGGGAGGGAGAGGGGAAAAGGGAGGAGAGGGGAAAGGTGGAAAGGGGGAGAAGGCGAGGGAGACACACAGAGAGAGACAGAAAAAGAGACACAGAGAGACGGAGAGAGATACAGAGACAGCGACGTAGAGAC

>P954

AGGGGTACCGGGCAAAGGACAAAGAGGAGTCGTGGAGGACAAGAACGACAGAGGGATCGCGGGAACGAGGGGGGGAAACGGAAGGAAGAGATCGTAGGGGGAGGAGGGTCAGGAGTGTAAACAGACTAAGTGTCGTCTGTCGGGCAACCATGCGAGTGGTCGTCGGATTTTCGTCACCCCGGACACGACCGGGTCAGGAC

>P955*

TATGCACACACACACACAGAGAGAGAGAGAGAGAGAGAGAGAGAGAGAGAGAGAGAGACAGAGACAGAGACAGAGACAGAGACAGAGACAGACAGAGACAGAGACAGAGACAGAGAGAAAGAGAAACAAAAAGACAGAGAAACAAAAAGACAGGGACAGAGAGGCAGAGAGTTTTTGTCC

>P956

TGTGTACAAATGAGACCTTCGTGGATTCTACCTAGGGGGCGGGGGTTGAGTGGCATCTGAGCCAAGTCCAGAGGTCTGTCTTAGGGTCTGAGTGATCTTCCTGTGCCGGTGTGGTGTGGTGTGGTGGGGTGGGGTGTGGTGTGGTGTGGTGTGTGTGTGTGTGTCACTCATGGTTGTCACATGGGACATAGCTGCTTGTC

>P957

GAGTTCCCAGTTTTTATAGGCCCTCAGGGGGGAAAGTAGAAGGGGGAGATTAGGGGATTTCCAGATCTAAACAATGTCTATTCTCAAGAAATGGGTATGGGAGGG

>P958

CCGTATCTCTTCTTTCGTACCAGTTTCTTGTAACAGAAGGGAGAAAATCCCCGAGTGGTCCTACTGGAGGGGGGTGGGACAGGGGTCCAA

>P959

ACCGATAATTTTATGTAATCTGAAAACACTTAACCGACGACAATAATGTCAATCTGAATTCTAGGCTAGGGGGGGGGGTCGAAAAGGGGGAGGGGAATGGGGGAGGTGTGACTTCTCGTA

>P960

TGAGCGGGAAGCTCCACCCCTGAGCAAGCAGGTTTCAGGCTGGGGGAGGGGCAACTACACACATCCATACATGTACCATATACATATGTACATGCACACACACGCATGAACAACCACATACAGCCACATAGCCAC

>P961

AGGGGGAGGGGGAGGAAGAGGAGAATATTAGCCAGGAAGGAAAATCTGTAGAGAAGGACC

>P962

GAGGGTCTAAGGGAGGAGGTGAGGGAGGAGGTCTATTTGGAAGGGATCTTCAGCCAAAGTCAGTTGAGGGAGGAG

>P963

CAAGTTCTGCTTGTTGGAGCTGAGGTCCTCTGGGAGGGAAGGCATGGAAGGTAGGGCTGATGAAAATGAGGCATCGAGGTTCTCAACCTGTGGGTCATGACCATTGAAACACCATATTTCTGATGGTATTAGAAT

>P964

CGCACACTGGGCCTTCGAGTCCTCAATGTGAGCCCCCGTCCTCACCCGTCACAGTCGGATGAGACTAACCGACTCTCTCCCTTCCTGGGGTCTGGTGGTCGGGGTACCCCTTCTCGGGATAGTCTGGGTGGACGAATTGGTTCTTCTCTCCTGGTATCCTTCAGGTTGGGAAAGACCCAGGAAGAGGGTCGGGAC

>P965

TTGCAAGAGTAGTAAGTGGGGGAGGGGATGGGGGACTTTTGGGATAGCATTGGAAATGTAAATGAAGAAAATACC

>P966*

CATGTGGGACAAAGTGGAAGTGGGTTAAGTTAAAATGTGACCCATGAAATATTTCCTGTT

>P967

TACAACATCTGCTGGGCAGATACCATCTGCAGATAATGACAGGGCAGGGGTGAAGGGTAGAAAGGCCAAGTTGGAGAAGAGAGATGAGGGTGGGGGAAGGAGGGAGCTGTTGCAAGAACTGTTGCCTCCCTTCGCCACTCTGGCCCTCTCTTTGGCAGCCTAGGGGTGCGTTTCTCTGTTACTCCACGACTCCTTCCTCT

>P968

GAAGGGAAGGGAAGGGAAGGGAAGGGAAAAGGAAGGAAGGAAACCACTTAGGTTAGATATTCTGTAAAAAAGGACCCATTTTATACTCAAGGTAATAAGAGTAAA

>P969

TACAGGGGAATACCAGGGCCAGAAAGTGGGAGTGGGTGGGTAGGGGAGCAGGGCAGGAGGAGGGTATAGGGAACTTTGGGGAAAGCATTTGAAATGTAAATCAAGAAAAATCTAATAAAAAATTTAAAAAAAAAACCTTTAGGCTATCATAAGCTGCACTGCTCT

>P970

TACCAGGGGCAGGGAGGCAGGAGTGGGTAGGTCGGTGTGTGTGGGAGCACCTTCATAGAAACAGGGAGAGGGATGATGGGATAGGGGTCTTCAGAGAGGAAACCGGAAAGGGGATAACATTTGAAATGTAAATAAATAAAATATCCAATAAAAAAATCGAGGAAAACTTTCTAAATAAAGGCTGCAGATTTATTA

>P971

TGGGTATGGGGGACCTTTGGGATAGCATTGAAAATGTAAACGAAAATACCTAATTAAAAAAAATTAAAAAAAAAA

>P972

CCACTGTCTCACAACCATCAGTTACTCCAGGTTTTTTTTTGGGGGGGGGGGGATACAAGTCCCTCTTTTGGTTGGCAATTAATGAGCTAATTAACTAACAAGTCTTCACTTATTTAGCTCAAGTCAGGGTGGTTTGTGGAATGGATGGCCCAAGAAATTGAAGGG

>P973

GAAGGGAGGTAGAGCAGAGGGACTATGAGAAGTGGAGGGAGGGGAAACTGTGTTTGGGATTTAATGGATGAGAGAAATATAAATAAAATTAAATAAGTAAATAAGTAAGTAAGTAAGTAA

>P974

TAGATGGATGGATGGAGGGGTGGGTGGGTG

>P975

GGTGACACATTCTGGTAATCTTGGAGGGGGAGGTGGAGACAGGAACATCTTTGGCACTTACTGACCAGCCCGCCTAAACCTAATCAGTAAGCCCTAGACCCCAAT

>P976

GGGGAACGCCAGGGCCAAAAAAATGGGAATGGTGGGTAGGGAAGTGGGGGGAGGGTATGGGGGACTTTTGGGATAGCATTGGAAATGTAATTGAGGAAAATATGTAATAAAAAATATTAAAAGAAAAGTTAAGCAAGAGATCATTAAAAA

>P977

ACGACGTCAATAAACAATAGTATTTCTCATTTTTACTGACTTCAAAGACTGAGAGAAGAGACCATGTCCTTGAGGGAGGAGGAGGGGGAGGGGGTGAATAGACGT

>P978*

CATGTCCAACCATGTCCAACTTATTACATAGATGCTAGAGATTTT

>P979

GGTACTATGGTCCCTTGAGATCGTTGTAGAATGGGAAGACGACAGGGACAAGGTTTTTTCCAGGGGTGAGAGAGGACGAAGGAGAGAAGAAGGTAGGTTGGACCTTCAGGGTGGATGAGT

>P980

GAGAGAGAGAGAGAGAGAGAGAGAGAGAGAGAGAGGCCCAAGGCGTTCCCCTCTGTTCTAAGGTTGGAACTCATGTCTAAGGTCTGAGATACTGTGGTATAATTA

>P981

AGGGAAAGAGTGTGGAGGTCGGGAGGGTCTGACTTTTATTTGGGCTGTGACTTAAGTGCTCACAGGTAAAGTTGGGAGGTGAGTCAAGTTAACTCTGGGAATGTATTGCCAGAGCAGCAGATGTGTGTGTCTCTG

>P982

GGAACTTTCATGTCTCTAGGTACACACAGAAGGAAGACTTACGACCCTAATTTTCACACGTAGTTCGGACCGGTTCTGTGTGTGTGGTGGTGGTGGTGGTGGGTGGGTGGGAAATTTTAAGGACATCGTTCCACT

>P983*

TTCAGGCTTTTTTGTAGCTTTCACACCGTC

>P984

AGGGAGACAGTGAAGCCAAGTGGGGATTTGGGTGGGGGGCAAATCTGGTACTGAAACAGAGGGGCTGTTTAGGTAGGGTACTTTCAGGTAGGGTCAGAATGTGACCCTATCATTCCTTGC

>P985*

TTTTAATGGGAATATCATTGAATGTGTAGATTGATTTGGGTGGGATAGCCATTTCTTATATTAATTCTACCAATCAAATGACCCTGAGAA

>P986

TATTGGAAGTGATCCAGATTAGACTTAGAGTAAAATTTTTTAAAAATAATCTATAAAAGAAGTAAATGTAAAGTTTACGATAAGGTTTTCAGGGGATATGGGAGGGACGGGACGAGGGGATGGGTGGGTGAGGGTGAAGAACCGGGACCGTAAGGGGACATGACC

>P987

ACGGAAACAAGGCTCATCCAGGGACTAGGGTGTCTGGGGGTGGGGAGGGCAGCAAGGCAGGGCTGGCTGAGTATGGAGACATGGTTTGATTGACACTTTTGTGAGAGGTTTTCTGCCTTATTCCTGATATTGTTT

>P988

TAGTTGAAATGTTATTCTCCCTCTTTTGATCCCCCTAAACGTTTCCCCACCCGCCCAACTCCGAGGGAGGCTCCACCCCCTAACTCCGAGGGAGGGAGGCGTAGAGAGGAAAGGACCTTGTCTCAGGCCTTAGGG

>P989*

AAAGGTAGGGACGTCAAGAAAGTCTGTCATTGTTAATACTCAGTCTCAAAACTGGTACCCTCCCGTTAGGGGAGGGAGTGAACTACGAGA

>P990

CGATTCCCAGAAGTTGTCACCGACCTCCCTGGAACTCAGTGCGCCTGTCTGTTCGGATTCCACTAGCTCCTGGTACCATCTCCCTCGCGTCTCCAGTGGAGTTGTGGGGATGAGGTCTTCGGGGGTCGGGCGCGAGAGCCCGGGGTTTCAGGAGTGGCCTCGTTG

>P991

TCAAATTACATGGGTGGGGTGAGGAGTTGAGGGGAGTGCTCCAGCCTGGCCCCACTTCCAGATCTCTCTGCTCCATGTTTGGAGTTGCAGATGTGATTCTCAGTTTCCTGTTCTTGCTGC

>P992

TCGAGACGAGTCATCGACGGAAGGGTCCCGTACCGATGGAAGGAGGAGAGACGAACGGTCGGGTCTAGGTAGGGATGGGTAGGGACGGGT

>P993

CAGGGGTAGGTATGGGTTTTCCGCCCAAATAAGGGGAAAGAAGGGAGAGCAACGGAGAAATAATGACGCGGACAGTAACAGGAGTCGCGTTAGAACGGAGTTGAACCACGTTCAGCCTTTCCAAACACCCTTAGA

>P994

GGTGGATGTGGGTATGTTTGGGGAGAGTGGCGCTAGGGTAGGGCCCTTCCCTTTGTACTCATGTTGCTGTCTCCTCTGCAAGGCTGCATTGATTCGGAGGGATGCTGGAAAGAGGAAAGGTAGATAAGAATTGTT

>P995

AGGGGTGGGGGTTCTCCTGTATCCAGTCCTTCAGCATCTGCCCTGGTGTTATCATCGCAGCAACAGGAGGTACTTTGCTCTCTGTTCCTT

>P996

AAGGTAAATTTAGTAAAAACAAAACGAAAATTCTTCCCCAGGACACAACGGGACCCACCGGAGTGAACTTAAAAACTAGGGAGAGGGAGACGGAGGGTTCACGACCAAAACACCATGTCCCATTGTTACAAATTT

>P997

AATGAAGTTTGCATCGGACCACTCTCGTTATTTAAAATAAAGATGAGAGAGCACAGGGATGTCTATTTTAGGTGGGTGGAATGATACCTGCCTGGTACAGGGCGGGTGGTAGTCTGAGTGGGATAAGACCGGGTTCGGTCGACCCGATTG

>P998

AGGGAAGAAGAAGAAGGAGGAGGAGGAGGAGGAGGAGGAGGAGGAGGAGGAGGAGGAGGA

>P999

AGATAGATGGAACGTTTGTTGAACCTCCTCCCACTACTGGTTTCGACTGACGACAGGCAAAAACCTCCGCGGGTGTCTGACGGGTCAGGGTGGGGTCCACGGAGA

>P1000

GAACCATAGACAAAATTATAAAATTATCAAAATCGAACTTGTACAGTATGTACATGTATTATCCAAACCTTGTTTAAGTGGGTGATAAGGAAGGGGAGGTTCAGAAAGAGAGAGAGGGGTGTTGATGTTAAGGGA
