# Supplementary material for: QUFIND: tool for comparative prediction and mining of G4 quadruplexes overlapping with CpG islands
Source: Front Genet. 2023 Oct 25;14:1265808. doi: 10.3389/fgene.2023.1265808 (PMC10634401; doi:10.3389/fgene.2023.1265808)
Supplement: Supplementary file 2 [file Table2.docx]

Supplementary table S2 displays the experimentally validated G4s mined from the literature.

* Denotes sequence with no predicted quadruplexes.

| **Sequence ID** | **Quadruplex** | **Ref** |
| --- | --- | --- |
| **Seq1** | AGGGTTAGGGTTAGGGTTAGGG | [1] |
| **Seq2** | AGGGCGGTGTGGGAATAGGGAA | [1] |
| **Seq3** | GTTGGGGTTGGGGTTGGGGTTGGGG | [1] |
| **Seq4** | GGGGTTTTGGGGGGGGTTTTGGGG | [1] |
| **Seq5** | GGTGTGTGGTGGTGTGGTGGTGGTG | [2] |
| **Seq6** | GGTGTGTGTGTGGTGTGGTGGTGGTG | [2] |
| **Seq7** | GGTGTGTGTGTGTGTGTGGTGGTGGTG | [2] |
| **Seq8*** | GTGTGTGTGTGTGTGTGTGGTGGTGGTG | [2] |
| **Seq9** | GGGAGGGCGCGCCAGCGGGGTCGGG | [3] |
| **Seq10** | GCGGGAGGGCGCGCCAGCGGGGTCGGG | [3] |
| **Seq11** | TTGGATCTGAGAATCAGATGTGGGTGGGTGGGT | [4] |
| **Seq12** | GGTCTGAGGGAGAGGGGCTGGGT | [5] |
| **Seq13** | CGGGCGCGGGAGGAAGGGGGCGGGA | [6] |
| **Seq14** | GGGTGGGAAGGGTGGGA | [7] |
| **Seq15** | GGGATGGGACACAGGGGACGGG | [8] |
| **Seq16** | AGGGCGGTGTGGGAAGAGGGAAGAGGGGGAGG | [9] |
| **Seq17** | AGGGCGGTTTGGGAAGAGGGAAGAGGGGGAGG | [9] |
| **Seq18*** | GCGGAGGGGAGGCG | [10] |
| **Seq19*** | GCGGAGGGGAGG | [10] |
| **Seq20** | GCGTGGGTCAGGGTGGGTGGGACGC | [11] |
| **Seq21** | GGTTGGTGTGGTTGGTTGTGGTGGTGGTG | [12] |
| **Seq22** | GGGTTGGGTTGGGTTGGG | [13] |
| **Seq23** | TGAGGGTGGGTAGGGTGGGTAA | [14] |
| **Seq24** | TTGGGGAGGGTTTTAGGGTGGGGAAT | [15] |
| **Seq25** | GGGCCACCGGGCAGTGGGCGGG | [16] |
| **Seq26** | GGGTCACCGGGCAGTGGGCGGG | [16] |
| **Seq27** | TGGTGGTGGTGGTTGTGGTGGTGGTTGT | [17] |
| **Seq28*** | GGTTTTGGGGTTTTGGGG | [18] |
| **Seq29** | GGGTACCCGGGTGAGGGGCGGGGCT | [19] |
| **Seq30** | GGGTTAGGGTTAGGGTTTGGG | [20] |
| **Seq31** | GGGTTGCGGAGGGTGGGCCT | [21] |
| **Seq32*** | GCGAGGGAGCGAGGG | [22] |
| **Seq33** | GGTTTGGTTTTGGTTGG | [23] |
| **Seq34** | ACAGGGGTGTGGGGACAGGGGTGTGGGG | [24] |
| **Seq35** | AGAGTGGGGGGGATGTAGGTGGGTT | [24] |
| **Seq36** | AGCGGGAGAAGACGGGCTGGGAGGGCGC | [24] |
| **Seq37** | AGGGAAGAGAAAGGGGCCAGGGCCTGGGA | [24] |
| **Seq38** | AGGGAGGGCGCTGGGAGGAGGG | [24] |
| **Seq39** | AGGGATGGAACCGGGGAGGGCGGCGGGGGGGGGGGGGGGGT | [24] |
| **Seq40** | AGGGATTGGGATTGGGATTGGGTT | [24] |
| **Seq41** | AGGGCGGTGTGGGAAGAGGGAAGAGGGGGAGG | [24] |
| **Seq42** | AGGGCGGTGTGGGAAGAGGGAAGAGGGGGAGGCAG | [24] |
| **Seq43** | AGGGGGGAGGGAGGGTGG | [24] |
| **Seq44** | AGGGGTTAGGGGTTAGGGGTTAGGGG | [24] |
| **Seq45** | AGGGTCAGGGTCAGGGTCAGGG | [24] |
| **Seq46** | AGGGTGGGTGTAAGTGTGGGTGGGT | [24] |
| **Seq47** | AGTGGGGGTAGGGGATAGGGTAGGC | [24] |
| **Seq48** | ATCACGTGGTGGGCAAATAACCGGTTGGGGTGGGTCGAGG | [24] |
| **Seq49** | CAGGCGTTAGGGAAGGGCGTCGAAAGCAGGGTGGG | [24] |
| **Seq50** | CCCGGGACGGGGGCCGGCGGGCCACGGGCCC | [24] |
| **Seq51** | CCGTAGGTTCGGGGTCGGAGTGGTCCGGAAGGTGGCGTGG | [24] |
| **Seq52** | CCGTAGGTTCGGGGCGGAGTGGTCCGGAAGGTGGCGTGG | [24] |
| **Seq53** | CGGAGGGGTGGGGGAGGGGTGGGGGAGGGT | [24] |
| **Seq54** | CGGGAGGGCGGGGTGTGGTATGTATTGAGCGTGGTCCGTG | [24] |
| **Seq55** | CGGGCGGGCGCGAGGGAGGGGT | [24] |
| **Seq56** | CGGGGGGGGGGGGGGGGGCGGGGGAGGGAGGC | [24] |
| **Seq57** | GAAGGGACACGGAGGGGCGGGCCAGAGGGTCC | [24] |
| **Seq58** | GAGCGGGGACGAACACATATGGGGAAGTGGCTTGGGGTGG | [24] |
| **Seq59** | GAGGAGGGAGAATAGGGGTGGGTGG | [24] |
| **Seq60** | GAGTGCGTAATGGTACGATTTGGGAAGTGGCTTGGGGTGG | [24] |
| **Seq61** | GATACACGGGCGGAGGAGGTGGGGGGGGGTAGGTGGGTAT | [24] |
| **Seq62** | GATTGAAAGGTCTGTTTTTGGGGTTGGTTTGGGTCAATA | [24] |
| **Seq63** | GCCGGGCGGGGAGGGGGGGTCA | [24] |
| **Seq64** | GCCGGGGCGGCTCGGGACGGGGCCCGGGGAGCGTGGGTGGGACC | [24] |
| **Seq65** | GCGGGGTTGGGCGGGTGGGTTCGCTGGGCAGGGGGCGAGTG | [24] |
| **Seq66** | GCTGGGCGAGGGGTGGGAGCAGACGGGCTG | [24] |
| **Seq67** | GCTGGGGTGTTGGGTGTGGGGGTGA | [24] |
| **Seq68** | GCTGGGTTGGGGCGGGGGGAGCGGGACG | [24] |
| **Seq69** | GGAGGAACGGGTTCCAGTGTGGGGTCTATCGGGGCGTGCG | [24] |
| **Seq70** | GGAGGCGCGATGTAGGTATGTGAGGGCGGCGCGGTGGGCG | [24] |
| **Seq71** | GGAGGGAAAAGTTATCAGGCTGGATGGTAGCTCGGTCGGGGTGGGTGGGTTGGCAAGTCT | [24] |
| **Seq72** | GGCGAGGAGGGGCGTGGCCGGC | [24] |
| **Seq73** | GGGACGTAGTGGGGGGACGTAGTGGG | [24] |
| **Seq74** | GGGACTGGGACTGGGACTGGG | [24] |
| **Seq75** | GGGAGGGCGCTGGGAGGAGGG | [24] |
| **Seq76** | GGGAGGGTGTAAGTGTGGGAGGG | [24] |
| **Seq77** | GGGAGGGTGTAAGTGTGGGCGGG | [24] |
| **Seq78** | GGGAGGGTGTAAGTGTGGGTGGG | [24] |
| **Seq79** | GGGAGGGTTGGGGTGGG | [24] |
| **Seq80** | GGGAGGGTTTGGGAGGG | [24] |
| **Seq81** | GGGAGGGTTTGGGCGGG | [24] |
| **Seq82** | GGGAGGGTTTGGGTGGG | [24] |
| **Seq83** | GGGAGGGTTTTTTGGGAGGG | [24] |
| **Seq84** | GGGAGGGTTTTTTGGGCGGG | [24] |
| **Seq85** | GGGAGGGTTTTTTGGGTGGG | [24] |
| **Seq86** | GGGATTGGGATTGGGATTGGGTT | [24] |
| **Seq87** | GGGCAGCGGTGGTGTGGCGGGATCTGGGGTTGTGCGGTGT | [24] |
| **Seq88** | GGGCAGGGAGGGAACTGGG | [24] |
| **Seq89** | GGGCCTGTCAGGGTGGGCTAGGG | [24] |
| **Seq90** | GGGCCTGTCAGGGTTTGGGTTTGGG | [24] |
| **Seq91** | GGGCCTGTTGGGGTTTGGGTTTGGG | [24] |
| **Seq92** | GGGCCTGTTGGGTTTGGGTTTGGG | [24] |
| **Seq93** | GGGCCTTTCAGGGTTTGGGTTTGGG | [24] |
| **Seq94** | GGGCGCGGGAGGAAGGGGGCGGG | [24] |
| **Seq95** | GGGCGCGGGAGGAATTGGGCGGG | [24] |
| **Seq96** | GGGCGGGAUAGAGAGCGUGGGCGGG | [24] |
| **Seq97** | GGGCGGGCGCGAGGGAGGGG | [24] |
| **Seq98** | GGGCGGGCGGCTCCGGGCGCGGG | [24] |
| **Seq99** | GGGCGGGGAGGGGGAAGGGA | [24] |
| **Seq100** | GGGCGGGGGTGCTGGGGGTGGAGTGCTGCGTGCTGCGG | [24] |
| **Seq101** | GGGCGGGTGTAAGTGTGGGAGGG | [24] |
| **Seq102** | GGGCGGGTGTAAGTGTGGGCGGG | [24] |
| **Seq103** | GGGCGGGTGTAAGTGTGGGTGGG | [24] |
| **Seq104** | GGGCGGGTTTGGGAGGG | [24] |
| **Seq105** | GGGCGGGTTTGGGCGGG | [24] |
| **Seq106** | GGGCGGGTTTGGGTGGG | [24] |
| **Seq107** | GGGCGGGTTTTTTGGGAGGG | [24] |
| **Seq108** | GGGCGGGTTTTTTGGGCGGG | [24] |
| **Seq109** | GGGCGGGTTTTTTGGGTGGG | [24] |
| **Seq110** | GGGCGGTGTGGGAAGAGGGAAGAGGGG | [24] |
| **Seq111** | GGGCTAGGGCTAGGGCTAGGG | [24] |
| **Seq112** | GGGCTAGGGTGGGCCTGTCAGGG | [24] |
| **Seq113** | GGGGAAAAAGGGGGGGGGGGGGGGGGGGGGG | [24] |
| **Seq114** | GGGGAGGGGAAGGGGAGGG | [24] |
| **Seq115** | GGGGGAGGACGCGTAGTGGGGGGCCCATGGTTGTGTGG | [24] |
| **Seq116** | GGGGTAGGATAGGGTNTGGAAGGAGGTGCCCCGT | [24] |
| **Seq117** | GGGGTCTGGGTGCTGTGGGGTCTGGG | [24] |
| **Seq118** | GGGGTGGGAGGAGGGT | [24] |
| **Seq119** | GGGGTTACCGCAAAATGGATAGATGGACCGGGGCACACCGGGTAGGGGTCCGGAGGG | [24] |
| **Seq120** | GGGGTTGGGGTTGGGGTTGGGG | [24] |
| **Seq121** | GGGGTTTTGGGGTTTTGGGGTTTTGGGG | [24] |
| **Seq122** | GGGTACGGTGGGTAATAAGGGAAGGTATCGGG | [24] |
| **Seq123** | GGGTAGCATTGGGTTTGGGTTTGGG | [24] |
| **Seq124** | GGGTAGGGTAGGGTAGGG | [24] |
| **Seq125** | GGGTCAGGGTCAGGGTCAGGG | [24] |
| **Seq126** | GGGTCCTCCAAGGGGTAAAACTTACATGGGATGGTGGGGTCACATGGG | [24] |
| **Seq127** | GGGTGGGCCTGTCAGGGCTAGGG | [24] |
| **Seq128** | GGGTGGGTGGGTGGGT | [24] |
| **Seq129** | GGGTGGGTGGGTGGGT | [24] |
| **Seq130** | GGGTGGGTGTAAGTGTGGGAGGG | [24] |
| **Seq131** | GGGTGGGTGTAAGTGTGGGCGGG | [24] |
| **Seq132** | GGGTGGGTGTAAGTGTGGGTGGG | [24] |
| **Seq133** | GGGTGGGTTTGGGAGGG | [24] |
| **Seq134** | GGGTGGGTTTGGGCGGG | [24] |
| **Seq135** | GGGTGGGTTTGGGTGGG | [24] |
| **Seq136** | GGGTGGGTTTTTTGGGAGGG | [24] |
| **Seq137** | GGGTGGGTTTTTTGGGCGGG | [24] |
| **Seq138** | GGGTGGGTTTTTTGGGTGGG | [24] |
| **Seq139** | GGGTGGGTTTTTTTTTGGGTGGG | [24] |
| **Seq140** | GGGTGGGTTTTTTTTTTTTTTTGGGTGGG | [24] |
| **Seq141** | GGGTGGGTTTTTTTTTTTTTTTTTTGGGTGGG | [24] |
| **Seq142** | GGGTGGGTTTTTTTTTTTTTTTTTTTTTGGGTGGG | [24] |
| **Seq143** | GGGTGGGTTTTTTTTTTTTTTTTTTTTTTTTGGGTGGG | [24] |
| **Seq144** | GGGTGGGTTTTTTTTTTTTTTTTTTTTTTTTTTTTTTGGGTGGG | [24] |
| **Seq145** | GGGTGTGAGAGGTTGAGGGGGTTCG | [24] |
| **Seq146** | GGGTGTGGGAGGTGATGGGGTAGGT | [24] |
| **Seq147** | GGGTGTGGGTGTGGGTGTGGG | [24] |
| **Seq148** | GGGTGTGTGGGTGTGTGGGTGTGTGGG | [24] |
| **Seq149** | GGGTTAAGGGTTAAGGGTTAAGGG | [24] |
| **Seq150** | GGGTTAGGGTTAGGGTTAGGG | [24] |
| **Seq151** | GGGTTAGGGTTAGGGTTAGGGT | [24] |
| **Seq152** | GGGTTAGGGTTAGGGTTAGGGTTA | [24] |
| **Seq153** | GGGTTAGGGTTAGGGTTAGGGTTAGGGTTAGGGTTAGGGTTAGGG | [24] |
| **Seq154** | GGGTTCAGGGTTCAGGGTTCAGGG | [24] |
| **Seq155** | GGGTTGGGTGTGGGTTGGG | [24] |
| **Seq156** | GGGTTGGGTTAGGGTTGGG | [24] |
| **Seq157** | GGGTTGGGTTTTGGGTTGGG | [24] |
| **Seq158** | GGGTTTAGGGTTTAGGGTTTAGGG | [24] |
| **Seq159** | GGGTTTGGGAAAGGGTTTGGG | [24] |
| **Seq160** | GGGTTTGGGAACGGGTTTGGG | [24] |
| **Seq161** | GGGTTTGGGAATGGGTTTGGG | [24] |
| **Seq162** | GGGTTTGGGACAGGGTTTGGG | [24] |
| **Seq163** | GGGTTTGGGACCGGGTTTGGG | [24] |
| **Seq164** | GGGTTTGGGACTGGGTTTGGG | [24] |
| **Seq165** | GGGTTTGGGAGAGGGTTTGGG | [24] |
| **Seq166** | GGGTTTGGGAGCGGGTTTGGG | [24] |
| **Seq167** | GGGTTTGGGAGTGGGTTTGGG | [24] |
| **Seq168** | GGGTTTGGGATAGGGTTTGGG | [24] |
| **Seq169** | GGGTTTGGGATCGGGTTTGGG | [24] |
| **Seq170** | GGGTTTGGGATTGGGTTTGGG | [24] |
| **Seq171** | GGGTTTGGGCAAGGGTTTGGG | [24] |
| **Seq172** | GGGTTTGGGCACGGGTTTGGG | [24] |
| **Seq173** | GGGTTTGGGCATGGGTTTGGG | [24] |
| **Seq174** | GGGTTTGGGCCAGGGTTTGGG | [24] |
| **Seq175** | GGGTTTGGGCCCGGGTTTGGG | [24] |
| **Seq176** | GGGTTTGGGCCTGGGTTTGGG | [24] |
| **Seq177** | GGGTTTGGGCCTGTCAGGGTTTGGG | [24] |
| **Seq178** | GGGTTTGGGCCTGTTGGGGTTTGGG | [24] |
| **Seq179** | GGGTTTGGGCCTGTTGGGTTTGGG | [24] |
| **Seq180** | GGGTTTGGGCCTTTCAGGGTTTGGG | [24] |
| **Seq181** | GGGTTTGGGCGAGGGTTTGGG | [24] |
| **Seq182** | GGGTTTGGGCGCGGGTTTGGG | [24] |
| **Seq183** | GGGTTTGGGCGTGGGTTTGGG | [24] |
| **Seq184** | GGGTTTGGGCTAGGGTTTGGG | [24] |
| **Seq185** | GGGTTTGGGCTCGGGTTTGGG | [24] |
| **Seq186** | GGGTTTGGGCTTGGGTTTGGG | [24] |
| **Seq187** | GGGTTTGGGTAAGGGTTTGGG | [24] |
| **Seq188** | GGGTTTGGGTACGGGTTTGGG | [24] |
| **Seq189** | GGGTTTGGGTAGCATTGGGTTTGGG | [24] |
| **Seq190** | GGGTTTGGGTATGGGTTTGGG | [24] |
| **Seq191** | GGGTTTGGGTCAGGGTTTGGG | [24] |
| **Seq192** | GGGTTTGGGTCCGGGTTTGGG | [24] |
| **Seq193** | GGGTTTGGGTCTGGGTTTGGG | [24] |
| **Seq194** | GGGTTTGGGTGAGGGTTTGGG | [24] |
| **Seq195** | GGGTTTGGGTGCGGGTTTGGG | [24] |
| **Seq196** | GGGTTTGGGTGGGTTTGGG | [24] |
| **Seq197** | GGGTTTGGGTGTGGGTTTGGG | [24] |
| **Seq198** | GGGTTTGGGTTAGGGTTTGGG | [24] |
| **Seq199** | GGGTTTGGGTTCGGGTTTGGG | [24] |
| **Seq200** | GGGTTTGGGTTGGGTTTGGG | [24] |
| **Seq201** | GGGTTTGGGTTTGGGCCTGTCAGGG | [24] |
| **Seq202** | GGGTTTGGGTTTGGGCCTGTTGGG | [24] |
| **Seq203** | GGGTTTGGGTTTGGGCCTGTTGGGG | [24] |
| **Seq204** | GGGTTTGGGTTTGGGCCTTTCAGGG | [24] |
| **Seq205** | GGGTTTGGGTTTGGGTTTGGG | [24] |
| **Seq206** | GGGTTTGGGTTTTGGGTTTGGG | [24] |
| **Seq207** | GGGTTTGGGTTTTTGGGTTTGGG | [24] |
| **Seq208** | GGGTTTGGGTTTTTTGGGTTTGGG | [24] |
| **Seq209** | GGGTTTGGGTTTTTTTGGGTTTGGG | [24] |
| **Seq210** | GGGTTTGGGTTTTTTTTTGGGTTTGGG | [24] |
| **Seq211** | GGGTTTGGGTTTTTTTTTTTTTTTGGGTTTGGG | [24] |
| **Seq212** | GGGTTTGGGTTTTTTTTTTTTTTTTTTTTTGGGTTTGGG | [24] |
| **Seq213** | GGGTTTGGGTTTTTTTTTTTTTTTTTTTTTTTTTTTTTTGGGTTTGGG | [24] |
| **Seq214** | GGGTTTTAGGGTTTTAGGGTTTTAGGG | [24] |
| **Seq215** | GGTTAGGTTAGGTTAGG | [24] |
| **Seq216** | GGTTGGATGTAAGGTTGGAGGGGGG | [24] |
| **Seq217** | GGTTGGTGTGGTTGG | [24] |
| **Seq218** | GGTTGGTTAGGTTGG | [24] |
| **Seq219** | GGTTGGTTTGGTTGG | [24] |
| **Seq220** | GGTTGGTTTTGGTTGG | [24] |
| **Seq221** | GTAGGTGGGGGACTGGGGACGGGTATGGGCACACGGTAT | [24] |
| **Seq222** | GTCAAGGTGGGTGGGTGGGGTTGGTTGTTGTTTTGA | [24] |
| **Seq223** | GTGGGTAGGGCGGGTTGG | [24] |
| **Seq224** | GTGGTGGGTGGGTGGGT | [24] |
| **Seq225** | TAAGGGTGGGTGTAAGTGTGGGTGGGT | [24] |
| **Seq226** | TAAGGGTGGGTGTAAGTGTGGGTGGGTGT | [24] |
| **Seq227** | TAGCGGGTGTGGTGGGTGGGGGAGGCATGGTTTTTGGTAA | [24] |
| **Seq228** | TAGGGTTAGGGTTAGGGTTAGGGATT | [24] |
| **Seq229** | TATGGGGGTGGGTCAGGTTTCGGTA | [24] |
| **Seq230** | TCAGGGTGGGGGCCCCGAGGGCTGGGGCCG | [24] |
| **Seq231** | TCCGGGGGGCTGGGCAGGGGGGTAACTTTC | [24] |
| **Seq232** | TCGAGGGGTGTGCAAGGCGGGTCAACGGGCCTTATTTGGTGCTTAGGTA | [24] |
| **Seq233** | TGAGGGTCTAGGGTGGTGGGGTGGA | [24] |
| **Seq234** | TGAGGGTGGTGAGGGTGGGGAAGG | [24] |
| **Seq235** | TGATGGATGTGGGGATGCGGGGGCG | [24] |
| **Seq236** | TGGCCTGGGCGGGACTGGG | [24] |
| **Seq237** | TGGCTAGTGGGTAAGGGGCGGGAGGGTGACAGGGCGATCC | [24] |
| **Seq238** | TGGGCAGGGGAGACACTGGGATCTGAGGGTCTGGGT | [24] |
| **Seq239** | TGGGCCAAGGGCAGAGAAGGGCTGGGA | [24] |
| **Seq240** | TGGGCCGTGTGGGCAGAGGGCACATGGGC | [24] |
| **Seq241** | TGGGCGGGGGAGGGAGGGAGGGAGGGGGTTGAGGGTGGGGATGCCAAGGGGT | [24] |
| **Seq242** | TGGGGAGGGTGGGGAGGGTGGGGAAGG | [24] |
| **Seq243** | TGGGGGATGGGGTTGGAATGGGGGCGGGA | [24] |
| **Seq244** | TGGGGGCGGGGAGGGAAGGGGGT | [24] |
| **Seq245** | TGGGGGGAGGGGCGGAGGGGTGGGGTCGCGCGGGT | [24] |
| **Seq246** | TGGGGGTCGGGATACGGTCAGTGGTGGTGAGTGGTAACGG | [24] |
| **Seq247** | TGGGGGTTGGGGGTTGGGGGTTGGGGGT | [24] |
| **Seq248** | TGGGGTGGGGGTGGAAAGGGGGTGGGGGTGGT | [24] |
| **Seq249** | TGGGGTGGGGGTGGGGGTGGTGGTGGGGC | [24] |
| **Seq250** | TGGGGTTGGGGTTGGGGTTGGGGT | [24] |
| **Seq251** | TGGGTAGGTTCGAGGGGTGGGTGTG | [24] |
| **Seq252** | TGGGTGGGTTTAATTTTGGGTGGGA | [24] |
| **Seq253** | TGGGTGTAAGTGTGGGTGGGTGTAATTGTGGGT | [24] |
| **Seq254** | TGGTTGGGGATAGAGGTGGGTGTTT | [24] |
| **Seq255** | TGTGAAGGGGGGTACATGACGGGGACTGGCCGGACTACAG | [24] |
| **Seq256** | TGTGGGGTAGGGGGAGGGGGGAGGGATA | [24] |
| **Seq257** | TGTGGGGTCGGGGGAGGGGGGAGGGATA | [24] |
| **Seq258** | TGTGGTGGGTGGGTGGGT | [24] |
| **Seq259** | TTGGGGTGGGAGGGCGGGTTAACAAAGATAGCGCAACAGG | [24] |
| **Seq260** | TTGGGTGGGTGGGTGGGT | [24] |
| **Seq261** | TTGGGTTAGGGTTAGGGTTAGGGA | [24] |
| **Seq262** | TTGTGGTGGGTGGGTGGGT | [24] |
| **Seq263** | TTTGTGGGTGTGGGTGTGGGTGTGGG | [24] |
| **Seq264** | GGGGCTGGGGCTGGGGCTGGGG | [24] |
| **Seq265** | AGCGGGGGAGAAUUAGAUAAAUGGGAAAAAAUUCGGUUAAGGCCAGGGGGAAA | [24] |
| **Seq266** | TTTTAAAAGAAAAGGGGGGATTGGGGGGTACAGTGCAGGGG | [24] |
| **Seq267*** | TTTTAAAAGAAGGGGAGGAATAGGGGATATGA | [24] |
| **Seq268** | GGGACTTTCCGCTGGGGACTTTCCAGGGAGGCGTGGCCTGGGCGGGACTGGGGAGTGG | [24] |
| **Seq269** | GAGGAGGAGGTGGGT | [24] |
| **Seq270** | GGTCTTAAAGGTACCTGAGGTCTGACTGG | [24] |
| **Seq271** | GGGGGGACTGGAAGGG | [24] |
| **Seq272** | GGGCTTGGGTGGGCGCTTGGG | [24] |
| **Seq273** | GGGAGTGGGAGCGGGAACGGGAACGGGACTGGGA | [24] |
| **Seq274** | CGGGAACGGGAACGGGACTGGGA | [24] |
| **Seq275** | GTGGGAGCGGGAACGGGAACGGGA | [24] |
| **Seq276** | CCGGGAGTGGGAGCGGGAACGGGA | [24] |
| **Seq277** | GGGAGCGGGACTGGGACCGGGACCGGGACCGGG | [24] |
| **Seq278** | GGGAGCGGGACTGGGACCGGGACCGGGA | [24] |
| **Seq279** | GGGAGCGGGACTGGGACCGGGA | [24] |
| **Seq280** | GGGACTGGGACCGGGACCGGGA | [24] |
| **Seq281** | GGGAGTATGGGTAACGGGGGGGG | [24] |
| **Seq282** | GGGACTATGGGTAACGGGGGGG | [24] |
| **Seq283** | GGGTAGGGCAGGGGACACAGGGTAGGG | [24] |
| **Seq284** | GGGCAGGGGACACAGGGTAGGG | [24] |
| **Seq285** | GGGTAGGGCAGGGGACACAGGGT | [24] |
| **Seq286** | GGGAAAGGGTACCTCGAGGGGCCGCGGGG | [24] |
| **Seq287** | GGGCAGGGTAGGGCAATTTAGGG | [24] |
| **Seq288** | GGGTTAGGGTTAGGGTTAGGG | [24] |
| **Seq289** | GGATGGGGTGGGGAGG | [24] |
| **Seq290*** | AGATGGAGTGGAGAGG | [24] |
| **Seq291** | GGGGGATGCGGGGG | [24] |
| **Seq292*** | AGGAGATGCAGGAG | [24] |
| **Seq293** | GGAGGGTGGATGG | [24] |
| **Seq294*** | AGAGGGTAGATGG | [24] |
| **Seq295** | GGGGCCGGGGCCGGGGCCGGGGC | [24] |
| **Seq296** | TGGGTAGGGCGGGTTGGGAAA | [24] |
| **Seq297** | TTGGGTGGGTGGGTGGGT | [24] |
| **Seq298** | GGTGGTGGTGGTTGTGGTGGTGGTGG | [24] |
| **Seq299** | GAAGCGGGGGAGGGGGGGUUUGGUGGAAAU | [24] |
| **Seq300** | GGGGTGAAAGGGGCCCTGGGCTTGGG | [24] |
| **Seq301** | GGGGGCCTTGGGGCTCGGCAGGGGTGAAAGGGG | [24] |
| **Seq302** | GGGGTAGGTGGGGATCTGTGGGATTGG | [24] |
| **Seq303** | AGGGGAGGGGCTGGGAGGGC | [24] |
| **Seq304** | AGGGGTCGGGTCGGGGCGGGGT | [24] |
| **Seq305** | GGGTTTTTTGGGTTTTTTGGGTTTTTTGGG | [24] |
| **Seq306** | GGGGTTTTTTGGGGTTTTTTGGGGTTTTTTGGG | [24] |
| **Seq307** | GGGGTTTTTGGGGTTTTTGGGGTTTTTGGG | [24] |
| **Seq308** | TCACAGGGTTTTTTGGGTTTTTTGGGTTTTTTGGGACAA | [24] |
| **Seq309** | TCACAGGGGTTTTTTGGGGTTTTTTGGGGTTTTTTGGGGACAA | [24] |
| **Seq310** | TCACAGGGGTTTTTGGGGTTTTTGGGGTTTTTGGGGACAA | [24] |
| **Seq311** | TGGGTGGGACTATTGGGACGGGT | [24] |
| **Seq312** | TGGGTGGGACTATTGGGACAGGGC | [24] |
| **Seq313** | TGGGGCTGGGGTTACGGGGCCAGTGGGGT | [24] |
| **Seq314** | TGGGACCATTGAGGGTGGGAAATTGGACAATGGGGA | [24] |
| **Seq315** | CGGGGTCCGAGGGGATTCCTAAGGGGGTTCTGGGGA | [24] |
| **Seq316** | CGGGCGGGTGGGTTGGCCGAAGGGT | [24] |
| **Seq317** | CGGGCGGGTGGGTTGGCCGAGGGT | [24] |
| **Seq318** | CGGGCTCACGGGTGGGTATGGGC | [24] |
| **Seq319** | AGGGCGGGACTGAGGGCGGGGC | [24] |
| **Seq320** | CGGGGTGGGCGGGGGGCGAGGGA | [24] |
| **Seq321** | CGGGGTGGTGGGCCCAGGGATTGTTAGCGGGT | [24] |
| **Seq322** | AGGGACATGGGTGGGAGGGA | [24] |
| **Seq323** | AGGGCGGGGCCGCGGAAAGGAAGGGGAGGGGC | [24] |
| **Seq324** | CGGGGCCGCGGAAAGGAAGGGGAGGGGCTGGGA | [24] |
| **Seq325** | TGGGAGGGCCCGGAGGGGGCTGGGC | [24] |
| **Seq326** | AGGGCCCGGAGGGGGCTGGGCCGGGGA | [24] |
| **Seq327** | AGGGGGCTGGGCCGGGGACCCGGGA | [24] |
| **Seq328** | CGGGAGGGGTCGGGACGGGGC | [24] |
| **Seq329** | GGGGTAGGTGGGGATCTGTGGGATTGG | [24] |

References:

[1] K. Li, L. Yatsunyk, S. Neidle, Water spines and networks in G-quadruplex structures, Nucleic Acids Research. 49 (2021) 519–528.

[2] P. Das, K.H. Ngo, F.R. Winnerdy, A. Maity, B. Bakalar, Y. Mechulam, E. Schmitt, A.T. Phan, Bulges in left-handed G-quadruplexes, Nucleic Acids Research. 49 (2021) 1724–1736.

[3] D.J. Tan, F.R. Winnerdy, K.W. Lim, A.T. Phan, Coexistence of two quadruplex–duplex hybrids in the PIM1 gene, Nucleic Acids Research. 48 (2020) 11162–11171.

[4] T.Q. Ngoc Nguyen, K.W. Lim, A.T. Phan, Duplex formation in a G-quadruplex bulge, Nucleic Acids Research. 48 (2020) 10567–10575.

[5] Y.M. Vianney, K. Weisz, First Tandem Repeat of a Potassium Channel KCNN4 Minisatellite Folds into a V-Loop G-Quadruplex Structure, Biochemistry. 60 (2021) 1337–1346.

[6] S. Bielskutė, J. Plavec, P. Podbevšek, Oxidative lesions modulate G-quadruplex stability and structure in the human BCL2 promoter, Nucleic Acids Research. 49 (2021) 2346–2356.

[7] M. Volek, S. Kolesnikova, K. Svehlova, P. Srb, R. Sgallová, T. Streckerová, J.A. Redondo, V. Veverka, E.A. Curtis, Overlapping but distinct: a new model for G-quadruplex biochemical specificity, Nucleic Acids Research. 49 (2021) 1816–1827.

[8] L. Haase, K. Weisz, Locked nucleic acid building blocks as versatile tools for advanced G-quadruplex design, Nucleic Acids Research. 48 (2020) 10555–10566.

[9] J. Marquevielle, C. Robert, O. Lagrabette, M. Wahid, A. Bourdoncle, L.E. Xodo, J.-L. Mergny, G.F. Salgado, Structure of two G-quadruplexes in equilibrium in the KRAS promoter, Nucleic Acids Research. 48 (2020) 9336–9345.

[10] D. Pavc, B. Wang, L. Spindler, I. Drevenšek-Olenik, J. Plavec, P. Šket, GC ends control topology of DNA G-quadruplexes and their cation-dependent assembly, Nucleic Acids Research. 48 (2020) 2749–2761.

[11] B. Karg, S. Mohr, K. Weisz, Duplex‐guided refolding into novel G‐quadruplex (3+ 1) hybrid conformations, Angewandte Chemie International Edition. 58 (2019) 11068–11071.

[12] F.R. Winnerdy, B. Bakalar, A. Maity, J.J. Vandana, Y. Mechulam, E. Schmitt, A.T. Phan, NMR solution and X-ray crystal structures of a DNA molecule containing both right-and left-handed parallel-stranded G-quadruplexes, Nucleic Acids Research. 47 (2019) 8272–8281.

[13] L.Y. Lin, S. McCarthy, B.M. Powell, Y. Manurung, I.M. Xiang, W.L. Dean, B. Chaires, L.A. Yatsunyk, Biophysical and X-ray structural studies of the (GGGTT) 3GGG G-quadruplex in complex with N-methyl mesoporphyrin IX, PLoS One. 15 (2020) e0241513.

[14] W. Liu, C. Lin, G. Wu, J. Dai, T.-C. Chang, D. Yang, Structures of 1: 1 and 2: 1 complexes of BMVC and MYC promoter G-quadruplex reveal a mechanism of ligand conformation adjustment for G4-recognition, Nucleic Acids Research. 47 (2019) 11931–11942.

[15] J. Dickerhoff, B. Onel, L. Chen, Y. Chen, D. Yang, Solution structure of a MYC promoter G-quadruplex with 1: 6: 1 loop length, ACS Omega. 4 (2019) 2533–2539.

[16] Z.-F. Wang, M.-H. Li, I.-T. Chu, F.R. Winnerdy, A.T. Phan, T.-C. Chang, Cytosine epigenetic modification modulates the formation of an unprecedented G4 structure in the WNT1 promoter, Nucleic Acids Research. 48 (2020) 1120–1130.

[17] A. Maity, F.R. Winnerdy, W.D. Chang, G. Chen, A.T. Phan, Intra-locked G-quadruplex structures formed by irregular DNA G-rich motifs, Nucleic Acids Research. 48 (2020) 3315–3327.

[18] C. Wan, W. Fu, H. Jing, N. Zhang, NMR solution structure of an asymmetric intermolecular leaped V-shape G-quadruplex: selective recognition of the d (G2NG3NG4) sequence motif by a short linear G-rich DNA probe, Nucleic Acids Research. 47 (2019) 1544–1556.

[19] Y. Liu, W. Lan, C. Wang, C. Cao, A putative G-quadruplex structure in the proximal promoter of VEGFR-2 has implications for drug design to inhibit tumor angiogenesis, Journal of Biological Chemistry. 293 (2018) 8947–8955.

[20] C. Liu, B. Zhou, Y. Geng, D.Y. Tam, R. Feng, H. Miao, N. Xu, X. Shi, Y. You, Y. Hong, A chair-type G-quadruplex structure formed by a human telomeric variant DNA in K+ solution, Chemical Science. 10 (2019) 218–226.

[21] M. Meier, A. Moya-Torres, N.J. Krahn, M.D. McDougall, G.L. Orriss, E.K.S. McRae, E.P. Booy, K. McEleney, T.R. Patel, S.A. McKenna, Structure and hydrodynamics of a DNA G-quadruplex with a cytosine bulge, Nucleic Acids Research. 46 (2018) 5319–5331.

[22] V. Kocman, J. Plavec, Tetrahelical structural family adopted by AGCGA-rich regulatory DNA regions, Nature Communications. 8 (2017) 15355.

[23] S.A. Dvorkin, A.I. Karsisiotis, M. Webba da Silva, Encoding canonical DNA quadruplex structure, Science Advances. 4 (2018) eaat3007.

[24] A. Bedrat, L. Lacroix, J.-L. Mergny, Re-evaluation of G-quadruplex propensity with G4Hunter, Nucleic Acids Research. 44 (2016) 1746–1759.
